# Statistical analysis of high-dimensional biomedical data: a gentle introduction to analytical goals, common approaches and challenges

**DOI:** 10.1186/s12916-023-02858-y

**Published:** 2023-05-15

**Authors:** Jörg Rahnenführer, Riccardo De Bin, Axel Benner, Federico Ambrogi, Lara Lusa, Anne-Laure Boulesteix, Eugenia Migliavacca, Harald Binder, Stefan Michiels, Willi Sauerbrei, Lisa McShane, for topic group “High-dimensional data” (TG9) of the STRATOS initiative

**Affiliations:** 1 Department of Statistics, TU Dortmund University, Dortmund, Germany; 2 Department of Mathematics, University of Oslo, Oslo, Norway; 3 Division of Biostatistics, German Cancer Research Center (DKFZ), Heidelberg, Germany; 4 Department of Clinical Sciences and Community Health, University of Milan, Milan, Italy; 5 Scientific Directorate, IRCCS Policlinico San Donato, San Donato Milanese, Italy; 6 Department of Mathematics, Faculty of Mathematics, Natural Sciences and Information Technology, University of Primorksa, Koper, Slovenia; 7 Institute of Biostatistics and Medical Informatics, University of Ljubljana, Ljubljana, Slovenia; 8 Institute for Medical Information Processing, Biometry and Epidemiology, Ludwig Maximilian University of Munich, Munich, Germany; 9 Nestle Research, EPFL Innovation Park, Lausanne, Switzerland; 10 Institute of Medical Biometry and Statistics, Faculty of Medicine and Medical Center, University of Freiburg, Freiburg, Germany; 11 Service de Biostatistique et d’Épidémiologie, Gustave Roussy, Université Paris-Saclay, Villejuif, France; 12 Oncostat U1018, Inserm, Université Paris-Saclay, Labeled Ligue Contre le Cancer, Villejuif, France; 13 Biometric Research Program, Division of Cancer Treatment and Diagnosis, National Cancer Institute, Bethesda, MD USA

**Keywords:** High-dimensional data, Omics data, STRATOS initiative, Analytical goals, Initial data analysis, Exploratory data analysis, Clustering, Multiple testing, Prediction

## Abstract

**Background:**

In high-dimensional data (HDD) settings, the number of variables associated with each observation is very large. Prominent examples of HDD in biomedical research include omics data with a large number of variables such as many measurements across the genome, proteome, or metabolome, as well as electronic health records data that have large numbers of variables recorded for each patient. The statistical analysis of such data requires knowledge and experience, sometimes of complex methods adapted to the respective research questions.

**Methods:**

Advances in statistical methodology and machine learning methods offer new opportunities for innovative analyses of HDD, but at the same time require a deeper understanding of some fundamental statistical concepts. Topic group TG9 “High-dimensional data” of the STRATOS (STRengthening Analytical Thinking for Observational Studies) initiative provides guidance for the analysis of observational studies, addressing particular statistical challenges and opportunities for the analysis of studies involving HDD. In this overview, we discuss key aspects of HDD analysis to provide a gentle introduction for non-statisticians and for classically trained statisticians with little experience specific to HDD.

**Results:**

The paper is organized with respect to subtopics that are most relevant for the analysis of HDD, in particular initial data analysis, exploratory data analysis, multiple testing, and prediction. For each subtopic, main analytical goals in HDD settings are outlined. For each of these goals, basic explanations for some commonly used analysis methods are provided. Situations are identified where traditional statistical methods cannot, or should not, be used in the HDD setting, or where adequate analytic tools are still lacking. Many key references are provided.

**Conclusions:**

This review aims to provide a solid statistical foundation for researchers, including statisticians and non-statisticians, who are new to research with HDD or simply want to better evaluate and understand the results of HDD analyses.

## Background

The goal of the topic group TG9 “High-dimensional data” (HDD) of the STRATOS (STRengthening Analytical Thinking for Observational Studies) [1] initiative is to provide guidance for planning, conducting, analyzing, and reporting studies involving high-dimensional biomedical data. The increasing availability and use of “big” data in biomedical research, characterized by “large *n*” (independent observations) and/or “large *p*” (number of dimensions of a measurement or number of variables associated with each independent observation), has created a need for the development and novel application of statistical methods and computational algorithms. Either large *n* or *p* may present difficulties for data storage or computations, but large *p* presents several major statistical challenges and opportunities [2]. The dimension p can range from several dozen to millions. The situation of very large *p* is the focus of TG9 and this paper. Throughout the paper, “*p*” will refer to the number of variables and the term “subject” will be used broadly to refer to independent observations, including human or animal subjects, or biospecimens derived from them; or other independent experimental or observational units. Researchers who design and analyze such studies need a basic understanding of the commonly used analysis methods and should be aware of pitfalls when statistical methods that are established in the low-dimensional setting cannot, or should not, be used in the HDD setting.

This overview, a product of STRATOS topic group TG9, provides a gentle introduction to fundamental concepts in the analysis of HDD, in the setting of observational studies in biomedical research. The focus is on analytical methods; however, issues related to study design, interpretation, transportability of findings, and clinical usefulness of results should also be considered as briefly discussed throughout this paper.

### The STRATOS initiative and the STRATOS topic group TG9 “High-dimensional data”

The STRATOS initiative (www.stratos-initiative.org) is a large collaboration involving experts in many different areas of biostatistical research. The objective of STRATOS is to provide accessible and sound guidance for the design and analysis of observational studies [1]. This guidance is intended for applied statisticians and other data analysts with varying levels of statistical training, experience and interests. TG9 is one of nine topic groups of STRATOS and deals with aspects of HDD analysis.

Main issues addressed by TG9 often overlap with those of other TGs, but in the work of TG9 there is always a focus on the HDD aspect. Sometimes TG9 guidance will build upon that of other TGs to adapt it for relevance to HDD (see the “Discussion” section), but also completely new issues arise and require novel statistical approaches.

High-dimensional data are now ubiquitous in biomedical research, very frequently in the context of observational studies. Particularly omics data, i.e., high-throughput molecular data (e.g., genomics, transcriptomics, proteomics, and metabolomics) have provided new insights into biological processes and disease pathogenesis and have furthered the development of precision medicine approaches [3]. Rapidly expanding stores of electronic health records contain not only standard demographic, clinical, and laboratory data collected through a patient history, but also information from potentially many different providers involved in a patient’s care [4]. Data may be derived from multiple sources and can be represented in many different forms. Collectively, these data can be leveraged to support programs in comparative effectiveness and health outcomes research, and to monitor public health. Many statistical methods that are discussed here may be applied to health records data as well as to omics data, but our primary focus here is on the analysis of omics data.

Simultaneously, advances in statistical methodology and machine learning methods have contributed to improved approaches for data mining, statistical inference, and prediction in the HDD setting. Strong collaborations between data and computational scientists (e.g., statisticians, computational biologists, bioinformaticians, and computer scientists) and other biomedical scientists (e.g., clinicians and biologists) are essential for optimal generation, management, processing, analysis, and interpretation of these high-dimensional biomedical data [5].

Credibility and importance of research findings from biomedical studies involving HDD can be better judged when there is understanding of various approaches for statistical design and analysis along with their strengths and weaknesses. While this overview directly aims to improve understanding, simultaneously this guidance implies what information is necessary to report to fully appreciate how a study was designed, conducted, and analyzed. Whether study results prompt further pre-clinical or early clinical work, or translation to clinical use, ability to judge quality, credibility, and relevance of those results is critical. It is important to avoid sending research programs down unproductive paths or allowing flawed research results such as poorly performing prognostic models or therapy selection algorithms generated from HDD to be implemented clinically [6]. Historically, research involving biomarkers and prognostic modelling has been criticized for lack of rigor, reproducibility, and clinical relevance [7–10], and for poor reporting [11, 12]. At least as many deficiencies are also common in biomedical research involving HDD. The goal of STRATOS TG9 is to reduce these deficiencies, and improve rigor and reproducibility, by providing widely accessible didactic materials pertinent to studies involving HDD.

### Study design

In any observational study, including in the HDD setting, study design plays a crucial role in relation to the research question. A first important point is the precise definition of the target population and the sampling procedure. The subjects included in a study (or biospecimens derived from them) may be selected from the population by a random or other statistically designed sampling procedure (e.g., case–control, case-cohort), or may simply represent a “convenience” sample. It is therefore important to understand whether the subjects are representative of the target population, how the variables associated with subjects were measured or ascertained, and whether there are potential confounding factors. Failure to account for confounding factors or minimize bias in subject or variable ascertainment can lead to useless or misleading results.

Outcome-dependent sampling is rather common in observational studies, particularly for those investigating risk factors for relatively uncommon diseases or outcomes. Examples include classical matched or unmatched case–control designs along with two-phase sampling from a cohort (case-cohort or nested case–control). Another often-used strategy oversamples long survivors, or, for continuous outcomes, subjects with high and low values of the outcome variable. When any such sampling strategies are employed, it is important to use inferential procedures [13, 14] that properly account for the sampling design.

Laboratory experiments generating high-dimensional assay data should adhere to the same best practices as traditional controlled experiments measuring only one or a few analytes, including randomization, replication, blocking, and quality monitoring. Arguably, careful design might be even more important in the setting of HDD generation because HDD assays may be especially sensitive to technical artifacts. Even when a study is primarily observational yet involves analysis of stored biospecimens using omics assays, good design principles should be followed when performing the assays. Best practices include randomizing biospecimens to assays batches to avoid confounding assay batch effects with other factors of interest. For unmatched case–control studies, balancing (randomizing) cases and controls into batches may provide important advantages for reducing the influence of batch effects [15]. For matched case–control studies or studies involving analysis of serial specimens from each subject, grouping matched or longitudinal sets within the same assay batch can be a convenient way to control for batch effects.

Another fundamental aspect of design is sample size, which refers to the measurement of different subjects, which are referred to as biological replicates. Whenever there is interest in making inference beyond an individual subject, e.g., assessing differential gene expression between groups of subjects with different phenotypes or exposed to different conditions such as treatments, biological replicates are required. In the HDD setting, standard sample size calculations generally do not apply. If statistical tests are performed one variable at a time (e.g., differential expression of each gene comparing two groups), then the number of tests performed for HDD is typically so large that a sample size calculation applying stringent multiplicity adjustment would lead to an enormous sample size. Alternative approaches to controlling false positive findings in HDD studies are discussed in section “TEST: Identification of informative variables and multiple testing.” If the goal is to develop a risk or prognostic model using HDD, typical recommendations about the number of events required per variable break down [16]. Other sample size methods that require assumptions about the model are challenging to implement considering the complexity of models that might be used in HDD settings [17, 18], as discussed in section “PRED2.4: Sample size considerations.” In reality, HDD studies are often conducted with inadequate sample size, which is an important reason why many results are not reproducible and never advance to use in practice [19].

**Fig. 1:**
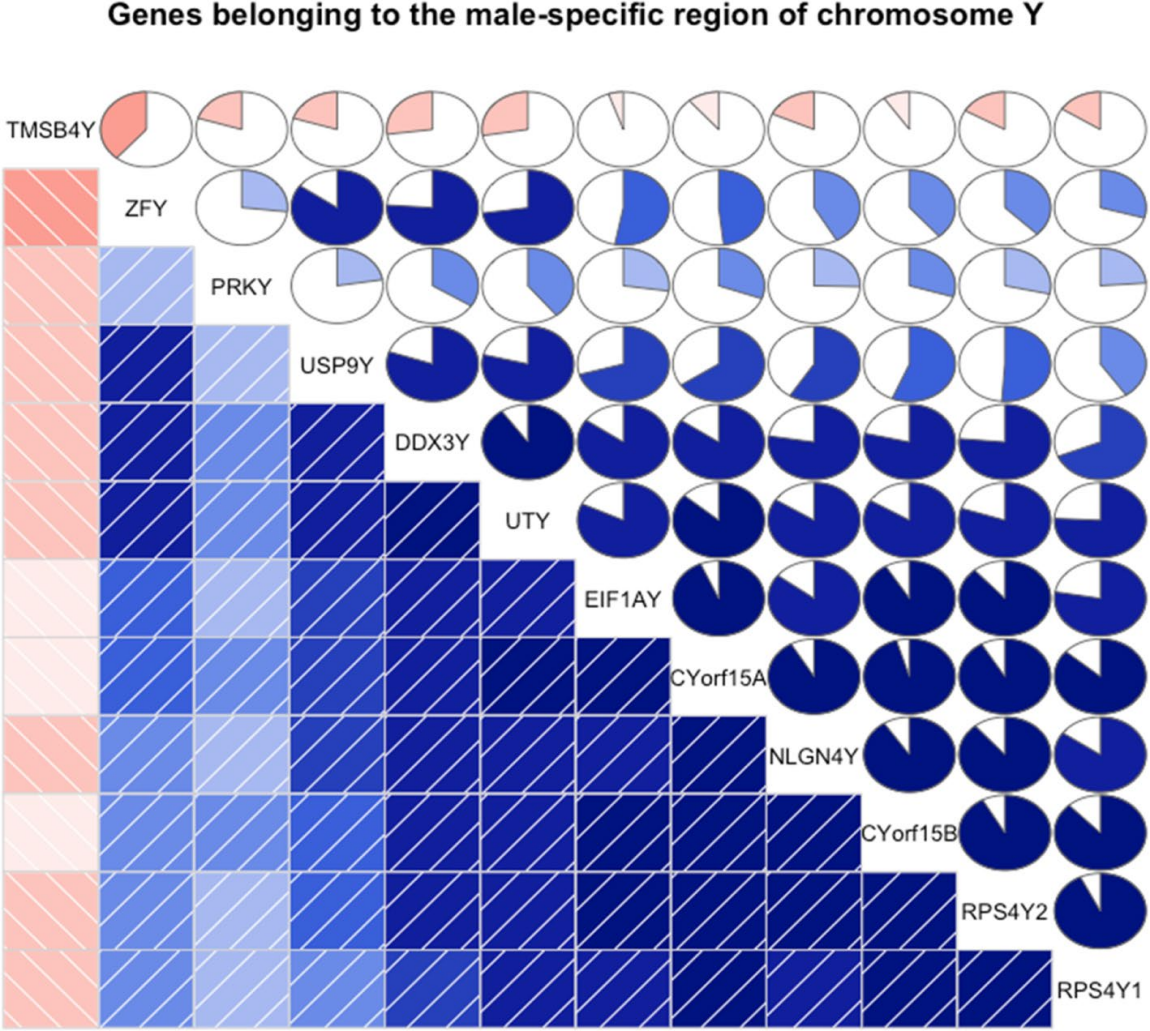
Correlogram of 12 male-specific genes expressed as log-counts-per-million from 69 lymphoblastoid cells derived from male (29) and female (40) Yoruba individuals. Variables (genes) are reordered to emphasize the similarity among of their relations. Lower triangle: correlations shown by color and intensity of shading; upper triangle: by circle filled proportionally to the correlation strength. Given the symmetrical nature of a correlogram, often different representations are used for the lower and the upper triangles. Source for the data [27]

It is important to distinguish technical from biological replicates. Technical replication refers to repeating the measurement process on the same subject. It should not be confused with sample size. Technical replicates are useful for evaluating the variability in the measurement process, which may be comprised of multiple steps each potentially contributing to the total error in the measurement [20] (Fig. 1) described the many steps in gene expression microarray analysis of mouse brains. Technical replication could theoretically be carried out at any of those steps. Sometimes measurements are repeated using an alternative non-high-throughput measurement technique (e.g., RT-PCR assay to measure expression or Sanger sequencing of a specific gene) as a form of measurement validation, but this must not be confused with other forms of validation such as clinical validation of a prediction model (see section “PRED2: Assess performance and validate prediction models”). In presence of budget constraints, if the goal is to compare different biological conditions, it is advisable to invest in biological replicates. When biological samples are inexpensive compared to the cost of the measurement process, pooling is sometimes recommended as a way to reduce costs by making fewer total measurements [21]. However, caution is advised, as assumptions may be required about assay limits of detection or the correspondence between physical pooling and additivity of measurements [22]. The context of any technical replication must be carefully described along with any methods of summarizing over replicates in order to interpret results appropriately.

Design of a study should ideally be placed in the context of an overarching analysis plan. Each individual study should be designed to produce results of sufficient reliability that its results will inform next steps in the research project.

## Methods

### Structure of the paper

This paper is organized with respect to subtopics that are most relevant for the analysis of HDD, particularly motivated by typical aims of biomedical studies but also applicable more generally. These subtopics are initial data analysis (IDA and Preprocessing, section “IDA: Initial data analysis and preprocessing”), exploratory data analysis (EDA, section “EDA: Exploratory data analysis”), multiple testing (section “TEST: Identification of informative variables and multiple testing”), and prediction (section “PRED: Prediction”). For each subtopic, we discuss a list of main analytical goals. For each goal, basic explanations, at a minimally technical level, are provided for some commonly used analysis methods. Situations are identified where performance of some traditional, possibly more familiar, statistical methods might break down in the HDD setting or might not be possible to apply at all when p is larger than n. Strengths and limitations of competing approaches are discussed, and some of the gaps in the availability of adequate analytic tools are noted when relevant. Many key references are provided. It should be noted that throughout this paper we are concerned almost exclusively with cross-sectional or independent observations rather than longitudinal observations.

Topics in the paper are organized into sections according to the structure summarized in Table 1, followed by a discussion of the importance of good reporting to improve transparency and reproducible research in the “Discussion” section and a summarizing discussion in the “Conclusions” section.

**Table 1:** Overview of the structure of the paper, as a list of the sections with corresponding analytical goals and common approaches

Section	Analytical goals	Common approaches	Examples
**IDA**	**Initial data analysis and preprocessing**		
IDA1	Identify inconsistent, suspicious or unexpected values	Visual inspection of univariate and multivariate distributions	Histograms, boxplots, scatterplots, correlograms, heatmaps
IDA2	Describe distributions of variables, and identify missing values and systematic effects due to data acquisition	Descriptive statistics, tabulation, analysis of control values, graphical displays	Measures for location and scale, bivariate measures, RLE plots, MA plots, calibration curve, PCA, Biplot
IDA3	Preprocess the data	Normalization, batch correction	Background correction, baseline correction, centering and scaling, quantile normalization, ComBat, SVA
IDA4	Simplify data and refine/update analysis plan if required	Recoding, variable filtering and exclusion of uninformative variables, construction of new variables, removal of variables or observations due to missing values, imputation	Collapsing categories, variable filtering, discretizing continuous variables, multiple imputation
**EDA**	**Exploratory data analysis**		
EDA1	Identify interesting data characteristics	Graphical displays, descriptive univariate and multivariate statistics	PCA, Biplot, multidimensional scaling, t-SNE, UMAP, neural networks
EDA2	Gain insight into the data structure	Cluster analysis, prototypical samples	Hierarchical clustering, k-means, PAM, scree plot, silhouette values
**TEST**	**Identification of informative variables and multiple testing**		
TEST1	Identify variables informative for an outcome	Test statistics, modelling approaches	*t*-test, permutation test, limma, edgeR, DESeq2
TEST2	Perform multiple testing	Multiple tests, control for false discoveries	Bonferroni correction, Holm’s procedure, multivariate permutation tests, Benjamini-Hochberg (BH), *q*-values
TEST3	Identify informative groups of variables	Tests for groups of variables	Gene set enrichment analysis, over-representation analysis, global test, topGO
**PRED**	**Prediction**		
PRED1	Construct prediction models	Variable transformations, variable selection, dimension reduction, statistical modelling, algorithms, integrating multiple sources of information	Log-transform, standardization, superPC, ridge regression, lasso regression, elastic net, boosting, SVM, trees, random forest, neural networks, deep learning
PRED2	Assess performance and validate prediction models	Choice of performance measures, internal and external validation, identification of influential points	MSE, MAE, ROC curves, AUC, misclassification rate, Brier score, calibration plots, deviance, subsampling, cross-validation, bootstrap, use of external datasets

## Results

### IDA: Initial data analysis and preprocessing

Initial data analysis (IDA) is an important first step in every data analysis and can be particularly challenging in HDD settings. IDA is a term for all steps of data inspection and screening after the analysis plan and data collection have been finished but before the statistical analyses are performed [23, 24]. It focuses on understanding the context in which the data were collected, on data cleaning (see section “IDA1: Identify inconsistent, suspicious or unexpected values”), and on data screening (see section “IDA2: Describe distributions of variables, and identify missing values and systematic effects due to data acquisition”). Data cleaning refers to identifying and possibly correcting errors. Data screening includes reviewing the characteristics of the data that could affect the subsequent analysis plan, for example, describing distributions of variables, by checking assumptions required for model fitting and hypothesis testing, describing missing values, and identifying the need for adjustments of systematic effects due to data collection. Systematic effects may include batch effects that are caused, e.g., by different technologies used for collecting the data or even by different technicians performing laboratory experiments, see section “IDA3.2: Batch correction” for details. Further, initial steps may include simplification of data, e.g., by excluding or collapsing variables, if deemed appropriate. Insights about the data gained from these screening steps might lead to refinement or updating of an analysis plan to ensure that the data are consistent with any assumptions or requirements of the proposed analysis strategies (see section “IDA4: Simplify data and refine/update analysis plan if required”). However, IDA should always be conducted independently of the analysis needed to address the research questions, in order to avoid biasing conclusions.

The term “data preprocessing” is often used in biomedical research involving analysis of HDD, especially in the omics field, to denote certain initial data cleaning and screening steps falling within the more general category of “initial data analysis.” Data preprocessing refers to the process of transforming “raw” data, obtained directly from measurement instrument, into quantifications that are suitable for the subsequent statistical analysis. This includes detection and handling of incomplete, incorrect or inaccurate values, application of normalization methods that aim to remove systematic biases (e.g., assay batch effects), and transformations of variables [25].

A first step of the data cleaning and screening process is often to standardize the names or terms of variables and observations, especially for omics data compiled using different technologies. This type of standardization helps facilitate other, more complex downstream analyses and interpretation of results, as well as better online dissemination and archiving of data.

The IDA material is organized for ease of discussion, but the IDA process is typically iterative. Preprocessing is discussed in section “IDA3: Preprocessing the data,” but after preprocessing one may need to go back to the data cleaning and screening steps described in sections “IDA1: Identify inconsistent, suspicious or unexpected values” and “IDA2: Describe distributions of variables, and identify missing values and systematic effects due to data acquisition.” Note also that some model-based methods used for the identification of informative variables incorporate normalization into the data analysis model (see section “TEST: Identification of informative variables and multiple testing”).

#### IDA1: Identify inconsistent, suspicious or unexpected values

Identification and handling of incomplete, incorrect, or inaccurate values is logically a first step in IDA. Attention is directed toward distinguishing aberrant values that clearly originate from the data collection or generation process from those that might reflect true biological variability. Both visual and analytical inspections of the data are used for the detection of such values.

##### IDA1.1: Visual inspection of univariate and multivariate distributions

Graphical displays are helpful to both understand the structure of the data and detect potential anomalies. For HDD, it is rarely feasible to conduct a detailed examination of the distribution of every variable individually. Visual displays might be constructed only after variables of interest have been identified, for example because a gene is differentially expressed between two experimental conditions or because a particular variable is identified to have an unusual distribution by calculation of summary statistics or has an outlier. A practical alternative is to first calculate scores (summary statistics) for each variable or pair of variables, and then select both typical and interesting atypical variables, with respect to distributions of the scores, for more detailed inspection of their univariate or bivariate distributions. Types of scores to be used in these analyses should include those that capture specific features of the distributions, including measures of location, dispersion, skewness, kurtosis for univariate distributions, linear relationships for bivariate distributions, and metrics to detect outliers or influential values (Table 2).

**Table 2:** Methods for visual inspection of univariate and multivariate distributions: Histograms, boxplots, scatterplots, correlograms, heatmaps

**Fig. 2:**
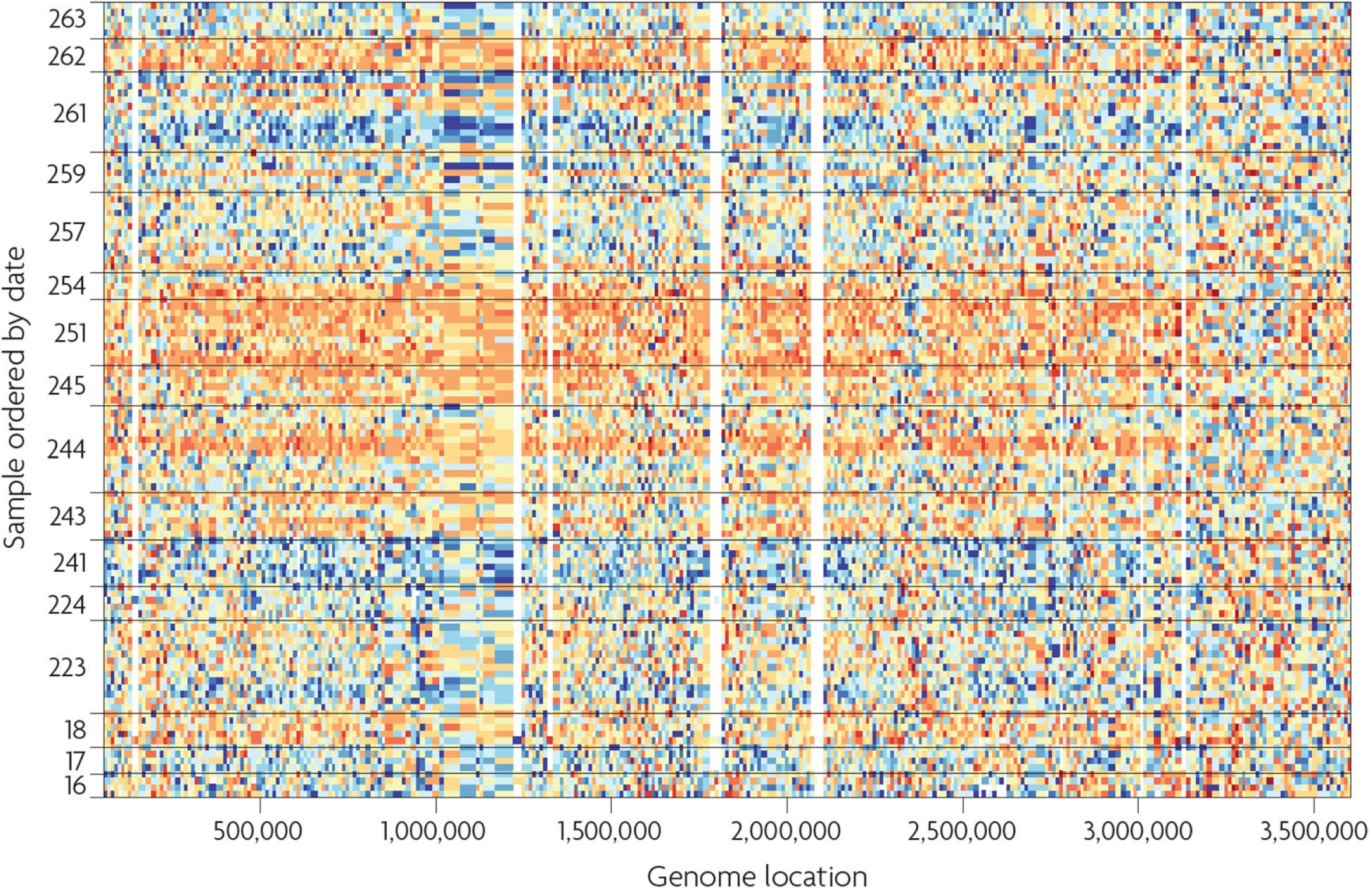
Example for a heatmap, which is a data representation in the form of a map in which data values are color coded. Here, sequencing data from the 1000 genomes project [30] are visualized. Rows correspond to samples and are ordered by processing date, and columns represent genome location of the corresponding sequence. One can see that for the dates 243–254, orange color indicating high values is overrepresented, compared to blue color indicating low values. This demonstrates that so-called batch effects are present, i.e. systematic biases in the data, which are discussed in detail in section “IDA3.2: Batch correction.” Source for the data: [29]

**Histograms**
Histograms divide the range of values into intervals and then count how many values fall into each interval. They can be useful to visualize the shape of the data distribution and identify outlying points. Sometimes use of a transformation before plotting will improve visualization by providing better resolution of densely packed values and drawing more extreme values closer to the main body of the distribution
**Boxplots**
A boxplot (also called box-and-whisker-plot) is a graphical display that gives a quick impression of location and spread of data values and thus makes the comparison between variables simpler. A central box indicates the values that include the central 50% of the data (interquartile range), the median is indicated with a line within the box, and the lines extending vertically from the box (whiskers) indicate the area of all values that are not further than 1.5 times the interquartile range from the edges of the box. In addition, a commonly used option is to plot points individually that are outside the main area indicated by the whiskers. When boxplots are used to display variables with many values (like the expression values of all genes within an experiment), it is expected that many values fall in this category and plotting them individually can create the impression of many extreme values
**Scatterplots**
Scatterplots display one variable plotted against another, with each axis corresponding to one of the two variables. Both variables may be observed (e.g., expression of one gene against expression of a different gene), or one of the two variables could be a factor such as time, order of entry into study, or order in which a measurement such as an assay was conducted. Plotted points may represent the values of two variables for each of the study subjects, or each point could represent one of many different variables measured on an individual subject. For HDD, plots in which each point represents a different variable may contain an extremely large number of points making them hard to interpret due to many overlapping plotting symbols. Strategies such as use of semi-transparent colors for the plotted points or density plots, where regions with more observations appear darker in the plot, may be necessary. Another strategy is to randomly sample points to create a subset that provides a less dense plot
**Correlograms**
A correlogram (or corrgram) is a graphical representation of the correlation matrix [26]. It is a visual display for depicting patterns of relations among variables directly from the correlation matrix. In a correlogram, the values are rendered to depict sign and magnitude. Further, variables can be reordered such that similar variables are positioned adjacently, in order to facilitate the perception of the relations. Since correlograms visualize correlation matrices, they are only useful for LDD, i.e., if the number of variables is not too large. Of course, the correlations themselves can be computed from high-dimensional vectors. Figure 1 [27] shows an example of a correlogram
**Heatmaps**
A common two-dimensional visualization method is a heatmap [28] where the individual values contained in a data matrix are represented as colors in boxes of a rectangular grid. Sometimes raw data values are centered or scaled within rows or columns prior to display, which can be particularly helpful when rows or columns represent variables having different ranges or measurement scales. Clear description of any such centering and/or scaling is essential for proper interpretation of the figure. Choice of color-palette and ordering of rows and columns can both heavily influence the information conveyed by the graphical display. Complementary colors (e.g., red and green, blue and orange) can be used to emphasize two sides of a centered scale. Examples include many published heatmaps for gene expression microarray data in which shades of red might represent degrees of overexpression (relative to median or mean) of a gene and shades of green could represent underexpression. Another consideration for a heatmap display is the ordering of the rows and columns. Sometimes there is an ordering of the observations based on experimental design, for example, samples collected in a time course experiment are represented as ordered columns in the heatmap. As a quality check, it can be useful to order columns by sequence in which samples were assayed. Unexpected trends may indicate assay drift or batch effects. If rows correspond to factors such as gene transcript or protein levels, it can be illuminating to order them according to similarity of pattern across observations. Various clustering methods can be applied to construct orderings of observations or variables. These orderings might be illustrated by dendrograms, which can be displayed along axes of the heatmap to depict the distance structure (see section “EDA2.1: Cluster analysis” for discussion of clustering methods). Figure 2 [29] shows an example of a heatmap

#### IDA2: Describe distributions of variables, and identify missing values and systematic effects due to data acquisition

##### IDA2.1: Descriptive statistics

For understanding the structure of data, often univariate measures for location and scale of the variables are informative. In the HDD setting, graphical display is often helpful to scan these measures across the large number of variables, both for detecting anomalies in the data and for a general exploration of variable distributions and their consistency with assumptions required for certain analysis methods. An example of the use of boxplots and of smooth histograms for exploratory purposes can be found in [31].

Standardization of data values is often performed prior to data analyses. Typically, this refers to normalization with respect to scale and location (e.g., subtract mean or median and divide by standard deviation). This can be helpful to give variables similar weight, especially if they are measured on different scales. However, standardization removes information about absolute magnitude of effects, so it should not be used when the actual magnitude of differences is of interest (e.g., differences in mean expression values between two groups). Another caution is that HDD will typically contain a certain number of variables that are uninformative because they do not vary much across observations, with variability essentially reflecting noise in the data. Standardization of such variables can exaggerate the noise to give these variables undue influence in analyses that is on par with that of truly informative variables. It is often preferred to drop such uninformative variables at the start of analyses (Table 3).

**Table 3:** Methods for descriptive statistics: Measures for location and scale, bivariate measures, RLE plots, MA plots

**Fig. 3:**
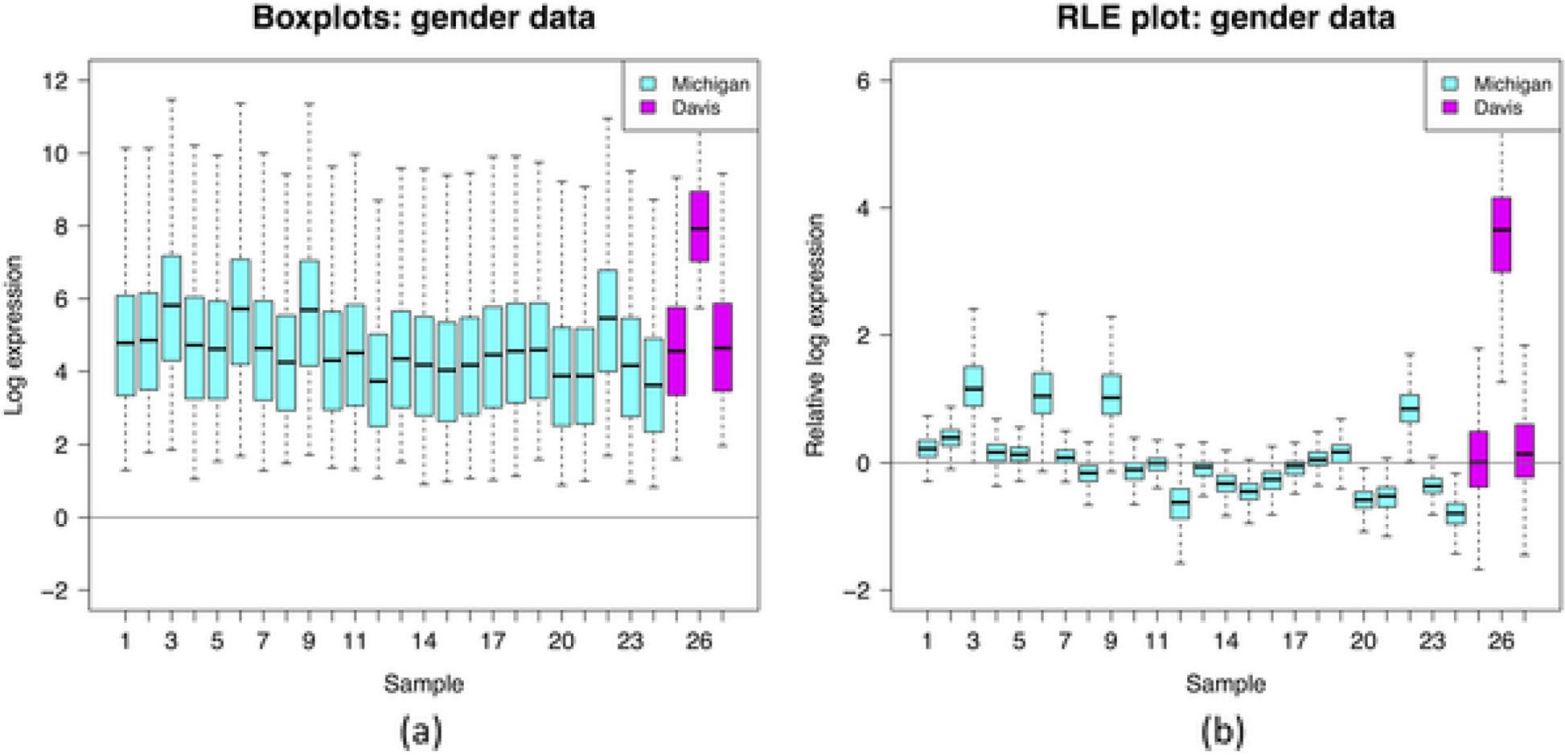
Visualization of the insights obtained from an RLE plot, representing (**a**) log gene expression distributions for 27 samples (without performing quantile normalization) and (**b**) relative log gene expression distributions for the same 27 samples. The RLE plot allows to highlight the unwanted variation due to the between-batch variation (cyan versus magenta boxplots) as well as the within-batch variation as suggested by both the difference in location (median further from 0) and spread (higher IQR) of the boxplots. This interpretation is under the often-plausible assumption that expression levels of most genes are unaffected by the biological factors of interest. Source: [32]

**Fig. 4:**
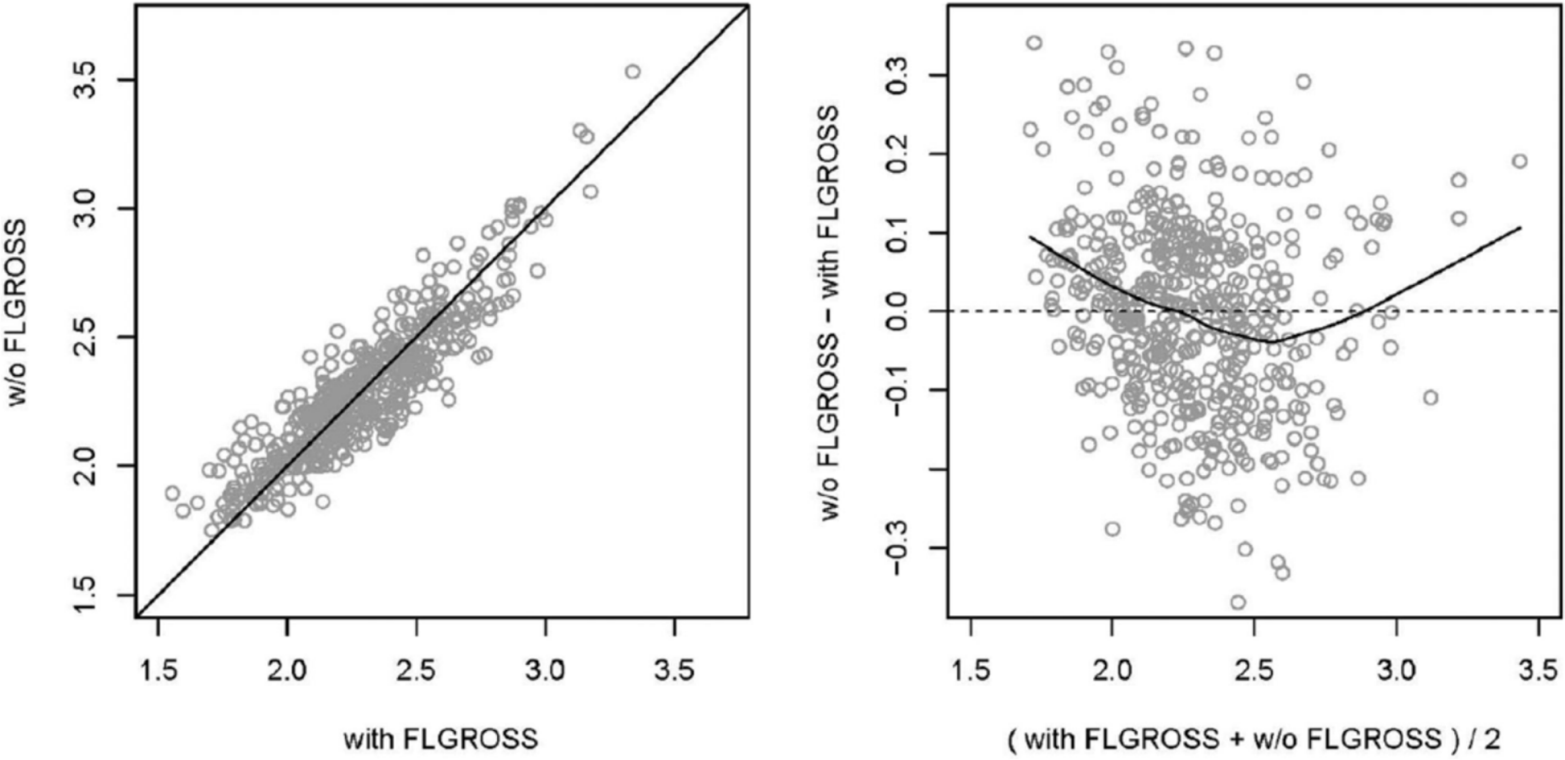
Comparison of a scatterplot (left) and a Bland–Altman plot (right, also MA plot for omics data) of the same data. In this example, the predicted values of two regression models (including and excluding a variable called FLGROSS) are compared. The scatterplot shows similar values for most observations, with points close to the diagonal. The Bland–Altman plot, with differences on the *y*-axis (on log-scale for MA plots on omics data typically log-ratios), better visualizes the dependence on the average value of the predictions (typically average log intensity for MA plots). The smoothing line in the example Bland–Altman plot indicates the shape of dependence of the differences on the average values. Source: [35]

**Measures for location and scale**
As measure of location, the mean is standard for continuous data, the median is robust regarding extreme values, and the mode is often used for categorical data. Such measures can be extended to higher dimensions by calculating them component-wise, i.e., for every variable separately, and then collecting the values into a vector
As measure of scale, the standard deviation for continuous data and the median absolute deviation to the median (MAD) as a robust counterpart are often used. The coefficient of variation scales the standard deviation by dividing by the mean and is helpful for comparing variables that are measured on different scales
**Bivariate measures**
Bivariate descriptive statistics are based on pairs of variables, often the correlation coefficient is used to quantify the relationship between two variables. The classical Pearson correlation coefficient captures only linear relationships, whereas Spearman’s rank-based correlation coefficient may more effectively capture strong non-linear, but monotonic, relationships
**RLE plots**
Relative log expression (RLE) plots [32] can be used for visualizing and detecting unwanted variation in HDD. They were developed for gene expression microarray data, but are now very popular especially for the analysis of single-cell expression data. For each variable (e.g., expression of a particular gene), first, its median value across all observations is calculated. Then, the median is subtracted from all values of the corresponding variable. Finally, for each observation, a boxplot is generated of all deviations across the variables. Comparing the boxplots, if one of them looks different with respect to location or spread, it may indicate a problem with the data from that observation. RLE plots are particularly useful for assessing the effects of normalization methods that are applied for removing unwanted variation, which might be due to, e.g., batch effects, see also section “IDA3.2: Batch correction.” An example RLE plot is presented in Fig. 3 [32]
**MA plots (Bland–Altman plots)**
A natural way to assess concordance between measurements that are supposed to be replicates is to construct a simple scatterplot and look for distance from the 45-degree line. However, a preferred approach is to construct a Bland–Altman plot [33] instead of a scatterplot. In the omics literature, this plot is often referred to as an MA plot [34]. The horizontal (“*x*”) axis of a Bland–Altman plot is the mean of the paired measurements, and the vertical (“*y*”) axis is the difference, often after measurements have been log transformed. The advantage of this plot compared to a traditional scatterplot is that it allows better visualization of differences against a reference horizontal line at height zero and improved ability to detect changes in variability (spread) of those differences moving along the *x*-axis (see section “IDA4: Simplify data and refine/update analysis plan if required”). An example of a Bland–Altman Plot is presented in Fig. 4 [35]

##### IDA2.2: Tabulation of missing data

Missing values are ubiquitous in real-world data and may have major implications for choice of analysis methods and interpretation of results [36]. In fact, most multivariable and multivariate analyses methods have as their default requirement that values of all variables are available for all subjects, i.e., all observations are “complete.” An important early step in any analysis is tabulation of the missing values, i.e., the identification of the number of missing values per subject and per variable, respectively, to provide an overview of the missing data structure. In multi-omics integrative studies, high-dimensional data from different data types are collected for the same subjects. In such studies, small sample size caused by experimental and financial constraints, which can also vary between data types, can be the reason for missing data, the absence of which has to be taken into account in the subsequent statistical analysis.

##### IDA2.3: Analysis of control values

Laboratory assay measurements can be affected by technical artifacts related to factors such as reagent lots, equipment drift, or environmental conditions. Sometimes these artifacts can be detected, and potentially adjusted for, through use of control and reference standard samples, which have expected distributions of measurements. For single-analyte assays, a calibration process is typically performed to adjust raw measurements and produce final reported values (Table 4).

**Table 4:** Method for analysis of control values: Calibration curve

**Fig. 5:**
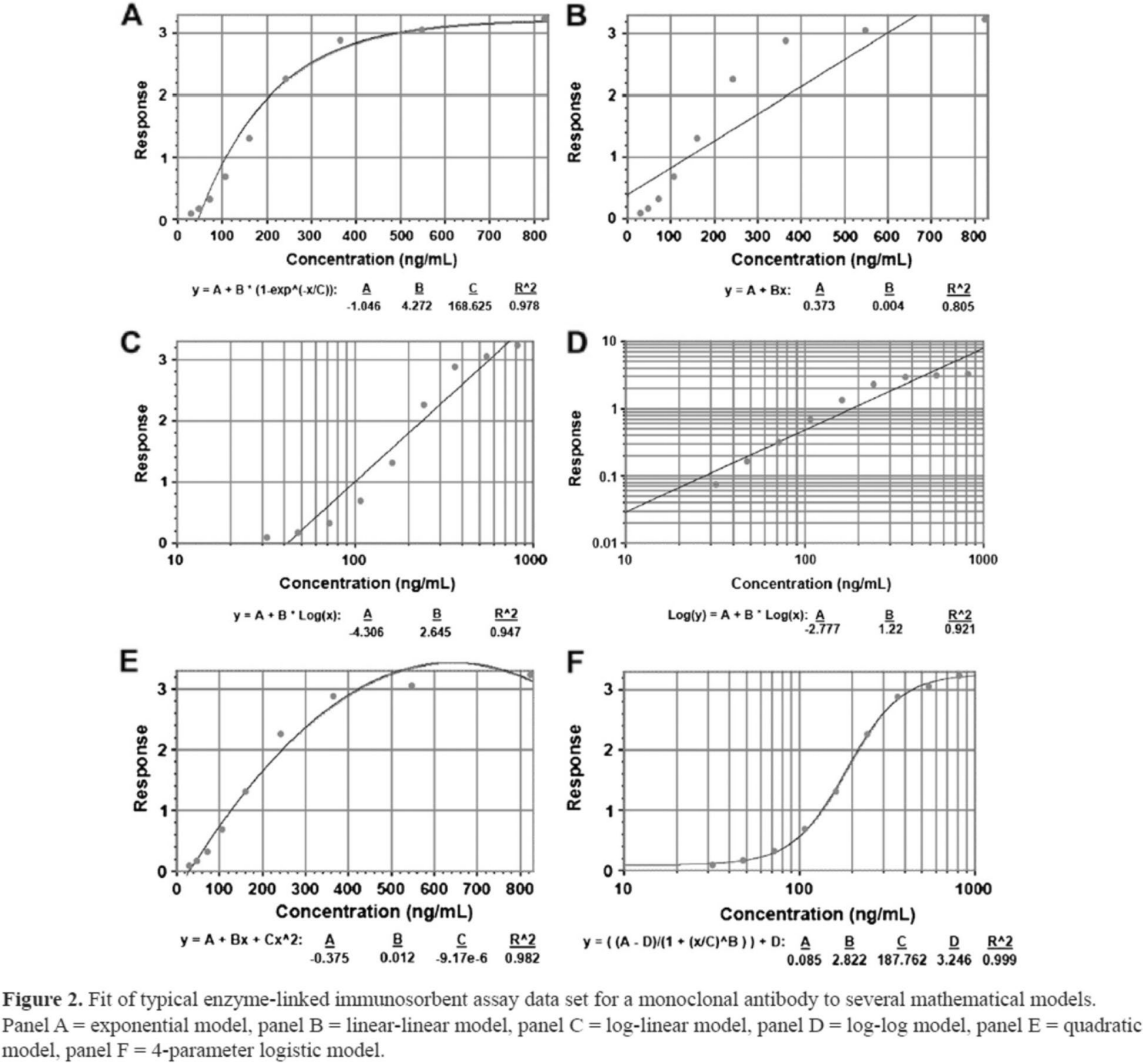
Visualization of calibration curves, representing the relationship between values of an analyte measured on a set of samples by some experimental assay (*y*-axis) and values obtained for those samples from some reference assay that is considered to represent truth and to be measured with negligible error (*x*-axis). The curve may be inverted to correct values obtained from the experimental assay to bring them closer to values of the analyte that would have been expected from the reference assay. Source: [37]

**Calibration curve**
A typical calibration process for a single-analyte assay might involve running a series of reference standard samples with known values of the target analyte followed by construction of a calibration curve. This curve can then be inverted to produce a mathematical correction that is applied to the raw measured values from the test samples. A multiplicative factor applied to all raw assays values is a simple example of a correction
In the setting of HDD such as omics data, it would be infeasible to construct a separate calibration curve for every analyte measured by the assay. Instead, calibration approaches used for omics assays typically rely on corrections derived either from a small subset of the analytes measured by the assay platform or on assumptions about the global distribution of the measured values across all analytes measured. An example of the subset approach in the context of gene expression arrays is the calculation of a mean over a small set of so-called “housekeeper genes”, whose expression levels are expected to be roughly constant across all samples being analyzed. This mean is compared to a specified reference value to generate a multiplicative factor specific to each sample, which is then applied globally across the expression data for all genes measured for the sample. Figure 5 [37] shows several examples of calibration curves

##### IDA2.4: Graphical displays

Systematic artifact effects arising from data acquisition processes can often be detected with graphical displays that visualize the data in a comprehensive manner. A widely used graphical representation for multivariate data is a principal components plot, which is also useful in exploratory data analysis, as described in section “EDA: Exploratory data analysis” (Table 5).

**Table 5:** Methods for graphical displays: Principal component analysis (PCA), Biplot

**Fig. 6:**
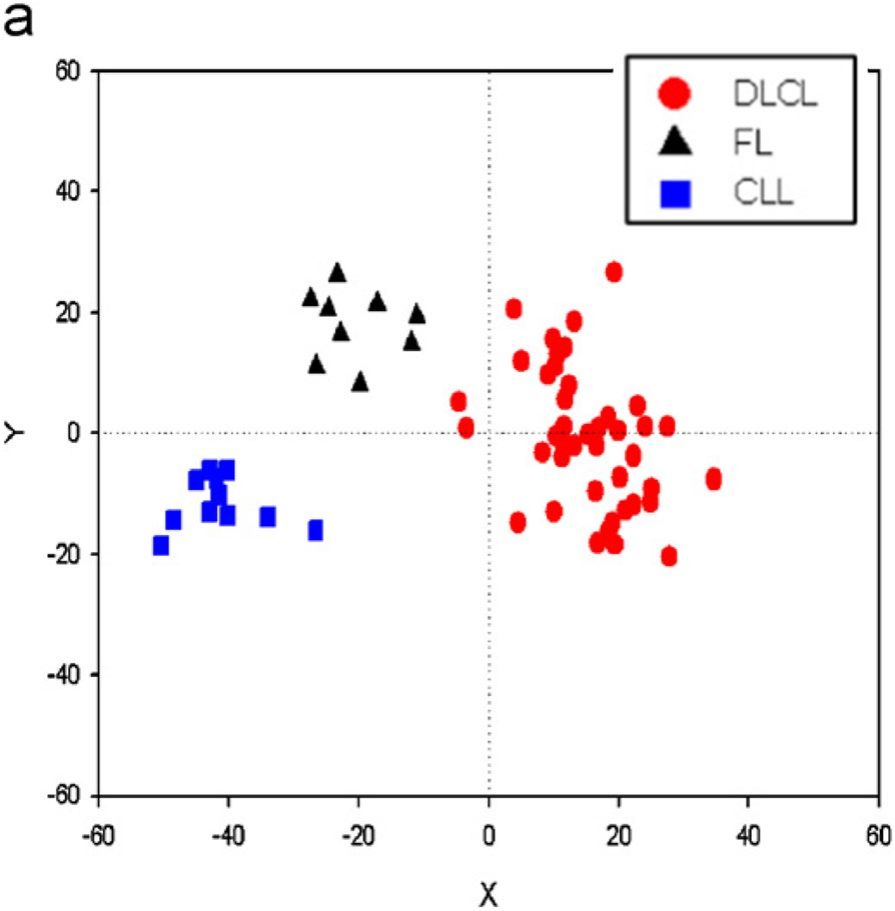
Principal component analysis plot depicting 62 lymphoma samples represented by their first and second principal component calculated from gene expression profiles comprising expression levels of 4026 genes on each lymphoma sample. The samples have been annotated in the plot according to pathologic subtype: 11 B-cell chronic lymphocytic leukemia (B-CLL; blue squares), 9 follicular lymphoma (FL; black triangles), and 42 diffuse large B-cell lymphoma (DLCL; red dots). Source: [39]

**Fig. 7:**
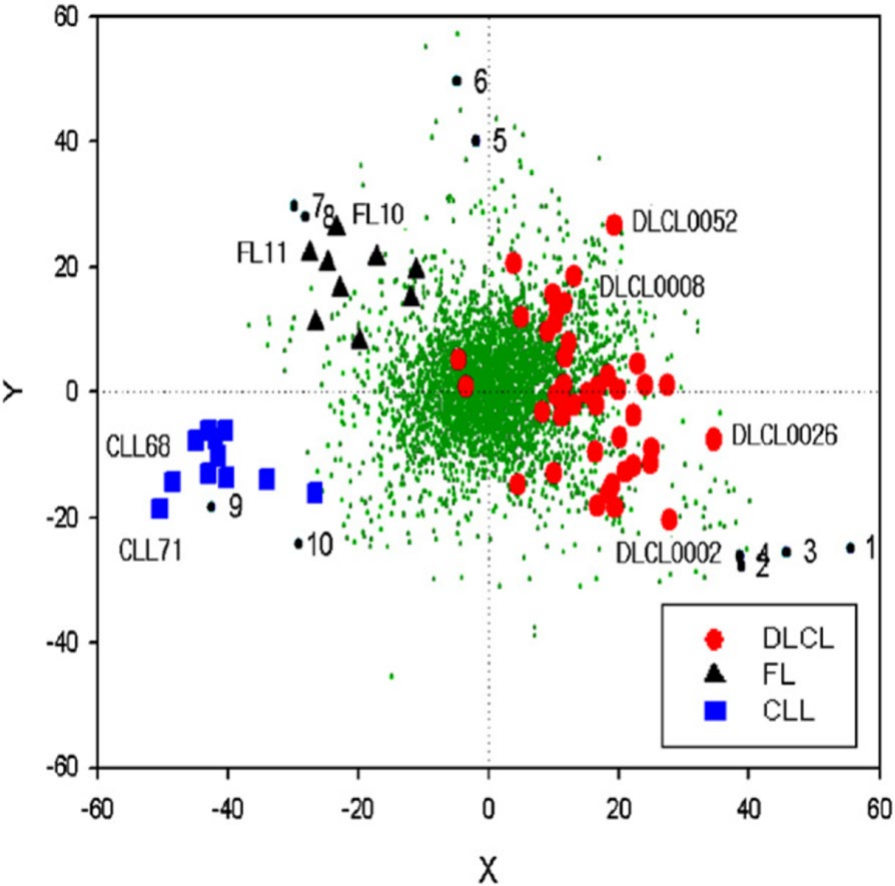
Biplot constructed by superimposing a PCA plot of 62 lymphoma samples (see Fig. 6) onto a PCA plot of genes where first and second principal component for the genes are calculated from gene expression profiles comprising expression levels of the 62 samples for each gene. Genes are represented in the plot as small green dots. Genes representing the three classes well are indicated by numbers. Source: [39]

**Principal component analysis (PCA)**
The basic idea of PCA [38] is to transform the (possibly correlated) variables into a set of linearly uncorrelated variables as follows. The first variable (first principal component) is constructed to capture as much of the total multidimensional variability in the data as possible, the second variable is uncorrelated with the first and maximizes capture of the residual variability (i.e., variability not already captured by the first principal component), and so on. Each principal component is a linear combination of the original variables. The result is a set of uncorrelated variables of decreasing importance, in the sense that the variables are ranked from the most informative (the first principal component, i.e., the one with the highest variance) to the least informative (i.e., the one with the lowest variance). The positions of each observation in the new coordinate system of principal components are called scores, and the loadings indicate how strongly the variables contribute to each PC. A major portion of the total variation in the data is often captured by the first few principal components alone, which are the only ones retained for the further analysis. Use of principal components greatly reduces the dimension of the data typically without losing much information (with respect to variability in the data). In the context of IDA, often the first two principal components are plotted to inspect for peculiarities in the data. Figure 6 [39] shows a PCA plot constructed from high-dimensional gene expression profiles generated from analysis of lymphoma specimens
**Biplot**
Biplots, introduced by Gabriel [40], are designed to show PCs’ contributions with regard to both observations and variables. In a biplot, both the principal component scores and loadings are plotted together. The most common biplot is a two- or three-dimensional representation, where any two (or three) PCs of interest are used as the axes. Since often most of the variation in the data is explained by the first few PCs, it usually suffices to concentrate on plotting those. The biplot allows identifying samples that are “different” from the majority of samples, and at the same time, it illustrates nicely where these differences occur, i.e., for which variables the samples show different values. Figure 7 [39] shows a biplot for the same data used for the PCA plot

#### IDA3: Preprocessing the data

Data generated by omics assay technologies typically require preprocessing by specially tailored methods that are based on understanding of the sophisticated instrumentation and scientific underpinnings of the technologies. Omics data are some of the most frequently encountered HDD in biomedical settings and are the focus in this paper. However, similar challenges exist with other types of HDD in biomedical research. Notably, high-dimensional imaging data are becoming commonplace, with examples including those generated by digital radiography, PET scans, and magnetic resonance imaging. In the following, we explain the main principles of data preprocessing using omics data examples.

Omics technologies are highly sensitive to experimental conditions and can exhibit systematic technical effects due to time, place, equipment, environmental conditions, reagent lots, operators, etc. In general, the first step of preprocessing aims to obtain an “analyzable” signal from the “raw” measurements. Subsequently, the signal is separated from possible systematic technical effects. The corrected signal may then be transformed to fulfill certain distributional properties, e.g., approximating a normal distribution. Note that sometimes the transformation may be applied before correcting the signal.

Preprocessing aimed at removal of systematic effects is often conducted as a separate step, as part of the IDA process, before the statistical analysis for answering the research question is undertaken. If the data have already been corrected for systematic effects and retain only the signals of interest (e.g., treatment effects), then the preprocessed (“normalized”) measurements for the biological samples can be analyzed using statistical methods that are easily accessible to researchers. However, conducting normalization as a separate step has important disadvantages. For instance, the normalized values are estimates and often carry with themselves some uncertainty, which should be taken into account in the analysis of the normalized data. However, this complicates the statistical analysis.

If inferential analysis is of interest, e.g., when comparing groups of samples to assess for biological differences, then a preferred approach is to consider normalization as part of a comprehensive statistical analysis model. The model is then used both to remove systematic technical differences and to quantify biological effects of interest (e.g., treatment effects). In that case, the uncertainty related to the normalization part of the analysis is naturally included in the estimates of uncertainty (standard errors) of the quantities of biological interest.

##### IDA3.1: Background subtraction and normalization

Omics data are prone to perturbations due to systematic effects induced by the measurement technology, also referred to as the assay *platform*. Many of these effects are unique to the assay platform, but there are some commonalities. A biological sample may have its gene expression profile measured using a single microarray or gene chip or its protein profile measured using a mass spectrometry system. The set of experimental conditions that gives rise to profiles such as these will be referred to here as an experimental *run*. However, even for the same sample, measurements obtained in different runs may differ due to factors such as different amounts of biological material input to the measurement system, settings on the instrumentation, environmental conditions in the laboratory, and so forth. These “between-run” differences may confound the “between sample” comparisons of scientific interest. Thus, these nuisance run effects should be removed to allow valid comparisons among data obtained in different runs. A generic preprocessing step aimed at removing between-run differences is often termed *normalization*. Even before normalization methods are applied, data generated by omics technologies generally require correction to subtract background noise from measurements to reveal their signal components. In Table 6 we introduce some basic methods for background subtraction and normalization.

**Table 6:** Methods for background subtraction and normalization: Background correction, baseline correction, centering and scaling, quantile normalization

**Fig. 8:**
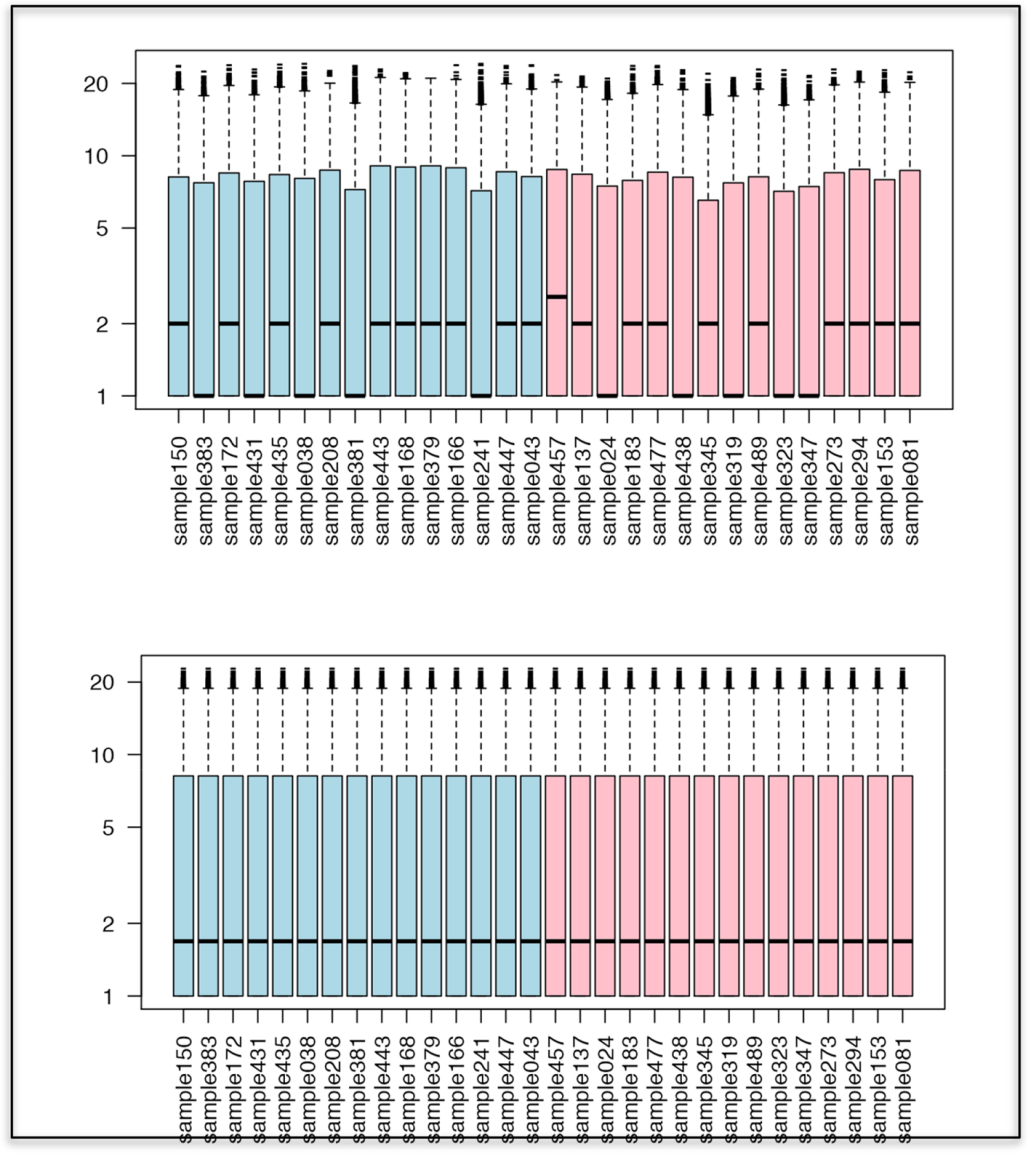
Boxplots representing artificial distributions of values for 30 samples (subjects), before quantile normalization (top) and after quantile normalization (bottom), showing that all distributions are fully aligned with each other after the transformation. Source: [44]

**Background correction**
A classic example of such a step is a background correction applied to data generated from some of the earliest microarrays [41]. In this approach, the signal of interest is obtained by summarizing the pixel intensity values within a designated region or “spot” (e.g., corresponding to location of probe for a particular gene) on a scanned image of a hybridized array. Ideally, pixels for areas outside the spots should have zero intensity, but this is rarely the case because of the fluorescence of the array surface itself. This fluorescence is termed the background. Because background may contaminate the measurement of spot fluorescence, the signal in the spot should be corrected for it by subtracting the fluorescence measured in the background
**Baseline correction**
In proteomic mass spectrometry [42], the counterpart of background correction is “baseline correction.” In mass spectrometry, the mass-to-charge ratio (m/z) of molecules present in a sample is measured. A resulting mass spectrum is an intensity vs. m/z plot representing the distribution of proteins in a sample. In this technology, chemical noise is usually present in the spectrum, which is typically caused by chemical compounds such as solvent or sample contaminants that did not originate from the analyzed biological sample. Chemical noise can cause a systematic upward shift of measured intensity values from the true baseline across a spectrum. The presence of baseline noise poses a problem, as the intensity is used to infer the relative abundance of molecules in the analyzed sample. A baseline shift will distort those relative measures; hence, baseline subtraction is typically applied when preprocessing mass spectrometry data
**Centering and scaling**
Normalization aimed at addressing between-run differences typically involves re-centering or re-scaling data obtained for a particular run by applying a correction factor that captures the difference between the measurements from that run and measurements from some type of average over multiple runs or from a reference run. The correction factor may be obtained by using internal controls or standards. These can be either analytes known to be present in the sample or analytes added to the sample that should, theoretically, yield the same measurements if the same amount of sample material is measured. If the measured values of internal standards differ across runs, then these internal control or standard values can be used for re-centering or re-scaling purposes
An alternative approach is to use a run-based estimate of the constant that is calculated across the many measured variables for an individual sample. Examples include re-centering or re-scaling the measurements by their mean value (as in the total ion current normalization of mass spectrometry data), or by an estimate reflecting the amount of processed biological material (as in library size normalization of next-generation sequencing data)
Data preprocessing terminology can be confusing for high-dimensional omics data. Although centering and scaling are often referred to generically as standardization, here, centering and scaling will refer to adjustment to all values of one observation (across variables). Standardization as meaning centering and scaling of all values of a variable (across observations) is described in section “PRED1.1: Variable transformations.”
**Quantile normalization**
Quantile normalization [43] is a widely used normalization procedure that addresses between-run differences and has been popular for use with omics data. The method assumes that the distribution of measured values across the many analytes measured is roughly similar from sample to sample, with only relatively few analytes accounting for differences in phenotypes (biological or clinical) across samples. Quantiles of the distribution of raw measured values (e.g., across genes) for each sample are adjusted to match a reference distribution, which is obtained either from a reference sample or constructed as some sort of average over a set of samples. Although the numerical quantiles are forced to match, the particular analyte (e.g., pertaining to a certain gene) to which the quantile corresponds can vary from sample to sample, thus capturing the biological differences across samples. Figure 8 [44] shows the effect of quantile normalization

##### IDA3.2: Batch correction

Another example of a systematic effect that is common to many technologies is a “batch effect.” The effect may arise when groups of biological samples (“batches”) have something in common in the way they are processed, e.g., same day or time of day, on same instrument, same operators, but these aspects are different for other groups of samples. Besides these measurement conditions, factors at play prior to measurement can cause batch effects. For example, clinical centers might differ in their standard operating procedures for processing, handling, and storing biospecimens, giving rise to pre-analytic factors that could influence downstream measurements. Patient characteristics, co-morbidities, or concomitant medications could additionally vary by batch, and may give rise to different distributions of measured values that have biological basis. Batch effects are widespread [29]. The challenge for batch correction is removal of nuisance effects such as those due to pre-analytic or technical factors while not inadvertently removing true biological differences. To facilitate appropriate correction, batch information such as dates, instrument, operator, and specimen collection sites should be recorded and patient factors might need to be taken into account in analyses. Above all, it is critical to avoid poor study designs in which important patient characteristics (including outcomes) are confounded with nuisance batch effects, as this could make it impossible to remove nuisance batch effects adequately.

Preprocessing of omics data aimed at removal of the aforementioned artifact effects poses several challenges. For instance, normalization is often data-driven and uses methods based on assumptions about the nature of the biological mechanisms. If those assumptions do not hold, then the methods might not work as intended. An example of a commonly made assumption in experiments involving genome-wide expression data is that most genes are not differentially expressed under the compared conditions. It may be challenging to verify whether such assumptions are correct.

The dependence of systematic effects on the platform raises an important issue for novel technologies, for which sources of measurement variation may not be fully established or understood. Out of convenience, preprocessing approaches developed for one platform have often been applied to other platforms. For example, normalization methods developed for microarrays are also used for proteomic [45] and metabolomic [46] mass spectrometry experiments. This might be reasonable in some settings, but the assumptions required for adequate performance of a normalization method should always be reviewed carefully for appropriateness prior to its application to another technology.

In addition, it is worth noting that preprocessing may need to be tailored to the analysis goals. For instance, it is problematic to remove batch effects when constructing a classification rule. This is because the measurements for a new sample presented for classification will most likely include effects from batches not represented in the data used to construct the classification rule. Consequently, a classification rule should be constructed using data that have not been batch corrected so that robustness to batch effects can be built in (Table 7).

**Table 7:** Methods for batch correction: ComBat, SVA (surrogate variable analysis)

**Fig. 9:**
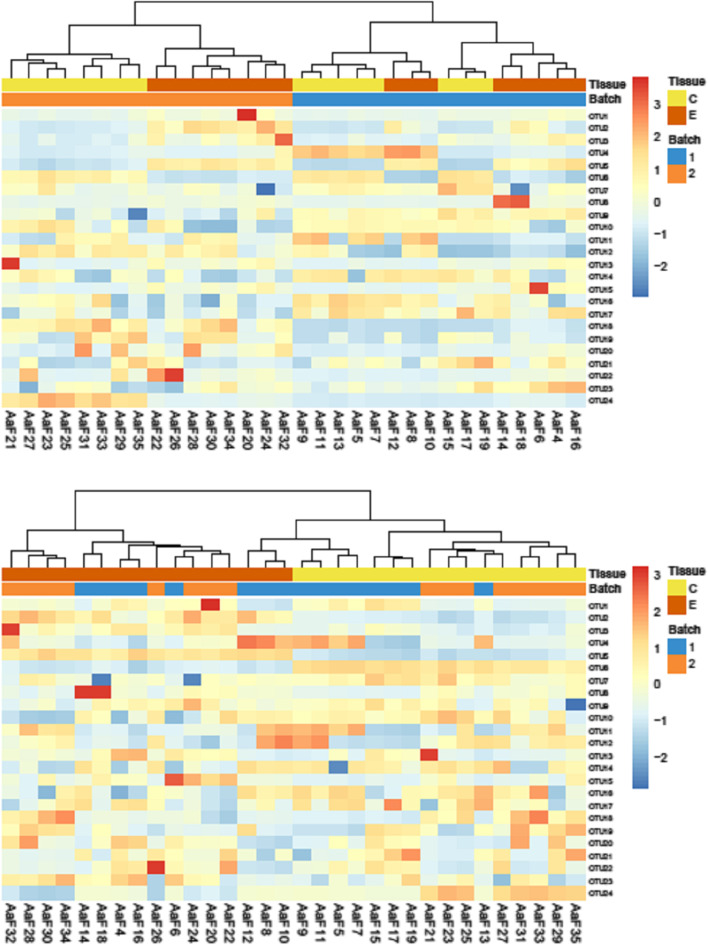
Visualization of the effect of batch correction. Heatmaps of hierarchical clustering of sponge metagenomics data studying two tissues types (C and E) with 2 batches, before and after Combat batch correction. Without batch correction (top figure), the clustering is mainly driven by the batch effect. After correction, the clustering is driven by the tissue type (bottom figure). Source: [49]

**ComBat**
ComBat is a widely used batch correction method that has been shown to have generally good performance [47]. For each gene, this method estimates location and scale parameters for each batch separately. Then the data are transformed using these parameter estimates so that the location and scale parameters are the same across batches. This method is robust to outliers also in small sample sizes and thus especially well-suited for HDD analysis. ComBat-Seq [48] is an extension specifically developed for count data using a negative binomial model, and it is compatible with differential expression algorithms that require counts. Figure 9 [49] shows an example of the effect of ComBat, comparing the results with and without using this batch correction.
**SVA (surrogate variable analysis)**
Variability in measurements may arise from unknown technical sources or biological sources that are not expected or controllable and can affect the accuracy of statistical inference in genome-wide expression experiments. SVA [50] was developed to deal with the unmeasured factors that influence gene expression by introducing spurious signal or confounding biological signal. SVA identifies unobserved factors and construct surrogate variables that can be used as covariates in subsequent analyses to improve the accuracy and reproducibility of the results. SVA was first developed for microarray data and later adapted for sequencing data [51].

#### IDA4: Simplify data and refine/update analysis plan if required

The findings from the IDA steps can have substantial impact on the choice of appropriate analytical methods for subsequent statistical analyses. Therefore, the analysis plan should be refined or updated as necessary and according to the relevant findings from the IDA analysis [23].

##### IDA4.1: Recoding

Recoding primarily refers to transformations of the (original, raw) data, which allow for easier handling for a specific purpose. This is particularly useful in HDD settings, in which simple representation of the information can be challenging and sometimes even impossible due to the large number of variables (Table 8).

**Table 8:** Method for recoding: Collapsing categories

**Collapsing categories**
When a categorical variable has substantial imbalance in its distribution across categories, especially when relatively few observations are assigned to a certain category, it can cause instability in analyses. Models incorporating categorical variables with substantial imbalance can be strongly influenced by them. To avoid the undue influence of a rare category on the analysis, it may be necessary to accept the information loss by collapsing the variable, i.e., merging the rare category with another category that is similar in terms of content but more frequent.

##### IDA4.2: Variable filtering and exclusion of uninformative variables

Variable filtering refers to the exclusion of variables that are considered uninteresting, before the statistical analysis to address the main research question is even started. This practice is widespread in HDD analysis where any steps to reduce the dimensionality and complexity of models at the outset are appreciated. If many irrelevant variables are filtered out, the multiple testing problem (see section “TEST: Identification of informative variables and multiple testing”) is diminished, and the statistical power of subsequent analysis steps can substantially increase. However, as discussed below, caution is required when applying certain filtering strategies that may introduce bias (Table 9).

**Table 9:** Methods for filtering and exclusion of variables: Variable filtering

**Variable filtering**
Variable filtering is typically accomplished by calculation of a score for each variable, followed by exclusion of variables having a score below a threshold from further analyses. Modelling or multiple testing procedures can then be applied only to the resulting variable set. However, in order to preserve the correct error control in multiple testing, it is crucial that the filtering is independent of the test statistics that will be used to analyze the filtered data [52]. This is generally accomplished using “nonspecific” filters, where the filtering does not depend on the outcome data. For example, when comparing groups using two-sample *t*-tests, first removing the variables that exhibit a small difference in the mean values of the classes and then applying the multiple testing corrections to the remaining variables leads to greatly inflated type I errors and overoptimistic multiplicity adjusted *p*-values. In contrast, type I error is correctly controlled if the filter is based on the overall variance or mean of the variables (combined across both groups), filtering out the variables with small overall variability or low overall expression [52–54]. Although computationally helpful, filtering that does not inflate errors also does not necessarily increase statistical power; for example, Bourgon et al. [52] showed an example for Affymetrix gene expression data, where filtering out a large proportion of the genes with low expression actually decreased the number of true discoveries
Variable filtering is implicitly performed also by some methods that can be used in regression modelling. These methods include Lasso, which will be discussed in the context of prediction modelling in section “PRED: Prediction.”

##### IDA4.3: Construction of new variables

Sometimes it is useful to construct new variables as an initial step of data analysis by combining the variables that are available in the dataset in a meaningful way, using expert knowledge. For example, in medical studies investigating factors affecting health, often, overweight status is an important variable to consider in the analysis. Because weight and height must be considered together in assessing whether an individual is overweight, constructed variables like body mass index (BMI) have been used. The importance of fat distribution has also been recognized, and it has motivated the combined measured of waist-hip ratio (WHR). Instead of relying on the ability of statistical methods and algorithms to construct such variables implicitly, e.g., during a statistical modelling process, it is useful to be informed by expert knowledge and to include these constructed variables directly into analyses.

Not all constructed variables are derived using expert knowledge. Some, like principal component scores (see section “IDA2.4: Graphical displays”), are constructed in an unsupervised manner meaning that they are constructed to capture features of the data based only on the explanatory variables without using dependent variables such as outcomes. These constructed variables are sometimes used as explanatory variables when building prediction models (see section “PRED: Prediction”), and they can also be used for exploratory data analysis (see section “EDA: Exploratory data analysis”). As discussed in section “IDA2.4: Graphical displays,” plots of (typically the first two) principal components are often helpful for detecting peculiarities in the data or problems such as batch effects. Some constructed variables are derived using outcomes or other dependent variables. Examples of outcome-informed constructed variables include supervised principal component [55], or partial least squares (PLS) scores (see section “PRED1.3: Dimension reduction” for further discussion). Sometimes new variables are constructed by discretization of continuous variables, but this practice is problematic and should generally be discouraged (Table 10).

**Table 10:** Method for construction of new variables: Discretizing continuous variables

**Discretizing continuous variables**
Discretization of a variable refers to the process of converting or partitioning a continuous variable into a nominal or ordinal categorical variable. Often, the variable is discretized into partitions of equal width (e.g., when constructing a histogram) or of equal frequencies (e.g., quartiles). Alternatively, the categorization may be based on historical context, for example if it is known that age above a certain threshold is a risk factor for a specific outcome. However, categorization introduces several problems and is often criticized in LDD [56, 57], especially for the extreme version with only two groups, called dichotomization. This simplification of the data structure often leads to a considerable loss of power, and the use of a data-driven optimal cutpoint for dichotomization of a variable leads to a serious bias in prediction models including the variable

##### IDA4.4: Removal of variables or observations due to missing values

The simplest approach to deal with missing data is a “complete case analysis.” That is, if a single variable is missing for an observation, the observation is fully excluded from the dataset. Basing analyses on only complete cases at best only leads to loss of statistical power, but at worst can lead to substantially biased analyses. Impact of missing data will depend on how many cases have missing data, how many variables have missing values, how many values are missing, and whether the likelihood of missing values in a variable is related to the value of that variable or other variables. When few observations have missing values for few variables, then the impact on results of subsequent analyses may be limited, but when the number is large, the impact can be substantial.

A typical strategy for dealing with missing data is to exclude variables from the analysis that have a large number of missing values. Obviously, the possible relevance of such variables is neglected. Only when the missingness (the events that lead to a value being missing) is independent of both unobserved and observed values, i.e., the data are missing “completely at random” (MCAR), are the results of the complete case analysis (using likelihood-based methods) unbiased. When missing values depend on the unobserved values themselves (e.g., it is more likely that the measurement of a variable is missing when the value of the biomarker is very high or very low), then the missing values are said to be “missing not at random” (MNAR), and the resulting complete case analysis is biased.

Between the two extreme situations of MCAR and MNAR, there is a third possibility: missing values are called “missing at random” (MAR), when the missingness is independent of the unobserved values after controlling for the other available variables. One way to diagnose whether data are MCAR or MAR is to tabulate a missing value indicator against the values of other variables. As an example, if the value of a biomarker (e.g., gene expression level) is missing with higher frequency in males than in females, but within these strata, the missing values are missing completely at random, then it is likely a situation of MAR and not MCAR.

In HDD settings, when a large number of variables must be considered, complete case analysis may require exclusion of too many observations. To avoid this, common approaches involve first removing variables for which more than a specified percentage (e.g., 5 or 10%) of observations are missing and then removing observations for which more than a specified percentage (e.g., 2%) of variables have missing values. For studies with more complex designs, additional considerations may apply. For example, it is common in case–control studies to remove variables for which there is larger imbalance (e.g., more than 5 or 10% difference) in the percentage of missing values between cases and controls.

##### IDA4.5: Imputation

For MAR situations, methods more sophisticated than complete case analyses or dropping variables are recommended to use the information from all observations in the study and obtain less biased results. An example method is multiple imputation, which is described below. Although imputation is a useful strategy, it should be understood that no single approach for dealing with missing data is fully satisfactory. Thus, the best approach is to carefully select variables that are both informative and feasible to collect when designing studies and then work diligently to collect those data as completely as possible in order to minimize the amount of missing information. In the context of LDD, a framework for the treatment and reporting of missing data was proposed [58].

For HDD data, performing a simple multivariable regression in high dimensions is typically not feasible. Therefore, most procedures for handling missing data in the HDD setting either involve a phase for selecting for imputation only those variables that are deemed important or trying to use some regularized regression [59] instead of standard multivariable regression. The handling of missing data in HDD settings is an active topic of research. Many tailor-made imputation algorithms have already been developed; for an early overview in the context of for gene expression measurements, see [60] (Table 11).

**Table 11:** Method for imputation of missing data: Multiple imputation

**Multiple imputation**
Multiple imputation is a widely used approach for handling missing data under the MAR scenario. It uses a regression model based on the available variables to predict the missing values. In an iterative fashion, missing values of a specific variable are predicted using a regression model that depends on the other observed variables, and the resulting predicted value is used in the main regression model. To account for the uncertainty in the imputation, multiple imputed datasets are generated and then analyzed, and the results are summarized according to “Rubin’s rule” [61]. Software for multiple imputation is widespread in major statistical packages. As described above, for HDD, before applying multiple imputation, often a pre-selection of variables is advisable
Future directions for HDD analysis include a more detailed look at MAR settings (as all procedures provided so far are fully justified only when the MCAR assumption is tenable), the addition of auxiliary information for specifying the imputation model, and development of analysis methods that can directly cope with missing values, such as robust PCA and random forests. The best method depends also on the analysis goal, such as cluster analysis or developing a prediction model

### EDA: Exploratory data analysis

When performing statistical analyses, it is important to distinguish between exploratory data analysis (EDA) and confirmatory data analysis, as this has important consequences both for the selection of appropriate analytical methods and for the correct interpretation of the results. The starting point for confirmatory analysis is a hypothesis to be evaluated, whereas, in EDA the goal is to provide an unbiased view of the data. Insights from EDA may then lead to development of new hypotheses that can be evaluated in subsequent confirmatory analyses on independent data.

Caution is necessary when performing statistical inference (e.g., feature selection as described in section “TEST: Identification of informative variables and multiple testing”) or model performance assessment following EDA when decisions to remove or modify observations from the analysis might depend on the observed relationships one is trying to confirm. For example, if outlier observations are removed from a dataset, the performance of a prediction model built only on the remaining observations is most probably an overly optimistic estimate of what the model performance would be on an independent dataset, which might contain different outliers.

Two major analytical goals for EDA are (1) to identify interesting data characteristics such as variables with extreme values, associations between variables, or representative subjects with usual values of variables, and (2) to gain insight into the structure of the data. Note that many of the methods used in EDA are also applied in IDA (like PCA; see section “IDA2.4: Graphical displays”). In this section, we focus on methods that are more specific to EDA. Note that many of the methods described in this section are generally designed and suitable for continuous data; only some can also be applied for discrete data.

#### EDA1: Identify interesting data characteristics

EDA can assist a researcher to identify interesting data characteristics that may lead to generation of specific scientific hypotheses that can be more fully evaluated in subsequent studies. Through EDA, a researcher might identify variables exhibiting extreme values or study subjects (observations) having extreme values of one or more variables or unusual combinations of values of two or more variables. EDA might also reveal intriguing associations between variables (e.g., levels of a certain protein tend to differ between two phenotypic classes). The two main classes of exploratory methods for identifying such interesting data characteristics are graphical displays and inspection of descriptive univariate and multivariate summary statistics. Graphical displays are discussed in sections “IDA2.1: Descriptive statistics,” “IDA2.4: Graphical displays,” and “EDA1.1: Graphical displays,” whereas descriptive statistics were already described in section “IDA2.1: Descriptive statistics” as tools for the initial data analysis (IDA). It should be noted that due to the potential for identification of many false positive signals in the HDD setting, findings from large-scale comparisons of descriptive summary statistics are often tempered by application of multiple testing methods as described later in section “TEST: Identification of informative variables and multiple testing,” even though the original intent was exploratory analysis.

To identify interesting data characteristics in low-dimensional data via visual or graphical methods, it is usually possible to inspect simple summary statistics and graphical displays of distributions of variables one, two, or three at a time, but for HDD this approach quickly becomes infeasible. For instance, the number of scatterplots for all pairs of *p* variables is *p*(*p* − 1)/2, which already exceeds 1000 when *p* exceeds 45. Visual identification of interesting characteristics of HDD typically requires specialized graphical displays or reduction of data dimensionality.

##### EDA1.1: Graphical displays

As mentioned in section “IDA2.4: Graphical displays,” one can use principal components (PCs) for exploratory analysis by first summarizing the information included in all variables through calculation of PC scores (which are linear combinations of the original variables) and then plotting in two or three dimensions the first several PC scores that capture the majority of variability in the data. This may allow identification of clusters of observations or individual observations with unusual configurations of variables warranting further inspection.

Another goal for HDD visualization is to produce a display in lower dimensions that preserves the distances (more generally degrees of “dissimilarity”) between observations such that the closest points remain the closest and the furthest remain the furthest. Alternative data reduction techniques have been developed to achieve this goal. These methods aim to translate the data in such a way that dissimilarities among points in the lower-dimensional space are as proportional as possible to those quantified in the original (high-dimensional) space. One such technique, multidimensional scaling, is described below. A variation of multidimensional scaling not discussed here is correspondence analysis, which is suitable for categorical variables and shows the relationships between variables based on data specified in a contingency table. Cox and Cox [62] provide descriptions of both multidimensional scaling and correspondence analysis (Table 12).

**Table 12:** Methods for graphical displays: Multidimensional scaling, t-SNE, UMAP, neural networks

**Fig. 10:**
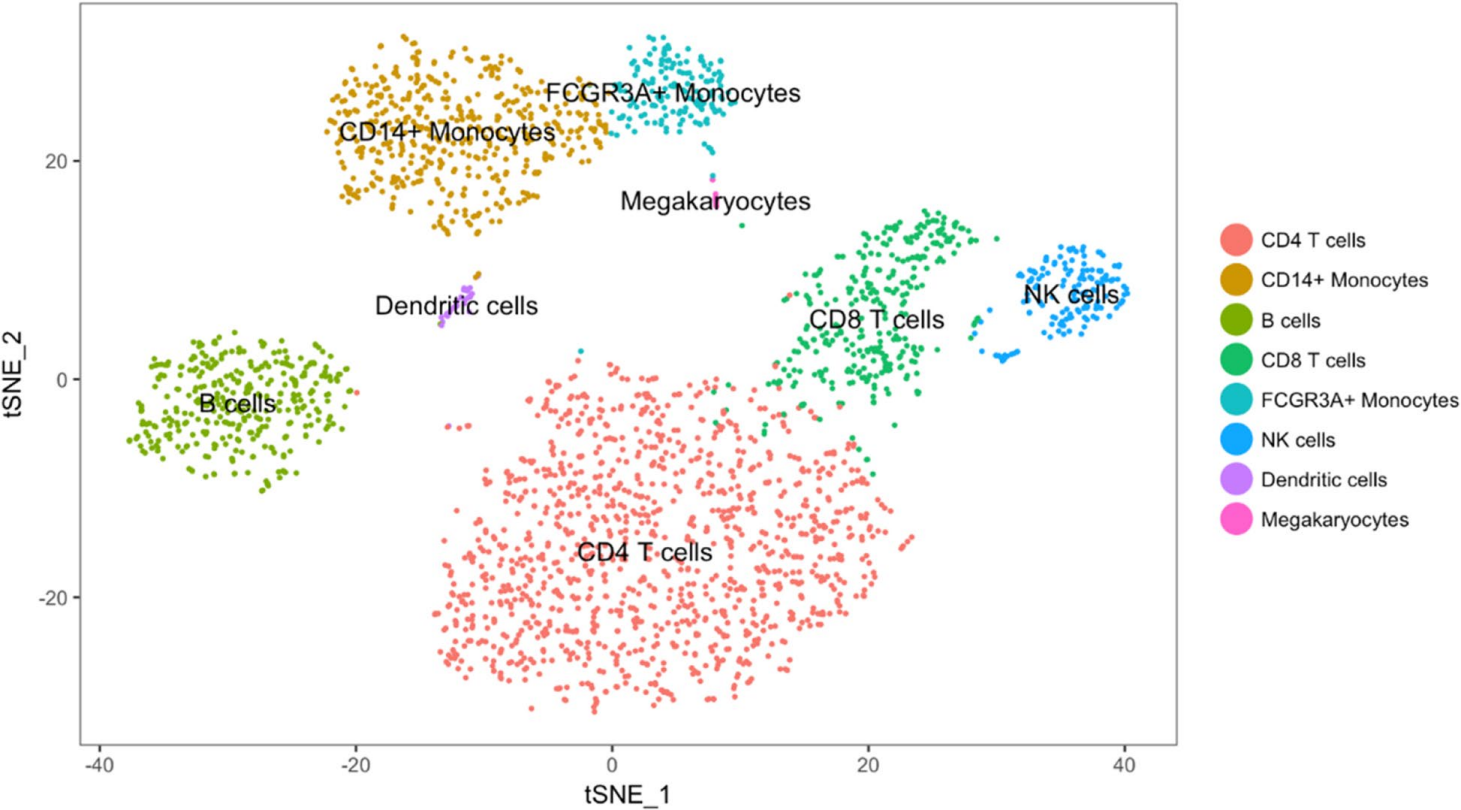
Two-dimensional visualization of a high-dimensional dataset using t-SNE. The dataset consists of 2700 single cells (peripheral blood mononuclear cells) that were sequenced on an Illumina NextSeq 500. The dataset is freely available from 10X Genomics. Points are colored by cell type. The plot shows that the cell types are locally well separated. Source: [71]

**Multidimensional scaling (MDS)**
Multidimensional scaling requires as input a distance matrix with elements corresponding to distances between all pairs of observations calculated in the original (high-dimensional) space, and the lower dimension space (often two-dimensional) to which the data should be projected is specified. A representation of the data points in the lower-dimensional space, called an embedding, is constructed such that the distances between pairs of observations are preserved as much as possible. Functions that quantify the level of agreement between pairwise distances before and after dimension reduction are called stress functions. MDS implements mathematical algorithms to minimize the specified stress function
Classical Multidimensional Scaling was first introduced by Torgerson [63]. Mathematically, it uses an eigenvalue decomposition of a transformed distance matrix to find an embedding. Torgerson [63] set out the foundations for this work, but further developments of the technique associated with the name principal coordinates analysis are attributed to Gower [64]. While Classical Multidimensional Scaling uses eigenvector decomposition to embed the data, non-Metric Multidimensional Scaling (nMDS) [65] uses optimization methods
**T-Distributed Stochastic Neighbor Embedding (t-SNE)**
Some newer approaches to derive lower-dimensional representations of data avoid the restriction of PCA, which requires the new coordinates to be linear transformations of the original. One popular approach is t-SNE [66], which is a variation of Stochastic Neighbor Embedding (SNE) [67]. It is the most commonly used technique in single-cell RNA-Seq analysis. t-SNE explicitly optimizes a loss function, by minimizing the Kullback–Leibler divergence between the distributions of pairwise differences between observations (subjects) in the original space and the low-dimensional space. PCA plots, which are typically based on the first two or three principal component scores, focus on preserving the distances between data points widely separated in high-dimensional space, whereas t-SNE aims to provide representations that preserve the distances between nearby data points. This means that t-SNE reduces the dimensionality of data mainly based on local properties of the data. t-SNE requires the specification of a tunable parameter known as “perplexity” which can be interpreted as a guess for the number of the effective neighbors (number of neighbors that are considered close). Figure 10 shows the result of t-SNE on a dataset with eight classes
**Uniform manifold approximation and projection (UMAP)**
t-SNE has been shown to efficiently reveal local data structure and was widely used for identifying subgroups of populations in cytometry and transcriptomic data. However, it has some limitations. It does not preserve well the global structure of the data, i.e., relations between observations that are far apart are not captured well by the low-dimensional representation. A further drawback is large computation time for HDD, especially for very large sample size *n*. A newer approach called uniform manifold approximation and projection (UMAP) [68] overcomes some of these limitations by using a different probability distribution in high dimensions. In particular, construction of an initial neighborhood graph is more sophisticated, e.g., by incorporating weights that reflect uncertainty. In addition, UMAP directly uses the number of nearest neighbors instead of the perplexity as tuning parameter, thus making tuning more transparent. On real data, UMAP has been shown to preserve as much of the local and more of the global data structure than t-SNE, with more reproducible results and shorter run time [69]
**Neural networks**
Neural networks provide another way to identify non-linear transformations to obtain lower-dimensional representations of HDD, which in many cases outperform simple linear transformations [70]. The concept is briefly described in section “PRED1.5: Algorithms” in the context of reducing the number of variables in preparation for development of prediction models or algorithms. Yet, research is ongoing to determine how best to develop low-dimensional representations and corresponding derived variables, and which of those derived variables might be most suitable depending on their subsequent use for statistical modelling or other purposes

#### EDA2: Gain insight into the data structure

A global depiction of data to identify structure, including patterns or motifs, is another major goal of exploratory data analysis for HDD. Here, data structure is understood in a general sense, it refers to many aspects of the data that concern the arrangement or interrelation of the observations or variables of a dataset. Although a natural first step is to look at marginal distributions (e.g., univariate and bivariate) of all variables across observations, this approach is generally not feasible for HDD for reasons discussed above. Further, some structure may involve many different variables and not be discernible by examination of univariate, bivariate, or even trivariate distributions.

The data visualization techniques described in section “EDA1.1: Graphical displays” are often supplemented with additional approaches geared toward detection of certain kinds of structure, for example clusters. The goal of cluster analysis is to identify subgroups of observations or variables that are similar to each other, but different from others. Identification of prototypical observations to characterize each cluster might be of interest. The structure might also be multi-level. In this section, we focus on techniques that are useful to uncover structure that might be missed by examining only marginal distributions or low-dimensional representations of HDD.

##### EDA2.1: Cluster analysis

The goal of a cluster analysis is to assemble objects (observations or variables) into subgroups, termed clusters, such that similarities between members within the clusters are high (or, equivalently, distances are small), compared to similarities between members from different clusters. Sometimes, the goal is only to find dense, i.e., heavily populated, regions in the data space that correspond to modes of the data distribution. Alternatively, there may be interest in fully characterizing the structure. Cluster analyses typically require choice of a similarity metric (or, alternatively, distance metric) for pairs of objects (sometimes also for pairs of clusters), a clustering algorithm, and a criterion to determine the number of clusters. Some clustering approaches that have been successfully used for low-dimensional data, e.g., mixtures of low-dimensional parametric probability distributions such as multivariate normal mixtures, either cannot be applied at all or perform very poorly in the HDD setting. Approaches not suitable for HDD are not further discussed here.

For comparing similarity of objects (either variables or observations), the Pearson correlation coefficient or Euclidean distance are the most popular metrics. The Pearson correlation does not depend on the scale of the variables, but the Euclidean distance does. If each of the variables characterizing an object is first standardized across the set of objects (subtract mean and divide by standard deviation), then use of Pearson correlation and Euclidean distance metrics will produce equivalent results. The measure should be chosen deliberately. If only relative levels of the values are important, then Pearson correlation is suitable, but if absolute values matter, then Euclidean distance is appropriate. It is important to note that both metrics tend to be more heavily influenced by a few large differences or deviations than by a series of small ones because the values are squared. An important modification of the Pearson correlation is the Spearman (rank) correlation, where values of observations are first replaced by their corresponding ranks before calculating the Pearson correlation. With this adjustment, the results are less heavily influenced by extreme data values.

In high-dimensional spaces, data are typically quite sparse. This means that distances between objects become large, a phenomenon often referred to as the curse of dimensionality. Therefore, the distance metrics may be prone to exaggeration by a few distant objects. Strategies to help avoid this problem include use of data reduction or variable selection before clustering (see section “IDA2.4: Graphical displays” for graphical displays for dimension reduction and section “PRED1.2: Variable selection.” for variable selection and dimension reduction in the context of improving prediction models).

Clustering algorithms can be divided into hierarchical and partitioning methods. In hierarchical clustering, observations are iteratively grouped together into larger clusters (agglomerative hierarchical clustering) or clusters are subdivided into smaller clusters (divisive hierarchical clustering). Centroid-based so-called partitioning algorithms aggregate the observations around specific points (the centroids) such that observations related to the same centroid are as similar as possible, and observations related to different centroids as different as possible. Hierarchical clustering algorithms provide a clustering for any number of clusters, whereas partitioning methods require an initial choice about the number of clusters present in the data. The most popular clustering algorithms are described in Table 13.

**Table 13:** Methods for cluster analysis: Hierarchical clustering, k-means, PAM

**Fig. 11:**
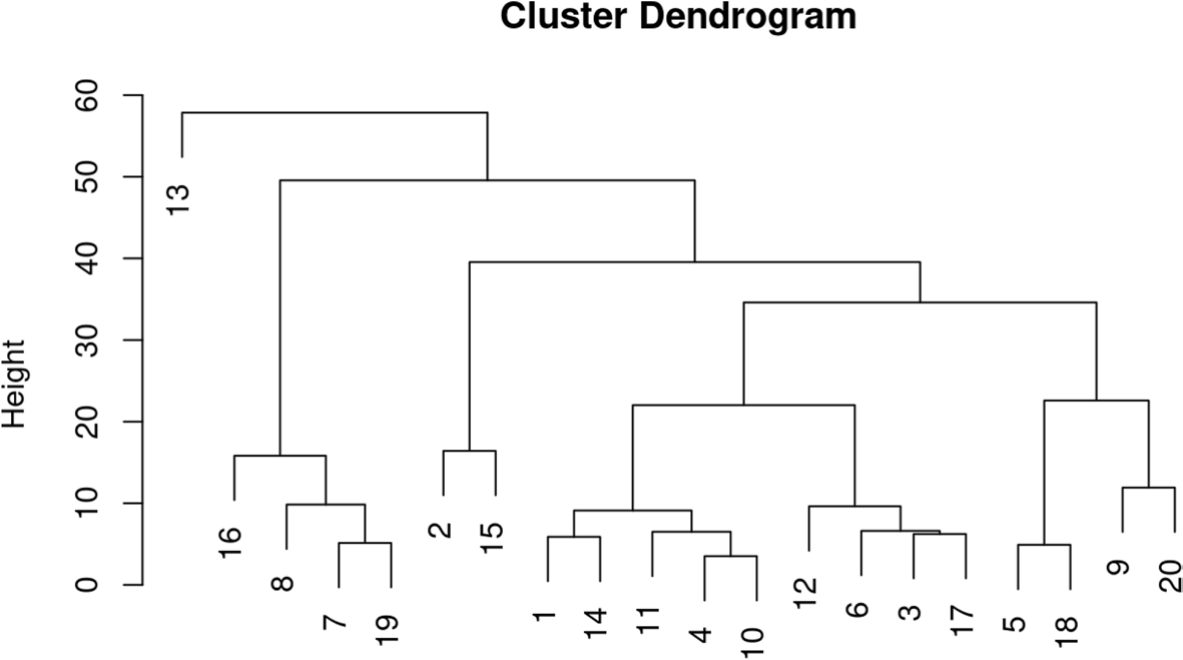
Hierarchical clustering result displayed in a dendrogram, where heights in the tree at which the clusters are merged correspond to the between-cluster distances. Source: [73]

**Fig. 12:**
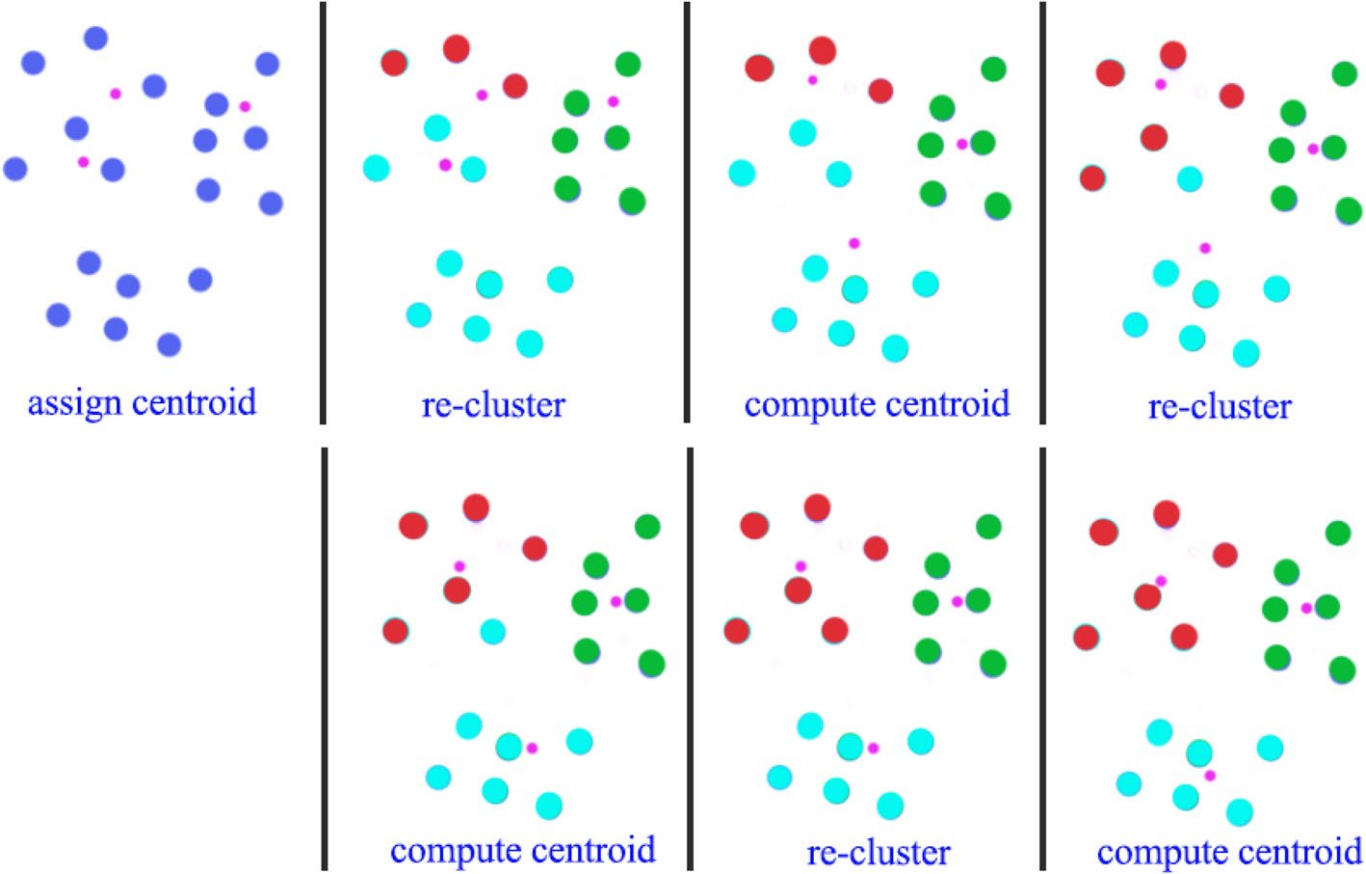
Visualization of the k-means algorithm with an example. Iteratively, observations are assigned to the cluster for which the squared Euclidean distance from the observation to the cluster centroid is minimized, and cluster centroids are computed based on the current cluster memberships. The iterative process continues until no observations are reassigned (as in the case of the last iteration in the figure). Source: [75]

**Hierarchical clustering**
Hierarchical clustering is a popular class of clustering algorithms, mostly in an agglomerative version, where initially all objects are assigned to their own cluster, and then iteratively, the two most similar clusters are joined, representing a new node of the clustering tree [72]. The similarities between the clusters are recalculated, and the process is repeated until all observations are in the same cluster. The distance metric to be used for comparing two individual objects is specified by the researcher. For defining distances between two clusters of objects, there are also several options. In hierarchical clustering, the approach for measuring between-cluster distance is referred to as the linkage method. Single linkage specifies the distance between two clusters as the closest distance between the objects from two clusters; average linkage calculates the mean of those distances, and complete linkage specifies the largest distance. Single linkage has the disadvantage that it tends to generate long thin clusters, whereas complete linkage tends to yield clusters that are more compact, and average linkage typically produces clusters with compactness somewhere in between. Hierarchical clustering results are often displayed in a tree-like structure called a dendrogram. A dendrogram is viewed from the bottom up, with each object beginning in its own cluster as the terminal end of a branch and eventually being merged with other objects as clusters are formed climbing up the branches of the tree toward the root where all objects are combined into one cluster. The heights in the tree at which the clusters are merged correspond to the between-cluster distances. Cutting the tree at a particular height defines a number of clusters. Although the hierarchical structure displayed in the dendrogram may seem appealing, it should be interpreted with caution as there can be substantial information loss incurred as a result of enforcing a flattened tree structure. Figure 11 [73] shows an example for a dendrogram resulting from hierarchical clustering
**k-means**
A popular partitioning clustering algorithm is k-means [74]. For its traditional implementation, the researcher must specify the number of clusters. First, random objects are chosen as initial centroids for the clusters. Then the algorithm proceeds by iterating between two steps, (i) comparing each observation to the mean of each cluster (centroid) and assigning it to the cluster for which the squared Euclidean distance from the observation to the cluster centroid is minimized, and (ii) recalculating cluster centroids based on the current cluster memberships. The iterative process continues until no observations are reassigned. k-means is not guaranteed to converge to the optimal cluster assignments that minimize the sum of within-cluster variances, and it can be strongly influenced by the selected number of clusters and initial cluster centroids. Nonetheless, it is a relatively simple algorithm to understand and implement and is widely used. Figure 12 [75] visualizes the k-means algorithm with an example
**PAM**
Several important extensions and generalizations of k-means have been developed. PAM (partitioning around medoids, [76]) allows using arbitrary distances instead of Euclidean distance, and instead of mathematically calculated centroids, actual observations are selected as prototypes of clusters. The algorithm iteratively improves a starting solution with respect to the sum of distances of all observations to their corresponding prototypes, until no improvement can be obtained by replacing one current prototype with another observation

Other methods for cluster analysis applied to biomedical data include fuzzy clustering and SOMs (self-organizing maps). In fuzzy clustering, objects can belong to multiple clusters. In SOMs (a type of neural networks first introduced by Kohonen [77]), a meaningful topology (special relationships) between the cluster prototypes is assumed. This means that the clusters can be visualized as a two-dimensional “map,” so that observations in proximate clusters have more similar values than observations in clusters that are more distant. Since the assumptions for SOMs are not guaranteed to hold, the interpretation can easily be misleading, such that SOMs should only be used by experts in this field. In addition, SOMs can be very sensitive to starting node configurations.

For HDD, the computer runtime of such partitioning algorithms can present a challenge. For example, PAM cannot be applied if the number of objects to be clustered is very large, i.e., for clustering variables in omics data or for clustering observations in large health records data. This challenge motivated development of the algorithm CLARA (Clustering Large Applications) [78], which works on subsamples of the data. Distribution-based clustering methods provide another alternative where probabilistic distributions for the observations within the clusters are assumed (e.g., multivariate Gaussian in each cluster, but with different means and potentially different variances). Parameters of the mixture distribution are typically estimated with EM-type (expectation–maximization) iterative algorithms [79]. However, not only, but particularly for HDD, the distributional assumptions are often difficult to verify and the algorithms may not converge to a suitable solution. Therefore, clusters might not be identified at all, or the results could be misleading due to incorrect assumptions about the data distributions.

Results produced by clustering algorithms are difficult to evaluate and often require subjective judgement. The validity of the results depends on the notion of a cluster, which varies between clustering algorithms, and this ambiguity carries through to estimation of the number of clusters (Table 14).

**Table 14:** Methods for estimation of the number of clusters: Scree plots, silhouette values

**Fig. 13:**
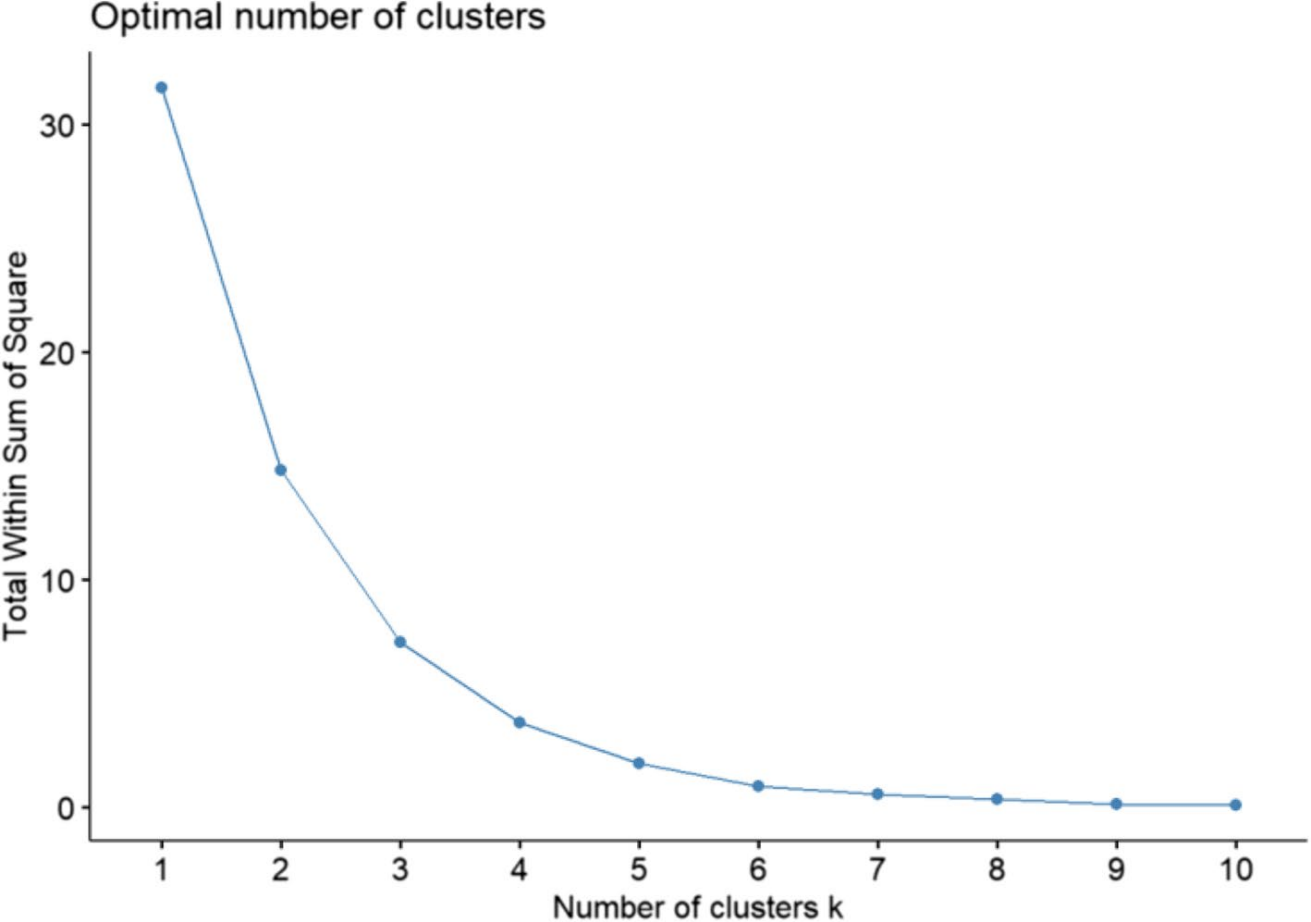
Example of a scree plot, which involves plotting some measure of within-cluster variation (here the total within sum of squares) on the *y*-axis and the number of clusters assumed in applying the algorithm on the *x*-axis. Source: [80]

**Fig. 14:**
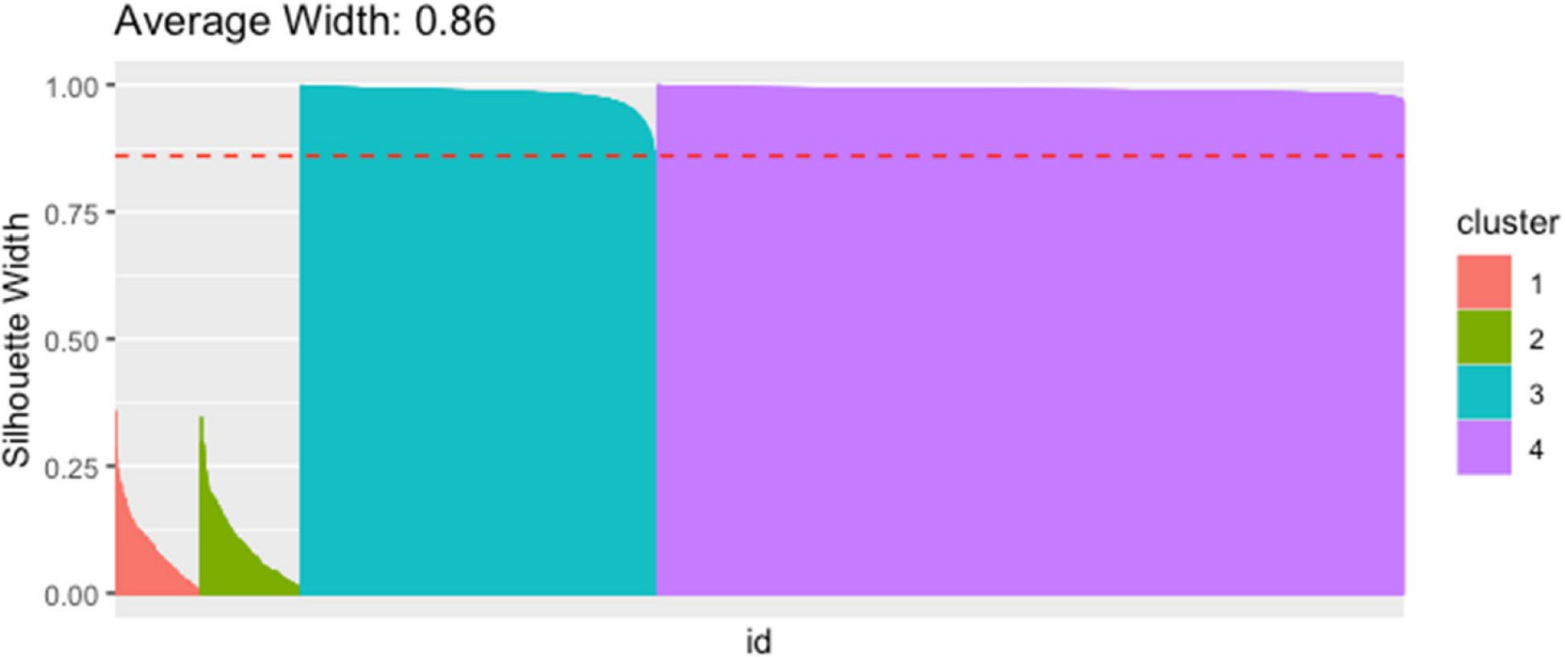
Silhouette values for observations that are grouped into four clusters. Observations are sorted along the *x*-axis by decreasing silhouette value, grouped by the four clusters. The silhouette values for the observations of the first two clusters have very low values, indicating two not well-separated clusters. Source: [82]

**Scree plots**
One traditional approach for estimation of the number of clusters is the construction of a scree plot, which involves plotting some measure of within-cluster variation on the *y*-axis and the number of clusters assumed in applying the algorithm on the *x*-axis. For hierarchical clustering, which does not require a priori specification of the number of clusters, a similar plot can be constructed by “cutting” the dendrogram at different levels corresponding to a range of numbers of clusters. The optimal number of clusters is determined by visual inspection where a line connecting the points shows a kink and there is diminished reduction in within-cluster variation with increasing number of clusters. Noise accumulating over the variables in HDD coupled with no guarantee that applications of the algorithms identify the optimal clusterings may lead to scree plots that fail to reveal a strong indication for the number of clusters. Figure 13 [80] shows such a typical scree plot
**Silhouette values**
Silhouette values are numerical tools for estimating the number of clusters [81]. The silhouette value of a single observation measures how well the observation fits to its assigned cluster by comparing its average similarity to members of its own cluster to the average similarity to the next best cluster. It is scaled such that the value 1 corresponds to an optimal fit (similarities to members of own cluster extremely large compared to next best cluster) and − 1 to the worst case (similarities to members of own cluster extremely small compared to best other cluster). The average silhouette width (asw) is then defined as average of all single silhouette values, which quantifies the quality of the clustering result. The asw requires no distributional assumptions for the data. In contrast, when using distribution-based clustering, typically so-called information criteria are required for selecting the number of clusters. These balance the coherence of the clusters (as large as possible) and the number of clusters (as small as possible). Figure 14 [82] shows a silhouette plot that visualizes the silhouette values of observations that were grouped into four clusters

Some clustering methods have been specifically developed to handle the typical large storage requirements and long run times for HDD settings. For example, CAST (Cluster Affinity Search Technique) [83] is especially useful for large numbers of observations or variables. Iteratively, clusters are constructed as follows. Choose a randomly selected observation not already assigned to a cluster and assign it to a newly defined cluster. Then repeat the following two steps until the set of observations assigned to this new cluster no longer changes. Add unassigned observations with average similarity to the current cluster members above a predefined threshold, and remove observations with average similarity below this threshold.

Another method is subspace clustering [84], where first subsets of variables are identified (called subspaces) and clusters are determined by defining regions of values based only on these variables. Then, iteratively, lower-dimensional subspaces are combined to higher-dimensional ones. In biclustering (or two-way clustering), first introduced by Hartigan [85], simultaneously variables and observations are selected to generate clusters that do not depend on all variables at the same time. Again, heuristic and stable algorithms are required to find approximate solutions in acceptable time (see, e.g., [86]).

Many traditional clustering methods are best suited for continuous variables, but there are several examples of HDD that are not continuous. One example is count data such as generated by RNA-Seq. Some examples of clustering methods that have been specifically developed for count data include those of Witten [87] and Si et al. [88], which are based on Poisson or negative binomial distributions. Cluster analysis based on deep learning has also been proposed [89]. That approach trains a deep neural network, extracts the resulting hidden variables, and uses them as the basis for clustering using standard methods like k-means.

##### EDA2.2: Prototypical samples

Often it is useful to construct prototypical observations that represent subgroups of observations. Prototypical observations are, for example, identified by some clustering algorithms. The motivation is to allow visualization or provide a summary of relevant characteristics of subgroups of observations. These summaries can be interpreted in the biomedical context, for example as a description of the characteristics of a typical patient who responds well to a particular therapy. Prototypical samples can be selected as central observations in their respective subgroups, or they can be newly constructed. When applying a k-means algorithm to separate observations into K clusters, centroids of each cluster are natural choices for prototypes. Similar to the principles of many cluster analysis approaches (see section “EDA2.1: Cluster analysis”), the construction of prototypical observations is done such that they are simultaneously as similar as possible to the observations of the same subgroup (cluster) and as different as possible from the observations of the other subgroups. Bien and Tibshirani [90] provide a nice overview of available methods, although their review is limited to classification problems. Prototypical observations can also be used to represent classes and then to predict the class of a new observation based on the similarities with these prototypical samples (see also section “PRED: Prediction”).

### TEST: Identification of informative variables and multiple testing

In HDD analysis, one is often interested in identifying, among a large number of candidate variables, “informative variables.” These are associated with an outcome or with a set of other phenotype variables that characterize the study subjects. For example, one might wish to characterize which single-nucleotide polymorphisms are more often present in patients who experience severe side effects from a particular drug compared to patients without severe side effects. In drug sensitivity screens performed on bacterial cultures, one might aim to identify bacterial genes with expression significantly associated with degree of sensitivity to a new antibiotic. When comparing individuals with a particular disease to healthy volunteers, one might wish to identify circulating proteins that are present in different abundance. In all these cases, evaluation of the associations might be accomplished by conducting many statistical hypothesis tests, one per candidate variable. This represents a *multiple testing* situation.

Multiple testing scenarios commonly encountered in biomedical studies with HDD are divided here into three categories. Scenarios that consider each candidate variable individually and perform a similar evaluation or statistical test for each include the following three cases: (i) Identification of variables among a set of candidates that are associated with a single outcome or phenotype variable, i.e., related to outcome or phenotype classes (categorical) or correlated with a continuous phenotype variable or time-to-event outcome. (ii) Identification of candidate variables with a trajectory over time affected by experimental factors or exhibiting a prescribed pattern. (iii) Identification of candidate variables that are associated with a prespecified set of other variables, i.e., where the candidate variables are considered as dependent variables and the set of prespecified variables as independent “predictor” variables. To illustrate the concepts, much of the discussion here will focus on a simple example of scenario (i) in which two classes are being compared with respect to a very large number of variables. Methods discussed for scenario (i) that can be extended straightforwardly to scenarios (ii) and (iii) are noted.

Scientific goals may go beyond simply providing a list of individual variables exhibiting associations with an outcome, a phenotype, a collection of prespecified variables, or patterns over time. Frequently, there is interest in more globally characterizing the variables that were included in the identified list. For example, genes are organized into interconnected biological pathways. Expression of two different genes might exhibit similar associations because they are both regulated by certain other genes, because one lies downstream of the other in the same biological pathway, or because their products serve similar biological functions. Established organizational structures might be described by gene taxonomies such as Gene Ontology [91], KEGG [92], or BioCarta [93]. Gene set enrichment analysis (see section “TEST3: Identify informative groups of variables”) refers to approaches that exploit these expected associations. They were first proposed in the omics field for use with HDD gene expression data. Although these enrichment analysis strategies could be applied in a variety of HDD settings, subsequent discussion of these methods will be based on examples with high-dimensional gene expression data for which the concept of enrichment is intuitively clear.

#### TEST1: Identify variables informative for an outcome

##### TEST1.1: Test statistics: Hypothesis testing for a single variable

Before discussing multiple testing procedures, it is helpful to briefly review basic concepts in statistical hypothesis testing involving a single variable. A hypothesis test aims to decide whether the data support or refute a stated “null hypothesis.” Typical examples of simple null hypotheses are that the distribution of a variable is not different between two or more groups or that a variable is not associated with another variable. A hypothesis test is based on some statistic that will reflect strength of evidence for or against the null hypothesis. Knowing the distribution of the test statistic (e.g., normal distribution or binomial distribution) allows one to construct a hypothesis test based on that statistic for which the probability of drawing an incorrect conclusion is controlled. Type I error refers to erroneously rejecting the null hypothesis when it is actually true. Type II error refers to failing to reject the null hypothesis when it is actually false. Statistical power is defined as one minus the type II error. In general, one wants to control the probability of a type I error, denoted *α*, at a small value, while maintaining acceptably high power (or low type II error). A conventional choice of $\alpha$ for the single variable setting is 0.05, which means that the probability of a false positive decision, i.e., falsely rejecting the null hypothesis when it is true, is 0.05.

Hypothesis testing is often operationalized by calculation of a *p*-value from the observed data, which estimates the probability of observing a value of the test statistic that is at least as extreme as that observed, assuming that the null hypothesis was true. (Note the correct definition of a *p*-value stated here, in contrast to the common misinterpretation of a *p*-value as the probability that H_0_ is true). A significance test is performed by comparing the computed *p*-value to the prespecified *α* level. When the *p*-value is less than or equal to *α* (e.g., 0.05 in the conventional setting), the null hypothesis is rejected; otherwise, it cannot be rejected.

It should be mentioned that sometimes the goal of a scientific study is to estimate certain parameters of interest, for example means or correlations, rather than to test hypotheses. In estimation settings, it is generally desired to provide intervals of uncertainty, such as confidence intervals, to accompany parameter estimates. Although errors in hypothesis testing have some relation to confidence interval coverage probabilities, most of the multiple testing procedures discussed in this section are not readily applicable to multiple estimation. Multiple estimation procedures are beyond the scope of the present discussion.

The *t*-test is an example of a widely used statistical test for a single variable. It is the basis for the modelling approaches described below that are extensions of hypothesis testing to multiple variables. Extensions particularly developed for HDD include limma, edgeR, and Deseq2, as discussed in section “TEST1.2: Modelling approaches: Hypothesis testing for multiple variables.”

Calculation of a *p*-value usually requires assumptions about the distribution of the test statistic. Sometimes that distribution can be derived from assumptions about the distributions of the variables. For example, the statistic of the *t*-test can be shown to have a *t*-distribution when the variables are normally distributed, and the within-group variances are the same for the classes being compared. Similar requirements hold for F-tests in analysis of variance and statistics associated with standard linear regression analysis. Although one can never be certain if these assumptions hold for real data, many test statistics can be shown by theoretical arguments to have an approximate normal distribution when sample size is sufficiently large (referred to as “asymptotic” approximation). An example asymptotic property is that a *t*-statistic has an approximate normal distribution for large samples size, even if the data are not normally distributed. Nonetheless, extra caution is necessary in the setting of HDD where the requirements for sample size to qualify as “large” are far greater. Extremes of a test statistic’s distribution are particularly prone to departures from data distributional assumptions, and this is exactly where accuracy is needed most when calculating the very small *p*-values upon which many multiple testing procedures for HDD rely.

When validity of assumptions required for familiar statistical tests is uncertain, for example that the data follow a normal distribution for the *t*-test or F-test, alternative tests broadly referred to as nonparametric tests may be preferable. Wilcoxon rank sum (equivalent to Mann–Whitney U) and signed rank tests are nonparametric alternatives to the two-sample *t*-test and paired *t*-test, respectively; the Kruskal–Wallis test is an alternative to the F-test in one-way ANOVA. These nonparametric tests are robust to outliers and do not require data to be normally distributed; nor do they require that their distribution is fully characterized by two parameters in the way that a mean and variance characterize a normal distribution. Many nonparametric tests are based on ranks of observed data rather than their actual values. Permutation tests, as described in Table 15 and below, comprise another class of nonparametric tests and are more generally applicable than rank-based tests.

**Table 15:** Methods for hypothesis testing for a single variable: *t*-test, permutation test

***t*-test**
The *t*-test is a standard test for comparing the means of two groups, for continuous outcomes (e.g., blood pressure or tumor size after therapy for a treatment and a control group, or expression values of a gene for two patient groups with different diseases). The null hypothesis is that the true difference between the group means is 0, and the alternative hypothesis is that it is not 0 (two-sided testing). The *t*-statistic underlying the usual *t*-test equals the ratio of the observed mean difference and a pooled standard error of both groups. It is important to note that validity of a statistical test depends on assumptions that should be checked. For this *t*-test, assumptions include independence of the observations, approximate normal distribution of the variable in each group and similar variance of the variable irrespective of group. *t*-tests tend to be sensitive to outliers, and in such situations, alternative nonparametric tests may be preferred. Extensions include the Welch test, if group variances are not assumed equal, and one-way ANOVA (analysis of variance), when more than two groups are compared
**Permutation test**
The idea behind a permutation test is to scramble the data to mimic a null hypothesis situation in which a variable is not associated with a particular outcome or phenotype. For the simple example of comparing the distribution of a variable between two phenotype classes, a permutation test would randomly scramble or re-assign class labels to the collection of observations. For each data permutation, the test statistic is calculated and recorded. After this statistic has been calculated on many permuted versions of the data, a *p*-value can be computed as the number of permutations on which the calculated test statistic was as extreme or more than the test statistic calculated of the original data

A word of caution is in order to emphasize that correct permutation of the data is critical to validity of a permutation test. The permutations must preserve any structure in the data that is unrelated to the null hypothesis. For instance, if the goal is to test whether the mean of a variable is different between groups, but it is thought that the variances are different, then the simple permutation test described for the two-group comparison will not be appropriate because the permutations will change the variances as well as the means. If the groups are paired, e.g., variables are measured both before and after each subject receives an experimental drug, then the permutations would have to preserve that pairing by randomly “flipping” the before and after measurements within patients. Correct permutation might not be easy, or even feasible, for regression models with multiple predictors. For example, naively permuting the outcomes in a logistic or Cox regression model with many predictors to provide test statistics for *individual* predictor variables (adjusted for the other variables) would not provide valid permutation *p*-values because the correlation structure of the data, e.g., correlations of the outcome with other variables that are not the focus of the test, would not be preserved. Anderson and Legendre [94] discuss appropriateness and performance of various permutation testing strategies in the context of testing partial regression coefficients in multivariable regression models.

Nonparametric methods have advantages and disadvantages. In the context of statistical tests, their main advantages include their applicability in situations where little is understood about the likely distribution of the data, and their robustness to oddities in the data such as outliers. The main disadvantage of nonparametric methods is their reduced statistical power, particularly for small samples sizes, compared to a parametric test when distributional assumptions of that test are actually met. For HDD settings, parametric tests have additional appeal, when reasonably justified, due to the possibility to “borrow information” across variables by modelling relationships of parameters (e.g., means or variances) across variable-specific distributions; modelling approaches such as those discussed in section “TEST1.2: Modelling approaches: Hypothesis testing for multiple variables” can greatly increase statistical power for testing multiple hypotheses.

##### TEST1.2: Modelling approaches: Hypothesis testing for multiple variables

In the scenarios (i)-(iii) described in the introduction of section “TEST: Identification of informative variables and multiple testing”, the number of statistical analyses performed is equal to the number of variables. For omics data, the number of variables is often in the range of tens of thousands or even millions. Direct application of standard hypothesis testing approaches to each variable in the setting of HDD is problematic. As an illustration, consider conducting several thousand statistical tests (one per candidate variable), each using the classical *α* level of 0.05 to test for significance of an association between a single variable and an outcome or phenotype of interest. If the truth were that none of the candidate variables had an association with the outcome or phenotype of interest, then, on average, testing 20,000 variables would lead to 1000 false positive test results (0.05 times the 20,000 variables tested), clearly an unacceptably large number that would limit interpretability of the results. Control of the number of false positives, often termed “false discoveries” in the setting of HDD, is critical.

Several challenges are encountered in multiple testing for HDD omics data. One is that in order to control false positives when a very large number of statistical tests are performed, small *α* levels must be used, which limits statistical power. Another challenge is the mathematical difficulty of dealing with joint distributions of certain variable types such as counts, which are commonly generated by newer omics technologies such as RNA-Seq. Furthermore, sample sizes are often insufficient to rely on classical statistical asymptotic (large sample size) theory to provide tractable approximate distributions of test statistics required to appropriately control type I and II errors. Finally, the classical approach of limiting false positives by controlling the overall probability of any false positive findings is overly stringent when extremely large numbers of tests are performed. These challenges have spawned a wealth of innovative statistical approaches for multiple testing with HDD, which are described in the sections that follow.

The earliest technologies for high-dimensional gene expression analysis based on microarray platforms quantified gene expression by fluorescence intensities. After logarithmic transformation, these continuous intensity values are typically well approximated by a normal distribution. Many of the early methods developed for statistical analysis of microarray data relied on normally distributed data, the simplest example being use of *t*-tests to identify lists of differentially expressed genes with varying degrees of type I error control. Sample size in these early studies was usually relatively small, making it difficult to adequately control false discoveries and still maintain sufficient statistical power. Some of these methods were ad hoc or limited to simple experimental settings such as two-group comparisons, but advances in statistical methodology led to improved approaches for the analysis of HDD gene expression data (Table 16).

**Table 16:** Methods for hypothesis testing for multiple variables in HDD: Limma, edgeR, DEseq2

**Limma**
Linear Models for Microarray Data (limma) developed by Smyth and colleagues [95, 96] and implemented in the R package limma was developed to address several challenges of multiple testing for HDD. Limma offers a unifying, statistically based framework for multiple testing that uses empirical Bayes shrinkage methods in the context of linear models. Initially popularized in the context of traditional gene expression analysis with microarrays, limma is based on normal distribution theory. It evolved from a procedure to modify *t*-statistics by “borrowing information” across variables to improve variance estimation and increase statistical power. Limma provides a way to balance the need for small type I errors for testing individual variables in HDD settings against statistical power to identify true discoveries. Designs more complex than simple two-group comparisons are easily accommodated by limma’s linear model framework. Although it was developed originally to identify differentially expressed genes for normalized measurements from microarrays, it has also been used successfully for analysis of data generated by other omics technologies, e.g., proteomics [97]
For simplicity of explanation, the focus of discussion here is how limma works in the context of simple two-group comparisons as an extension of the familiar *t*-test. Limma relies on the concept of borrowing information across a collection of similar variables (e.g., expression levels for the thousands of genes measured on a microarray). Many omics studies have relatively small sample size compared to the number of variables, so the idea of borrowing information across a very large number of variables is very attractive. If one can assume that the true variances across the many variables follow some overarching distribution, then variance estimates for individual variables that are imprecise due to small sample size can be made more precise by shrinking them toward a variance estimate that is pooled from all variables. The amount of shrinkage depends on the distribution estimated (empirical Bayes) or assumed (Bayes) for the true variances. Limma is based on an empirical Bayes approach that assumes normally distributed variables and shrinks the individual variances toward the mean of the estimated distribution of true variances
Out of this empirical Bayes framework comes the moderated *t*-statistic, which is similar in form to the usual *t*-statistic, but with an adjusted estimate of standard deviation for each variable that has been shrunk toward the mean of the distribution of variances, replacing the usual sample standard deviation estimate in the denominator. These shrunken estimates are more precise as reflected in larger degrees of freedom achieved by “gathering strength” across the many variables, resulting in higher statistical power to identify true discoveries
An additional advantage of limma is the complexity of experimental designs that it can handle. Many extensions beyond two class comparison problems can be accommodated by the linear model framework. Comparisons can be made between more than two classes, including linear contrasts, for example to assess for linear trends in means across classes. In addition, limma offers a powerful set of tools to address a broad range of experimental settings in which data can be reasonably represented by a Gaussian linear model. Included in the limma framework are factorial designs, which consist of two or more factors with levels (discrete possible values), for which all combinations across the factors are investigated. This allows the analysis of main effects and interactions between variables
The evolution of technologies for gene expression analysis from microarrays to sequencing-based approaches such as RNA-Seq presented new statistical challenges for HDD analysis. Gene expression measurements generated by these newer technologies are typically count data rather than continuous intensity values as for microarray technologies. Count data are generally not compatible with assumptions of normally distributed data on which limma relies. For example, RNA-Seq measures the number of reads (DNA fragments) that map to specific genomic locations or features represented on a reference genome. Two extensions to limma were developed to address gene expression measurements expressed as counts. Limma-trend shrinks the gene-wise variances of the log-transformed count values toward a global mean–variance trend curve. Limma-voom extends this idea further by also taking into account global differences in counts between samples, for example due to different sequencing depths
Several other methods to analyze count data were developed independently of the limma extensions, with foundation on negative binomial models to characterize the distribution of count data. The negative binomial includes the Poisson as a special case and is generally preferred in the setting of modern gene expression analysis. It has greater flexibility for modelling variances of counts, particularly when those counts are not large or when the number of replicates for each biological group or condition is not large
**edgeR**
The edgeR procedure [98] assumes that the read count for a particular genomic feature follows a negative binomial (NB) distribution. Although a genomic variable of interest need not correspond exactly to a gene, in the following the term gene is used for simplicity of discussion. Much of the discussion is framed in terms of gene expression count data arising from RNA-Seq measurements, but the developers note that the methods implemented in edgeR apply more generally also to count data generated by other omics technologies, including ChIP-Seq for epigenetic marks and DNA methylation analyses
The measured count for gene *g* in sample *i* is assumed to follow a NB distribution with mean equal to the library size for that sample (total number of DNA fragments generated and mapped) multiplied by a parameter representing the relative abundance of gene *g* in the experimental group *j* to which sample *i* belongs. The variance of the count for a specific gene based on the NB distribution is assumed to be a function of the mean and a dispersion parameter; specifically, the variance is modeled as the sum of the technical variation and the biological variation. Technical variation for gene expression and other types of omics count data can usually be adequately modeled as a Poisson variable, but incorporating biological variability leads to additional variability. To incorporate this additional variability, an “overdispersion” term is introduced into the variance. Specifically, the variance of a count is modeled as the mean multiplied by the sum of one and the mean multiplied by a term that represents the coefficient of variation of biological variation between samples. This expression reflects a partition of the variance into contributions from technical and biological variation. When there is no biological variation between samples, e.g., when samples are true technical replicate sequencing runs from a single library produced for a sample, this variance reduces to the Poisson variance, which equals the mean. This model provides a flexible and intuitive expression for the variance and incorporates dependence of the variance on the mean as expected for count data
Using an empirical Bayes approach similar in flavor to that described for limma, the edgeR procedure borrows information across genes to shrink the gene-specific dispersion parameters toward a model describing the distribution of dispersion parameters. The simplest model is one in which all genes share a common dispersion parameter, which can be estimated from the data. Allowing greater flexibility, dispersion parameters can be modeled as a smooth trend as a function of average read count for each gene. To allow for further gene-specific reasons for variation in the count of a gene, empirical Bayes methods are employed to estimate weighted averages that combine gene-specific dispersion estimates with those arising from dispersion models, in this way “shrinking” gene-specific dispersion estimates toward the overall model
The edgeR software allows the user to compare gene expression between groups when there are replicate measurements in at least one of the groups and more generally when the group mean structure can be expressed as a linear model. Scientific questions of interest can be framed in terms of inferences about the relative abundance parameters in the linear model. For example, one might wish to compare relative abundance of a particular gene transcript in a group of samples taken from cell cultures that had not been exposed to a new drug to that in samples from cultures after exposure to the new drug. There could be interest in examining the pattern of change in relative abundance of the gene, sampling from a series of cultures that are exposed to the new drug for differing lengths of time. From the specified linear model and shrunken variance estimates, the edgeR software can perform gene-wise tests of significance, based on likelihood ratio statistics, for any parameters or contrasts of parameters in the mean model
**DEseq2**
DESeq2 [99] is another method for differential analysis of count data that is widely used. Performance of DEseq2 compares with edgeR in terms of false discovery control and statistical power to detect differentially expressed genes. It also uses a negative binomial model for the counts with variance expression that incorporates a dispersion parameter, as described for edgeR. Dispersion parameters are modeled across genes as a smooth curve depending on average gene expression strength. Using empirical Bayes methods, gene-specific dispersion parameters are shrunk toward the curve by an amount dependent on how close the individual dispersion estimates tend to be to the fitted smooth curve and the sample size (through the degrees of freedom)
A feature of DEseq2 that distinguishes it from other methods is incorporation of shrinkage into estimation of mean parameters. Shrinkage of mean parameters, e.g., fold-change, has appeal because researchers tend to find larger effects more convincing. Genes that attain statistically significant effects but exhibit small effect sizes are frequently manually filtered out due to concern that the significance could be due to random experimental noise. Shrinkage of fold-changes implemented by DESeq2 provides a more statistically based approach to address these less reliable findings, which are observed particularly often for genes with small counts. Additional useful features of DESEq2 include options for outlier detection

Sometimes a researcher is interested in identifying genes for which expression is *not* different between conditions, opposite the more typical goal to identify differentially expressed genes. This requires reversing the usual role of the null and alternative hypotheses. However, since it is impossible to statistically rule out very tiny effects, the null hypothesis that is tested for each gene is that its effect is larger than some user-specified minimum size. When implementing this procedure to identify genes with negligible effect, mean parameter shrinkage functions must be turned off.

#### TEST2: Multiple testing

Methods described in the previous section provide useful approaches to improve statistical power for testing individual variables (genes) and to appropriately model commonly encountered omics data. However, a final step is required to control false positives in HDD settings. Several multiple testing correction methods and their utility for HDD are discussed in this section.

##### TEST2.1: Control for false discoveries: Classical multiple testing corrections

A simple table illustrates the types of errors that can be encountered in multiple testing [100]. When testing *m* hypotheses, these are either true or false, and either rejected or not rejected, yielding four possibilities, which are displayed in Table 17 along with the numbers of hypotheses falling in each category.

**Table 17:** Contingency table describing outcomes of testing multiple null hypotheses

	**Null hypothesis truth status**		
**Test result**	True	False	Total
Rejected	*V*	*U*	*R*
Not rejected	*m*_*0*_* − V*	*m*_*1*_* − U*	*m − R*
Total	*m*_*0*_	*m*_*1*_	*m*

In Table 17, *m* represents the number of tests conducted; *R* represents the number rejected hypotheses;* V* represents the number of tests for which type I errors were committed, or the number of false positives; and *U* represents the number of tests that correctly rejected the null hypothesis, or the number of true positives. Further, *m*_*0*_ represents the total number of true null hypotheses; *m*_*1*_ the total number of false null hypotheses; and *m*_*1*_* − U* represents the number of tests for which type II errors were committed. The goal of a multiple testing procedure is to control *V* while not too severely limiting *U*. If *R* = *0*, then no type I error can be committed. If *m*_*0*_ = *m*, then rejection of any test constitutes a type I error and represents a false positive result.

Classical multiple testing corrections that aim to control false discoveries by using more stringent (smaller) “critical” levels for significance testing may work well in situations with a few dozen tests or less. However, they can be problematic for HDD because they may be too stringent and severely limit statistical power for detecting associations that truly exist, particularly when sample sizes are not large.

The simplest approach to controlling false discoveries is the classical Bonferroni correction, where the critical level is adjusted by dividing it by the number of tests performed (see Table 18). Bonferroni correction is very stringent for several reasons. First, it is designed to control what is known as *familywise error rate (FWER)*, which refers to globally controlling the probability that any of the tests results in a false discovery. In terms of the notation in Table 17, controlling the FWER at level *α* means requiring *P*(*V* > 0) ≤ *α.* Despite its conservativeness, Bonferroni adjustment has become the standard approach for genome-wide association studies to control the genome-wide significance level. This enforces stringent control on the probability that any of the hundreds of thousands of genomic variants typically studied is falsely identified as associated with the phenotype of interest. Second, a simple Bonferroni correction is conservative in that it does not leverage information about potential correlations between the test statistics; nor does it account for the ordering of the *p*-values when applying the significance-testing threshold. When evaluating *p*-values in order from smallest to largest, it is natural to require smaller critical levels for declaring significance earlier in the list. These limitations of the Bonferroni correction have motivated development of modified approaches that are less stringent, as discussed next.

**Table 18:** Methods for multiple testing corrections: Bonferroni correction, Holm’s procedure, Westfall-Young permutation procedure

**Bonferroni correction**
The Bonferroni correction specifies that when *m* statistical tests are conducted, each one should use a critical level of *α*/*m* where *α* is the desired type I error for the full collection of tests. For example, a Bonferroni correction applied in the setting of 10,000 hypothesis tests would require that an individual test reaches statistical significance at a critical level = 0.05/10,000 = 0.000005. Achieving this level of significance would require an extremely large sample size or effect size (e.g., magnitude of association) in order for an individual test to have reasonable power
**Holm’s procedure**
Order the *p*-values from smallest to largest as *p*_*(1)*_*, p*_*(2)*_*,..., p*_*(m)*_, where *m* is the number of tests. Beginning with *p*_*(1)*_, proceed in order, comparing each *p*_*(i)*_ to the critical value *α*/(*m-i* + *1)*. Stop the first time that *p*_*(i)*_ exceeds the critical value α/(*m-i* + *1)*. Call this index *j*. Declare all *p*-values *p*_*(1)*_*, p*_*(2)*_*,..., p*_*(j-1)*_ to be statistically significant
This procedure controls the FWER to be no more than *α*. It is clear from comparison of the sequential Holm critical values to the fixed Bonferroni critical value that the Holm procedure has the potential to reject more tests and therefore offers greater power, although when the number of tests *m* is very large, as often in HDD, the actual difference in critical values can be extremely small
**Westfall-Young permutation procedure**
The Westfall-Young permutation procedure [104] is a multivariate permutation procedure to control the FWER that is more efficient (powerful) than Bonferroni-like procedures (as Bonferroni and Holm’s procedure) in finding true discoveries. It exploits the correlations among variables, which are preserved in the permutation process, since all variables are permuted at the same time. The method is a step-down procedure similar to the Holm method. After *p*-values are calculated for all variables and ranked, multiple times class labels are permuted and corresponding *p*-values are calculated. Then the successive minima of these new *p*-values are retained and compared to the original *p*-values. For each variable, the proportion of number of permutations where the minimum new *p*-value is less than the original *p*-value is the adjusted *p*-value

Some adjusted versions of Bonferroni correction that take *p*-value ordering into account have been proposed. Some, such as those proposed by Hochberg [101] and Hommel [102], require assumptions about the joint distribution of the *p*-values such as the nature of correlations, and those are not discussed here. However, the approach proposed by Holm [103] provides a simple improvement on the Bonferroni method that allows critical values for significance testing to depend on the ordering of the *p*-values while, like Bonferroni, requiring no assumptions about the joint distribution of the *p*-values. Holm’s approach is described in Table 18.

Several other methods of controlling the FWER have been proposed that require additional assumptions about the nature of correlations between test statistics or might only control false positives under a global null in which all hypotheses are null. Such tests are not guaranteed to always control the FWER when these assumptions do not hold and will not be discussed further here.

An appealing aspect of multiple testing procedures that control FWER is that one can make statements about the probability that an individual test falsely rejects the null hypothesis. Because the probability that *any* test among a collection of tests falsely rejects must be at least as large as the probability that a single randomly chosen test falsely rejects, control of FWER at level *α* automatically guarantees control of the type I error at level *α* for each individual test.

An important caveat about any multiple testing correction method that is based on *p*-values is that it relies on the validity of the *p*-values or the validity of the corresponding test procedures. As noted in the discussion of test statistics above in section “TEST1: Identify variables informative for an outcome,” ensuring sufficient accuracy of *p*-values based on specific (parametric) distributions can be challenging in HDD settings. Permutation tests can provide distribution-free options for multiple testing in some situations. They also offer the flexibility to handle HDD with variables of different types, e.g., variables could be a mix of categorical, count, or continuous data. However, permutation tests can be problematic for multiple testing in HDD settings as well, as it can be very computationally intensive to accurately compute *p*-values that might be very small.

Multivariate permutation tests are permutation tests that are applied for testing multiple hypotheses simultaneously. For each hypothesis, a test statistic is calculated, for example for simultaneously comparing the distribution of many omics variables between two phenotype classes. As in the univariate case, class labels are randomly reassigned to the observations (keeping the full profile of measurements intact for each observation), and then a *p*-value for each variable is computed as the number of permutations on which the corresponding calculated test statistic is as extreme or more than the test statistic calculated of the original data. The popular Westfall-Young permutation procedure, as an example, is described in Table 18. Multiple testing procedures can be applied to the collection of permutation *p*-values to control false discoveries just as if the *p*-values had been computed assuming parametric distributions for the variable.

##### TEST2.2: Control for false discoveries: Methods motivated by HDD

Various multiple testing correction methods have been developed that are more appropriate for HDD than the classical Bonferroni-type methods. Usually, these approaches aim for a false discovery control that is less stringent than familywise error control, such as limiting the *percentage* of false discoveries (rather than aiming to avoid *any* false discoveries) in exchange for greater power to detect true discoveries. Many multiple testing methods for HDD are combined with methods such as those just discussed in section “TEST1.2: Modelling approaches: Hypothesis testing for multiple variables” that borrow information across variables (or tests) or that exploit correlations between candidate variables to increase statistical power. The growing amount of HDD stimulated development of a variety of innovative multiple testing procedures more appropriate for these data than traditional approaches.

To describe the various multiple testing approaches for HDD and the false discovery criteria that they control, it is helpful to focus again on one of the most frequent goals in omics data analysis, which is the identification of differentially expressed genes between two or more classes or conditions. The notation used in this section follows that defined in Table 17.

Aiming to control type I error in terms of the FWER through application of classical Bonferroni-type methods becomes extremely challenging with increasing dimension of HDD due to low statistical power, as already discussed. These challenges motivated consideration of alternatives to classical control of type I error, most commonly control of the false discovery rate (FDR). The popular FDR is in principle the expected proportion of false discoveries among the rejected tests and described in more detail below. The methods differ by the type of error they aim to control but share some operational aspects. Once the acceptable magnitude of error (e.g., FDR) has been specified, the (raw, uncorrected) *p*-values are calculated and next the variables are usually ranked based on their associated *p*-values. Those with *p*-values below a certain threshold are included in the list of the positive findings (rejecting their associated null hypotheses). This threshold can be fixed for all *p*-values, or it may depend on the ranking of *p*-value. Equivalently, the *p*-values can be adjusted and then compared to the desired level of error control. There are several methods for FDR control, which define in a different way the adjustment applied to the *p*-values and the threshold to which those *p*-values are compared.

As is common in statistics, some methods require additional assumptions and the claimed properties are only valid when those assumptions are met. In multiple testing, an important distinction is between methods that achieve weak control and those that achieve strong control. Weak control means that the method achieves the stated error control only when there are no true positives (i.e., all null hypotheses are true). In contrast, *strong* control means that the method achieves the stated control no matter how many of the null hypotheses are true or false. Only methods that provide a strong (general) control are discussed here. In multiple testing, it is also common to encounter assumptions about the dependence among variables or *p*-values; the assumption of independence among variables is unrealistic for omics data, where variables are often positively correlated.

In the following, we first define metrics to quantify false positives and then briefly present some of the methods that have been proposed to control them, focusing only on the essential concepts. We point the more technical reader to comprehensive reviews of multiple testing methods by Dudoit et al. [105] and more recently by Goeman and Solari [106]. A practical introduction providing illustrative examples with implementation in the R language is available in the book of Bretz et al. [107].

The FDR is a popular extension of the concept of type I error for HDD. Using the notation described in Table 17, FDR is the expected (average) value of *Q*, i.e., *FDR* = *E(Q)*, where *Q* = *V/R* if *R* > 0 and *Q* = 0 if *R* = 0 [107]. *Q* is sometimes also called FDP (false discovery proportion). Since the case *R* = 0 is very uncommon in practical HDD applications, the FDR can be roughly thought of as the proportion of false positives among declared positives (i.e., among rejected tests). Controlling FDR is less stringent than controlling FWER, as FDR control inherently allows for some false positives. The goal of FDR control is to identify as many positive test results as possible, while accepting a relatively low proportion of false discoveries. In practice, common choices for FDR control are 5 or 10%.

**Fig. 15:**
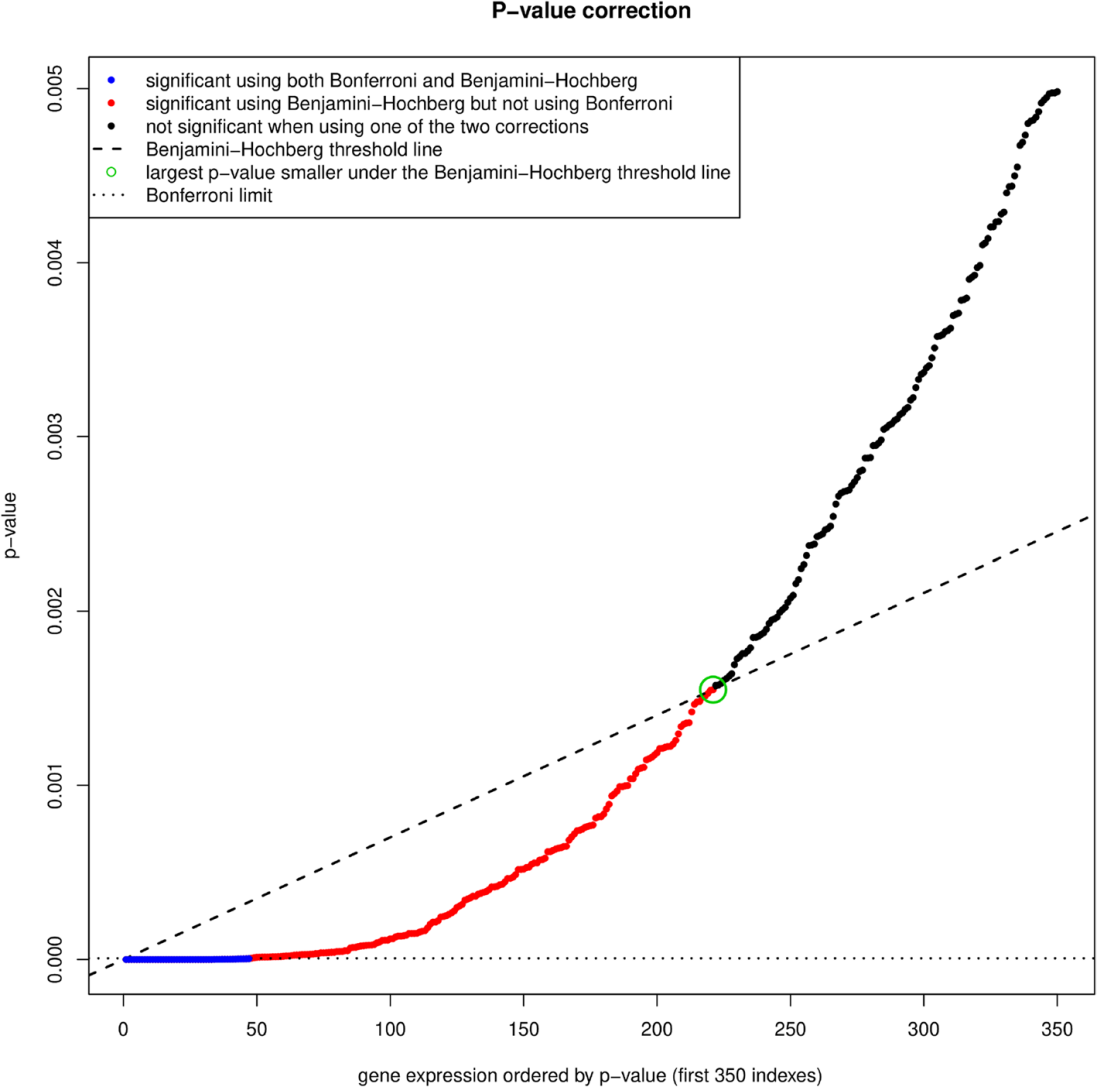
Graphical illustration how the Bonferroni and the Benjamini–Hochberg correction work, for an example with 7129 tests and 0.05 as desired significance level in each case. Applying Bonferroni, only the results of the tests with *p*-values smaller than 0.05 / 7129 (represented by a dotted line) provide evidence against the null hypothesis. For Benjamini-Hochberg, the significant genes are those whose tests yield *p*-values smaller than the largest *p*-value under the threshold, circled in green in the figure. The threshold is represented by the dashed line. The line has intercept 0 and slope 0.05 / 7129, where now 0.05 is the desired level of FDR control

The Benjamini–Hochberg procedure [108] is the most widely used method for controlling the FDR. It is described in Table 19. Notably, the adjusted threshold value used by the Benjamini–Hochberg method is identical to that used by the Bonferroni and Holm methods for the variable with the smallest *p*-value, but it is much larger for the others. It is generally true that lists of discoveries generated by procedures that control the FDR are much longer than those generated by methods that control the FWER at the same level. Yet, like the Bonferroni method, the original FDR method is conservative, effectively controlling the FDR at level *α*·*m*_0_/*m* ≤ *α* if the variables are independent. Many methods were proposed to improve the power of FDR by estimating this unknown proportion of true null hypotheses (*m*_0_/*m*) from data and using it to adapt the threshold value (see [100]). The original FDR [108], which was proposed for independent variables but proven to be valid under the assumption of a positive correlation of the *p*-values, was extended by Benjamini and Yekutieli [109] to handle more general dependencies. This more general procedure has lower thresholds and is more conservative. Several other methods were proposed to control FDR and some error rates closely related to FDR were defined [110]. Figure 15 illustrates how the Bonferroni and the Benjamini–Hochberg correction work.

**Table 19:** Methods for multiple testing corrections controlling the FDR: Benjamini-Hochberg, *q*-values

**Benjamini-Hochberg (BH)**
The Benjamini–Hochberg procedure [108] to control the FDR specifies that the *i*th ordered (smallest to largest) unadjusted *p*-value is compared to the threshold ($\alpha$*/m)·i*, where *i* is the ranking of the *p*-value, *m* is the total number of tests, and $\alpha$ is the desired level of FDR control. Then the largest *p*-value that is smaller than its threshold is identified, and the corresponding test and all tests with a smaller *p*-value are considered significant. Alternatively, one can convert the unadjusted *p*-values to FDR-adjusted *p*-values where the adjusted *p*-value associated with a variable represents the smallest value of FDR at which the procedure would have rejected the test associated with that variable. The intuition behind this correction is linked to the fact that the *p*-values of null variables for independent tests are uniformly distributed; therefore, the ranked *p*-values should lie approximately on the line *y* = *i/m*. In the presence of true positive variables (non-null hypotheses), one would expect a higher concentration of small *p*-values, therefore an excess of *p*-values falling below the line *i*/*m* for lower ranks
***q*-values**
Adjusted *p*-values can also be calculated for FDR-controlling procedures. For a particular variable, the FDR-adjusted *p*-value is sometimes called a *q*-value and can be interpreted as the expected proportion of false positives among all variables with test statistics as or more extreme (with smaller adjusted *p*-values) as the observed value for the variable under examination [110]. Thus, the *q*-value estimates the FDR that would be obtained if this specific *p*-value would be used as the upper threshold for the inclusion of the variables in the list of discoveries. Therefore, *q*-values do not have an obvious interpretation at the level of a single hypothesis. A related limitation is that the interpretation of the FDR results should be restricted to the complete list of discoveries obtained from the analysis, as the properties of subsets with respect to what number or proportion of false discoveries they might contain are not well defined

Many extensions and modifications of the FDR have been proposed. The most common criticism of FDR is that it controls only the *average* proportion of false positives, which might be very variable: in practice, the actual proportion Q of false positives derived from an analysis might differ substantially from the targeted FDR threshold, but the FDR methods do not provide an estimate of this variability. Readers are referred to Goeman and Solari [106] for discussion of methods that aim to control the false discovery proportion with a specified confidence. Other methods have been proposed for control of *local* FDR, a concept that allows a more powerful interpretation of *q*-values at the level of single hypothesis and not as a property of a list of variables [111]. In practice, the FDR-controlling approaches are some of the most widely used methods for multiple testing for omics data, despite some recognized limitations.

##### TEST2.3: Sample size considerations

Determination of an appropriate sample size for a study that will involve conducting an extremely large number of statistical tests is very challenging. Sample size methods must be tailored to the desired approach and criteria for error control. Both false positive (type I error) and false negatives (type II) errors need to be considered. Early in the emergence of omics data, sample size methods focused on FDR control [112], particularly for microarray technology [113]. A recent review mainly focusing on sequencing experiments also provides useful guidance [114].

#### TEST3: Identify informative groups of variables

The multiple testing problem is less severe when the interest is shifted to groups of variables instead of single variables, as described in the introduction of section “TEST: Identification of informative variables and multiple testing” as example (iii) of the main scenarios. In most cases, the groups are prespecified (e.g., genes belonging to the same biological pathway, genes with the same molecular function, or mutations on the same arm of a chromosome). A variable can belong to more than one group, and often the variables belonging to the same group are positively correlated. This type of analysis has the potential of having greater statistical power and greater between study reproducibility than a variable-by-variable analysis.

The methods tailored for the analysis of groups of variables can be divided into two broad classes [115, 116]: The first class are competitive methods, which attempt to identify which variable groups have a stronger association with the outcome (or phenotype) than the other groups. The second class are self-contained methods, which try to identify which of the variable groups contain at least one variable that is associated to the outcome. Example approaches are described below. The popular gene set enrichment analysis (GSEA) and over-representation analysis (ORA) are mixed approaches, while topGO is a competitive method and the global test a self-contained method. In all cases, FWER or FDR can be controlled using any of the methods already described in section “TEST2: Multiple testing” on multiple testing. When applying multiple tests for groups of variables, multiplicity refers to the multiplicity of these groups, not of individual variables. In order to also examine data from a single patient or a small number of samples in experiments, methods have been developed that score individual samples based on gene sets. Singscore [117] is one such approach. It is a rank-based single sample method that generates scores that are stable across a range of sample sizes (Table 20).

**Table 20:** Methods for multiple testing for groups of variables: Gene set enrichment analysis (GSEA), Over-representation analysis, global test, topGO

**Fig. 16:**
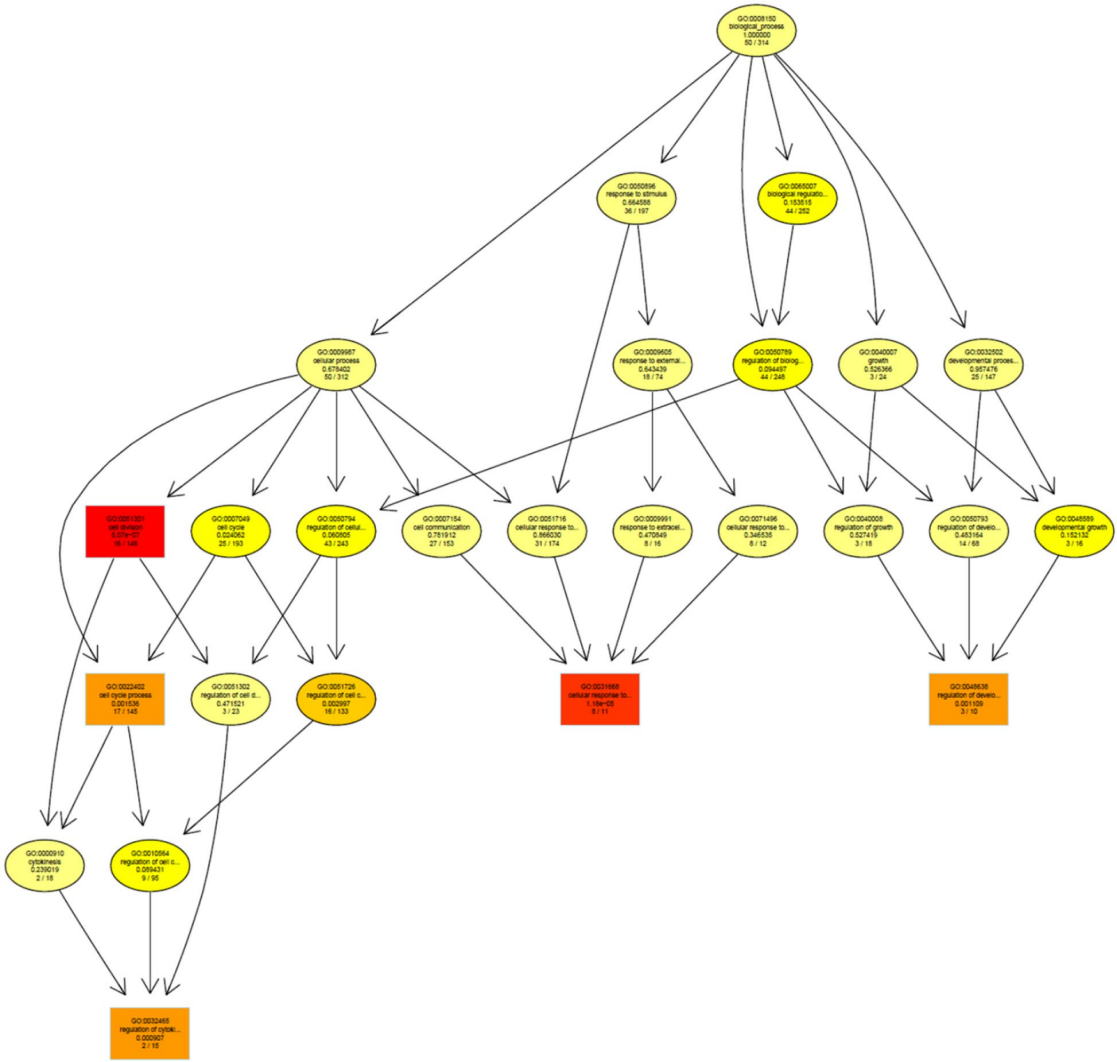
Subgraph of the Gene Ontology (GO) induced by the top 5 GO terms identified by topGO (elim algorithm) for scoring GO terms for enrichment. Rectangles indicate the 5 most significant terms. Rectangle color represents the relative significance, ranging from dark red (most significant) to bright yellow (least significant). The top GO terms are spread across different areas of the GO graph, representing rather different biological processes. Source: [123]

**Gene set enrichment analysis (GSEA)**
The popular gene set enrichment analysis (GSEA; [118]) and its extensions are considered mixed approaches, as they test whether any of the variable groups is associated to the outcome variable and if any of the variable groups is enriched by variables associated to the outcome variable. A summary statistic is computed for each variable, a relative enrichment score based on a signed Kolmogorov–Smirnov statistic is calculated for each group, and its significance is evaluated using permutations. The groups with scores above or below a threshold are called enriched and the false positive rate is evaluated using a permutation procedure that permutes the specimens rather than the variables. Efron and Tibshirani [119] proposed to base the score on a standardized “maxmean” statistic (the standardized maximum of positive and negative summary statistics in each group), thus improving the power of the method
**Over-representation analysis**
Over-representation analysis (ORA; [120]) uses a similar concept to GSEA. It determines which variable groups are more present (overrepresented) in a subset of a given list of “interesting” variables than would be expected by chance. This can also be applied to situations where GSEA is used, but then instead of the Kolmogorov–Smirnov statistic the hypergeometric distribution is used for determining the significance of the over-representation, and thus a subjective cutoff for the summary statistic must be chosen a priori
**Global test**
The global test [121] is based on the estimation of a regression model where all the variables belonging to the group are included as covariates, and the global null hypothesis is tested whether any of the variables is associated with the outcome variable. The method is particularly good at identifying groups containing many variables, each of which might have relatively small effects
**topGO**
The topGO algorithm [122] provides methods for testing specific gene groups defined via the Gene Ontology (GO). The Gene Ontology is a widely recognized comprehensive reference for gene annotations. It assigns genes to GO terms belonging to the three main domains: biological processes, molecular functions, or cellular components. The corresponding gene groups (defined according to GO terms) are widely used prespecified groups of variables, often referred to as gene sets. However, when scoring the relevance of GO terms with methods as mentioned above, due to the high redundancy of many terms resulting in many similar groups of variables, the list of the most significant groups is also highly redundant. topGO provides algorithms for testing GO terms while accounting for the relationships between the corresponding gene groups. As a result, the final list of the most significant groups better represents the diversity of all significant groups, see Figure 16 [123] for the result of the topGO algorithm

### PRED: Prediction

It is often of interest to build a prediction model that takes so-called “predictor variables” (sometimes also referred to as “independent variables”) as input and returns a prediction for a target variable of interest (sometimes also referred to as “dependent variable”) as output. This target variable, which refers either to the present state of the patient or to the future, may be a (binary or multi-categorical) class membership (e.g., treatment responder versus non-responder), a continuous variable (e.g., blood pressure or tumor size after therapy), an ordinal variable (e.g., WHO tumor grade), or a time-to-event (e.g., the overall survival time). Statistically more challenging cases of target variables that are not discussed in this paper are zero-inflated variables (typically continuous with additional frequent 0 values), continuous bounded variables (e.g., with values in [0,1]), or time-to-event variables in the presence of competing risks.

In the HDD setting, the number of candidate variables available to build the prediction model may be very large. This property has implications for construction of prediction models (section “PRED1: Construct prediction models”) and assessment and validation of their performance (section “PRED2: Assess performance and validate prediction models”). Detailed guidance for training, testing, and validation of HDD prediction models is provided by the IOM (Institute of Medicine of the National Academy of Sciences, USA) within a report that identifies best practices for the development, evaluation, and translation of omics-based tests into clinical practice [124]. However, that report does not contain detailed guidance on statistical approaches for construction of prediction models and assessment of their performance. In addition, statistical methodology has seen substantial developments during the last decade.

Many methods to assess model performance and validate prediction models have been developed for low-dimensional data and then adapted to HDD, so a good starting reference is the explanation and elaboration paper of the TRIPOD (Transparent Reporting of a multivariable prediction model for Individual Prognosis Or Diagnosis) reporting guideline [125]. This section explains, expands, and elaborates on existing guidance to more comprehensively cover issues in prediction modelling with HDD.

#### PRED1: Construct prediction models

Researchers developing a prediction model primarily focus on how well the model predicts the outcome of interest, especially for new observations, e.g., for patients whose data were not used to build the prediction model. While this is the main concern, often the researchers are also interested in the interpretation of the model, for example identifying which variables contribute most to the prediction and in what way. From this perspective, models involving only a limited number of predictor variables (denoted as “sparse models”), which clearly distinguish informative variables from non-informative variables, may be preferred to models making use of all variables measured for all observations. This is a particularly big challenge in the HDD setting, where many candidate variables are available. Beyond the issue of interpretability, sparse models may be easier to apply in clinical practice, because fewer variables have to be measured or determined to use them than for non-sparse models. In the case of gene expression, for example, the measurement of, say, 10 genes can be easily performed in any lab using PCR techniques, while the measurement of genome-wide expression requires the use of high-throughput methods (see, e.g., [126]).

A model is said to be “complex” if it reflects many patterns present in the available data, for example, by considering many predictor variables or capturing non-linear effects. Overly complex models risk *overfitting* the data, i.e., adhere too specifically to the data at hand and identify spurious patterns randomly present in the data used for model development that will not be present in independent data (see, e.g., [127]). An overfitted model usually exhibits suboptimal prediction performance when subjected to appropriate unbiased evaluation methods, and interpreting such models can be misleading. In contrast, a model that is not complex enough underfits the data. It misses important patterns that might have been useful for the purpose of prediction. When fitting prediction models, in particular (but not only) in the HDD setting, the challenge thus is to identify the optimal level of model complexity that will yield interpretable models with good prediction performance on independent data (see, e.g., [128, 129]).

The most straightforward statistical approach to construct a prediction model using several predictor variables simultaneously while taking into account their correlation is fitting a multivariable (generalized) regression model, for example a simple linear regression model in the case of an approximately normally distributed target variable. In linear regression, the regression coefficients are fitted such that the sum (for the *n* observations) of squared errors (i.e., of squared differences between the true value of the target variable and the predicted value) is minimal. Mathematically, this basic linear regression amounts to solving a system of *n* equations with *p* + *1* unknowns, where *p* stands for the number of predictor variables. Such a regression model, however, cannot be fitted if the number *p* + *1* of coefficients to fit (the intercept and one coefficient for each variable) exceeds the dataset size *n*. This dimension problem is complicated by the frequently occurring situation in which some of the *p* variables are highly correlated, i.e., they provide similar information. These correlations can cause instability with regard to which variables are deemed important contributors to the model and, thus, can influence model interpretability and performance.

Because the number of predictor variables *p* is usually larger than the number of patients *n* in HDD settings, basic regression models cannot be fitted directly. In this section, we briefly review some key strategies to deal with the dimension problem: variable selection, dimension reduction, statistical modelling (mainly through regularization methods), and algorithmic approaches (at the interface between statistics and machine learning). First, however, we discuss a preliminary step, variable transformation, that can be particularly helpful in the context of HDD analyses.

##### PRED1.1: Variable transformations

As mentioned in section “IDA3: Preprocessing the data,” data may be transformed to obtain certain distributional properties required for the methods that might be used in preprocessing or in downstream analyses of the preprocessed data. For example, (approximate) normal distributions for errors are a prerequisite for the application of tests such as the *t*-test or methods based on linear models such as ANOVA and linear regression [130]. Transformations may also be helpful to dampen the influence of peculiar or extreme observations and may put variables on scales that are more amenable to analysis. For example, one could transform a bounded variable to an unbounded range or convert multiplicative effects to additive effects. It is often preferable to apply suitable transformations first and then work with transformed variables (Table 21).

**Table 21:** Methods for variable transformations: Log-transform, standardization

**Log-transform**
Variables with nonnegative values are frequently encountered in practice and typically have a right-skewed distribution. A logarithmic transformation may be helpful to make the distribution of the data more symmetric. In principle, instead of X, the derived variable log(X) is used as input for prediction modelling [131]. An example in a high-dimensional context is gene expression microarray data, which typically enter in a prediction model after being log2 transformed (see, e.g., [43]). Other transformations than the logarithmic one are, of course, also possible, but rarer
**Standardization**
Another variable transformation often performed in high-dimensional contexts is standardization. Here, the variable is centered (for each value of the variable the mean of the variable is subtracted) and scaled (each centered value is divided by the standard deviation of the variable). This procedure has advantages from an interpretation point of view. For example, the intercept of a linear model including age would represent a person of average age instead of a hypothetical person of age 0. Further, standardization is crucial for the correct implementation of many regularized methods (e.g., lasso and ridge regression, see section “PRED1.4: Statistical modelling”). Note that standardization can cause problems when applying a prediction model to a new dataset. In this case, one either has to use the correction factors calculated from the original dataset or re-compute them on the new dataset, which is problematic because then individual predictions depend on other observations that happened to be included in the new dataset. Standardization is not mutually exclusive with other transformations, e.g., the logarithmic transformation described above, thus it is often performed in addition (i.e., after the logarithmic transformation)

Note that centering and scaling were discussed in section “IDA3.1: Background subtraction and normalization” (referring to normalization), but there the transformation was applied to all values of an observation (a subject, e.g., a patient) to adjust for potential systematic effects and make different observations more comparable, whereas here the transformation is related to all values of a variable.

##### PRED1.2: Variable selection

Variable selection refers to identification of a subset of predictor variables from all available variables, for purposes of building a prediction model. Note that terms as variable selection, selection strategy, or stepwise procedures are often used in the statistical literature, whereas use of the terms feature selection, wrapper, and filter is more common in the machine learning community. Multiple strategies have been proposed in the statistical and machine learning areas; for recent reviews, see, e.g., Heinze et al. [132] and Singh et al. [133]. If available, subject matter knowledge should be included in the variable selection process. In many cases, however, variable selection is performed in a data-driven way, either with filter methods or with wrapper methods. In filter methods, the candidate predictor variables are considered successively independently of each other. Those satisfying a given criterion (for example, those associated with the target variable or those showing sufficient variability across all patients) are selected, while the others are ignored in the remaining analyses (for a comparison of filter methods in classification tasks with HDD data, see [134]). In contrast, wrapper methods select a subset of variables that, taken in combination, yield good performance accuracy (when used for prediction modelling with a considered method). The performance is assessed, e.g., through cross-validation (see section “PRED2.2: Internal and external validation”). Note that an embedded variable selection is also performed intrinsically with model building methods such as lasso and boosting (see section “PRED1.4: Statistical modelling”).

When the variable selection process uses outcome data, care must be taken to avoid optimistic bias in apparent model performance estimates due to multiple testing issues such as those described in section “TEST: Identification of informative variables and multiple testing.” It is critical that any data-driven variable selection steps are included as part of the model building process when model performance is assessed using any internal validation method, see Sachs and McShane [135] for a discussion of the use of “incomplete” cross-validation approaches and the bias inherent in such flawed approaches. Section “PRED2.2: Internal and external validation” provides a further discussion. With an emphasis on LDD, the topic group TG2 “Selection of variables and functional forms in multivariable analysis” of the STRATOS initiative raised several issues needing more research about the properties of variable selection procedures. Authors stressed that it is not straightforward which variable selection approach to use under which circumstances [136]. Obviously, problems mentioned are strengthened in HDD.

##### PRED1.3: Dimension reduction

Data reduction has many purposes, including easier data handling (see also sections “IDA2.4: Graphical displays” and “EDA2.1: Cluster analysis” for aspects regarding data reduction). Concerning prediction, data reduction can help to reduce redundant information that may lead to instability of prediction models, as noted at the beginning of section “PRED1: Construct prediction models.” Data reduction may also facilitate explanation and interpretation by reducing the number of variables to consider. Note, however, that it may yield variables without a meaningful interpretation from a medical point of view [137].

In contrast to variable selection, the idea of *dimension reduction* is not to select variables but to build (a small number of) new variables, often called *components*, that summarize the information contained in the original variables. They can then be used as predictor variables for model building—possibly with a low-dimensional method. However, portability and feasibility of models generated using dimension reduction versus variable selection can be substantially different. To predict outcome using a model containing only a few selected variables, it is sufficient to measure these selected variables, while a model including derived components may require the measurement of all original variables. Consider, for example, deriving a prediction model from gene expression data generated using a microarray that measures 20,000 genes. There is a huge practical difference between using a model requiring input of expression levels of only 10 selected individual genes compared to using a model requiring input of 10 combination scores (components), each of which potentially requires knowledge of expression levels for all 20,000 genes.

The most well-known and widely used dimension reduction approaches are principal component analysis (PCA, see also section “IDA3: Preprocessing the data” for a description) and partial least squares (PLS), where the components are defined as linear combinations of the original variables [138]. While PCA constructs components that have maximal variance and thus capture the signals of all types contained in the data, PLS constructs new variables that have maximal covariance with the target variable of interest. PLS is said to be a supervised method, where the term “supervised” refers to the fact that the target variable determines the construction of the components. Note that dimension reduction can be combined with variable selection.

For HDD analysis, a knowledge-based data reduction may also be useful. There is often external knowledge available about the entities to be investigated, such as knowledge of signaling pathways when analyzing gene expression data, or knowledge on conserved regions when analyzing DNA sequencing data (see also section “TEST3: Identify informative groups of variables” for incorporating information about functional relationships between genes in multiple testing). Attempts to re-discover such knowledge from the data at hand when performing data reduction then will typically be less reliable compared to using a data reduction strategy that explicitly incorporates external information, even if the latter itself also is to some extent unreliable (Table 22).

**Table 22:** Method for dimension reduction: Supervised principal components

**Supervised principal components (SuperPC)**
PCA is conducted based on a subset of preliminarily selected variables. In SuperPC [55], first a variable selection method (see above) is used to reduce the number of prediction variables. This means that the additional step in comparison with PCA is that the subset of predictors selected is based on their association with an outcome, explaining the name supervised. Then, a classical PCA is performed on the reduced space (i.e., only considering the selected variables). The newly constructed components are then used for prediction

##### PRED1.4: Statistical modelling

Several modifications of traditional regression methods are available to address common challenges encountered in HDD settings with *p* > *n*. There is no unique mathematical solution for the standard regression parameter estimates. Traditional regression aims to find the parameters that minimize a sum of squared errors, which can be viewed as minimizing a type of “loss function.” Various modifications to this loss function can be made to permit a unique solution for the regression parameters in the HDD setting. The modifications described in this section impose mathematical constraints on regression coefficients. These constraints effectively limit the number of predictor variables included in the model or the magnitudes of their effects or both. Estimates obtained with such constraints are often referred to as “shrunken.” Some of these constraints can be shown equivalent to adjusting the covariance matrix (e.g., ridge regression; see [139]), but a variety of other constraints can be applied through specification of different loss functions; lasso [140] and elastic net [141] are two examples. Other methods, such as boosting [142], iteratively fit regression models that minimize a specified loss function at each stage. These various approaches usually lead to different models, each of which is optimal according to its corresponding criteria.

Numerous modifications of these basic approaches have been developed in the literature (especially for lasso, due to its variable selection property). Goals can be to recover desirable mathematical properties (e.g., the adaptive lasso [143] uses adaptive weights for penalizing different coefficients and estimates the correct model under some constraints) or to adapt the lasso to specific problems (e.g., the group lasso [144] allows predefined groups of variables to jointly be selected or not) (Table 23).

**Table 23:** Methods for statistical modelling with constraints on regression coefficients: Ridge regression, lasso regression, elastic net, boosting

**Ridge regression, lasso regression, and the elastic net**
Two of the most commonly used constrained regression methods are ridge regression and lasso. Interestingly, the problem of minimization of a loss function under particular constraints can be mathematically rewritten as the minimization of the same loss function with an additional penalty term. Consequently, ridge regression estimates the regression coefficients by minimizing the negative log-likelihood (in linear regression this corresponds to the sum of squared errors) plus a penalty term defined as the sum of the squared values of the coefficients. For lasso, the penalty term is instead the sum of absolute values of the coefficients. In both cases, the amount of penalty to be added is controlled by a tuning parameter, which must be chosen either by the user or as part of the algorithm (usually by cross-validation)
A nice property of the lasso penalty is that it forces many regression coefficients to be 0, providing implicit variable selection (those predictor variables whose coefficients are estimated equal to 0 are removed from the model). However, the lasso has more difficulties in handling correlations among prediction variables. To try to take advantage of the strengths of both methods, a solution that combines both penalties has been proposed under the name of elastic net [141]. A further tuning parameter (in addition to the one that controls the strength of penalty) must be chosen, to define the balance between the two types of penalty. For extreme values of this parameter, namely 0 and 1, elastic net reduces to ridge regression and lasso, respectively
**Boosting**
An alternative to adding constraints to solve the dimensionality problem for HDD is to pursue a stagewise approach. Starting from the simplest model (e.g., in regression, the null model), a single new predictor variable is added stepwise to the model, gradually improving it [142, 145]. The basic idea of boosting (combine several partial improvements to obtain a final good model) works particularly well when the improvements are small. Therefore, at each step, a regularized approach to the univariate problem is performed. For example, in a regression problem, rather than allowing only a single opportunity to add each predictor variable and produce its coefficient estimate, boosting allows a regression coefficient to be updated several times. At each step, the method selects the variable whose regression coefficient is to be updated, based on the minimization of the loss function
Valuable properties already mentioned for lasso, such as shrinkage and intrinsic variable selection, are also achieved by boosting. Shrinkage results from the use of a loss function incorporating a penalty to constrain parameter estimates. The stagewise nature of the procedure potentially allows for stopping before all predictors have been added to the model, effectively setting the regression coefficients for the remaining predictor variables to zero. When to stop updating the model to avoid excessive complexity and, consequently, overfitting is a crucial decision for which several criteria have been proposed, see, e.g., Mayr et al. [146]

##### PRED1.5: Algorithms

Boosting can be seen both as a statistical method, when a statistical model is fitted, and as an algorithmic approach, when it is implemented as a black box. In the latter case, the prediction updates are unrelated to an underlying statistical model, and only aim at minimizing a loss function [147]. Several machine learning algorithms have been developed to provide prediction rules [148]. The prediction model is constructed without variable selection or dimension reduction as a preliminary step, in a fully data-driven way, i.e., (in contrast to statistical methods) without assuming a particular model for the dependence between target and predictor variables. These algorithmic approaches may allow more flexibility to handle aspects such as non-linear or interaction effects, but often they are also less interpretable.

Machine learning algorithms comprise a diverse collection of methods. They include, among others, methods based on consideration of nearest neighbors in the predictor space (such as kNN), decision trees for classification and for regression (tree-based methods based on recursive partitioning of the predictor space), random forests (ensembles of decision trees, i.e., sets of decision trees whose predictions are averaged), and more complex approaches such as deep learning (neural networks with different structures and typically a huge number of parameters). In the HDD setting, many of these machine learning methods have been successfully used, but one must be particularly careful if the methods require the estimation of a large number of parameters, which applies especially to deep learning. Here, the overfitting problem discussed above becomes even more severe. Unbeknownst to users, some software developed to implement complex algorithms could have faulty designs that result in incorrect or overfitting results; hence, algorithms must be carefully tested [149] (Table 24).

**Table 24:** Methods for statistical modelling with machine learning algorithms: Support vector machine, trees, random forests, neural networks and deep learning

**Support vector machine (SVM)**
A support vector machine (SVM) is a typical example of an algorithmic method developed in the machine learning context [150]. It is mostly used for classification, i.e., to predict the response class of the observations (e.g., healthy vs. sick patients), but can also be applied for regression. An SVM divides a set of observations into classes in such a way that the widest possible area around the class boundaries remains free of observations; it is a so-called Large Margin Classifier. The main idea is to construct a *p-1* dimensional hyperplane (imagine a two-dimensional plane in a three-dimensional space, or a straight line in a plane) which separates the observations based on their response class. Often it is unrealistic to find such a perfectly separating hyperplane and one should accept some misclassified observations. Therefore, in the standard extended version of an SVM, observations on the wrong side of the boundaries are allowed, but their number and their combined distance to the boundary are restricted, such that a tuning parameter, usually denoted by C, defines how much “misclassification” is allowed. In addition, the extended implementation of kernel-based methods allows non-linear separating boundaries.
**Trees and random forests**
One of the simplest algorithmic tools for prediction is a tree, in which the prediction is based on binary splits on the variable space. For example, a simple tree could have two nodes (splits): a root (the first split), which divides the space into two regions based on the presence of a genetic mutation, and a second node that divides the observations with this mutation again into two parts, based on another mutation. A tree can be grown further, until a predetermined (usually via cross-validation) number of regions in the variable space is reached [151]. In many studies, variables are measured on different scales (binary, ordinal, categorical, continuous) and several binary splits are possible, raising the issue of multiple testing. Algorithms which do not correct for multiple testing are biased in favor of variables allowing several cut points over binary variables [152].
Simple trees are often unstable, i.e., fitting a tree to subsets of the data leads to very different estimated trees. One idea to solve this problem is to aggregate the results of trees computed on several bootstrap samples (bagging = Bootstrap AGGregatING, [153]). For example, for continuous variables, the predictions of different trees are typically averaged, and for categorical variables, for each category, the proportion of trees with this category as prediction is used as estimate of the probability of that category.
While bagging partially mitigates the instability problem, often it is not very effective, due to the strong correlation among the trees. Random forests [154] improve upon this approach by limiting the correlation among the trees through use of only a subset of the variables in the construction of each tree. As in bagging, the results of the different trees are then aggregated to obtain a final prediction rule. Tuning parameters such as the size of the subset and the number of bootstrap samples must be chosen, but often default values are successfully used. While using the default values is often a good strategy in the LDD case, this is not necessarily the case for HDD problems. For example, the best size of the variable subset depends on the dimension of the total number of variables available [155]. An overview from early development to recent advances of random forests was provided by Fawagreh et al. [156].
**Neural networks and deep learning**
In recent years, machine learning techniques like neural networks and deep learning have gained much interest due to their excellent performance in image recognition, speech recognition, and natural language processing [157, 158]. They are based on variable transformations: in neural networks, the predictor variables are transformed in a generally non-linear fashion through what is called an *activation function*. One popular choice for the activation function is a sigmoid or logistic function, which is applied to a linear combination of predictor variables (the coefficients used in the linear function, which provide the individual contribution of each predictor variable, are called *weights*). These new transformed variables (*neurons* in machine learning terminology*, latent variables* in statistical terms) form the so-called hidden layers, which are used to build the predictor. Mathematical theorems show that increasing the number of hidden layers and decreasing the number of neurons in each layer can improve the prediction performance of neural networks.
Specific neural networks with many hidden layers are called deep learning. The choice of the tuning parameters (activation function, number of hidden layers, and number of neurons per layer) characterizes the different kinds of neural networks (and deep learning algorithms). In the high-dimensional contexts, special approaches (e.g., selecting variables or setting weights to zero) are used to avoid overfitting.
Deep learning methods are extremely successful in the situation of a very large number of observations (as in image classification and speech recognition based on huge databases). However, they tend to generate overfitted models for typical biomedical applications in which the number of observations (e.g., number of patients or subjects) does not exceed a few hundred or thousand (see Miotto et al. [159] for a discussion of opportunities and challenges).

##### PRED1.6: Integrating multiple sources of information

A major challenge for HDD, both for omics data and for electronic health records, is the integrative analysis of different data types. For instance, multiple types of omics data including proteomic, transcriptomic, and genomic, may be measured on the same subject. For health records data, various variable types are combined, such as blood values, urine values, cardiography measurements (ECG or EKG), categorical diagnostic measurements, or a variety of demographic variables. This has implications for visualization and use of clustering methods, which are often designed for a single data type. Conducting and interpreting joint analyses of disparate variable types can be challenging. Richardson and coauthors [160] distinguish between “horizontal integration” applied to the same type of data across multiple studies and “vertical integration” applied to different types of data on the same sample of subjects. The distinction between horizontal and vertical refers to the fact that, usually, data from high-throughput experiments are organized with samples represented by columns and variables by rows.

Regarding horizontal integration, the meta-analytic approach of pooling summary measures of association is the most used approach. For other applications, such as clustering, in order to deal with different normalizations and platforms for the different datasets, centering, and standardization [161] or specific methods should be considered; for clustering, see for example Huo et al. [162]. Vertical data integration is typically model-based and the model used considers the specific characteristics of the data to be integrated and of the research question (whether exploratory or predictive).

In biomedicine, integration of multiple omics data types can provide deeper biological insights compared to individual omics in terms of disease subtyping, biomarker identification, and understanding of molecular mechanisms in diseases. For example, two different tissues from the same or different organism may carry an identical DNA sequence for a particular gene, but the gene may be inactivated by methylation in one of the tissues and not in the other; or the aberrant expression of one gene regulating the function of another downstream in the same biological pathway might be evident by observing the altered expression of the downstream gene at the RNA or protein level.

Richardson and coauthors [160] reviewed some vertical integrative analysis approaches, including integrative clustering and regression. The integrative clustering approach of Shen and coauthors [163], called iCluster, involves projection, via regression modelling, of the data onto scores representing a set of latent biological subtypes assumed common across data types. Resulting predicted biological subtype scores are clustered to identify latent subtype membership, and estimated coefficients from the fitted regression models can provide insights into data features that associate with certain subtypes. Mo and coauthors subsequently developed iCluster + to allow for other non-continuous, non-Gaussian data types [164]. More complex Bayesian mixture modelling approaches have also been developed to offer greater flexibility to accommodate mixed data types (e.g., discrete mutation indicators in combination with continuous RNA or protein expression measures), provide metrics reflecting uncertainty about estimated underlying structure, and allow for elucidation of potentially different structure from different data types [165–168]. Integrative regression techniques are useful for supervised analyses of integrated data types, such as building a regression model for prediction of an outcome or phenotype. These methods allow to utilize structure inherent in different data types (e.g., DNA sequence location, functional categories of proteins, metabolic or signaling pathways) to effectively reduce the high dimensionality of the predictor variable space to facilitate development of more parsimonious and interpretable models relating the multi-omics data to outcomes or phenotypes of interest. Multi-omics integration methods using autoencodeurs in a deep learning setting are reviewed by Benkirane and coauthors [169]. For more details, readers are referred to Richardson and coauthors [160] and references therein.

Although many prediction models for clinical outcomes have been developed based either on clinical data or (more recently) on high-throughput molecular data (e.g., omics), far fewer models have been developed to incorporate both data types through vertical integration. The paucity of such models in the literature and in clinical use persists despite suggestions that a suitable combination of clinical and molecular information might lead to models with better predictive abilities (e.g., [170, 171]).

In many medical specialties, there are some widely available and accepted clinical predictors with predictive value already validated in several independent populations. Strategies to combine such established clinical predictors with different data types, including high-dimensional omics data, have been proposed [172]; some examples have been published [173, 174], but applications are still rare. Volkmann and coauthors [174] investigated whether better use of the predictive value of clinical data has an influence on the added predictive value of molecular data. This concept can also be extended to multi-omics data [175].

Conceptually, it is obvious that incorporation of important clinical variables can potentially lead to better prediction models; thus, those variables should be considered in combination with molecular data. De Bin et al. [176] present strategies to combine low- and high-dimensional data in a regression prediction model, analyzing the influence of the complex correlation structure within and between the two data sources. In some situations, predictive value of molecular data might be fully captured through the clinical variables, thereby eliminating the need for the molecular data in the prediction model [172].

#### PRED2: Assess performance and validate prediction models

Perhaps even more than constructing predictive models and algorithms, evaluating their performance and validating them are key challenges. For HDD, not only the choice of suitable measures to assess and compare model performance (see section below), but also the way of computing these measures is generally not straightforward.

##### PRED2.1: Choice of performance measures

Prediction performance is typically assessed by comparing the true and the predicted values of the target variable. The comparison is based on specific metrics, mainly depending on the nature of the target variable. Typical metrics include mean squared error or mean absolute error for continuous target variables, area under the curve (AUC) or Brier score for binary target variables, and calibration plot and time-dependent Brier score for time-to-event variables. Such measures can be used to quantify the performance of a model (or algorithm) or to compare different models constructed using the same dataset. In most biomedical applications, the goal of a comparative assessment is to select a final model [177, 178]. Models of absolute risk that depend on covariates have been used to design intervention studies, to counsel patients regarding their risks of disease or future disease-related events, and to inform clinical decisions. Several criteria related to “calibration” and “discriminatory power” have been proposed [179, 180]. Often the main interest will be in the added value of biomarkers or gene signatures relative to an existing clinical prediction model. Several performance measures are available to quantify the added value [181].

For a clinical task, several very different models with equivalent prediction performance may be available. Not only, but especially in this situation, other aspects of the models can play an important role. Particularly noteworthy aspects of a model are sparsity, stability, interpretability, and practical usefulness [7, 182]. Regarding sparsity, when selecting a final model from among several with comparable prediction performance, selection of the most parsimonious (e.g., the model with smallest number of predictor variables) is preferred. Stability refers to the degree to which small changes in the data may produce large changes in the predictor output. A majority of predictors derived from HDD suffer from poor stability, irrespective of the method used to fit them, although some methods are more affected than others (see [183] for an overview of stability measures, and Sauerbrei et al. [184] for stability investigations of regression models for LDD and HDD). For HDD, the stability problem is due to the myriad ways to combine a set of predictor variables to derive similar performing predictors. If the stability is found to be low, then interpretation of specific model components (the list of selected predictor variables, relationships between predictor variables, etc.) should be avoided. In terms of interpretability of the model, strong prior biological knowledge may also be taken into account, similar as for the aim of data reduction described above (Table 25).

**Table 25:** Methods for assessing performance of prediction models: MSE, MAE, ROC curves, AUC, misclassification rate, Brier score, calibration plots, deviance

**Fig. 17:**
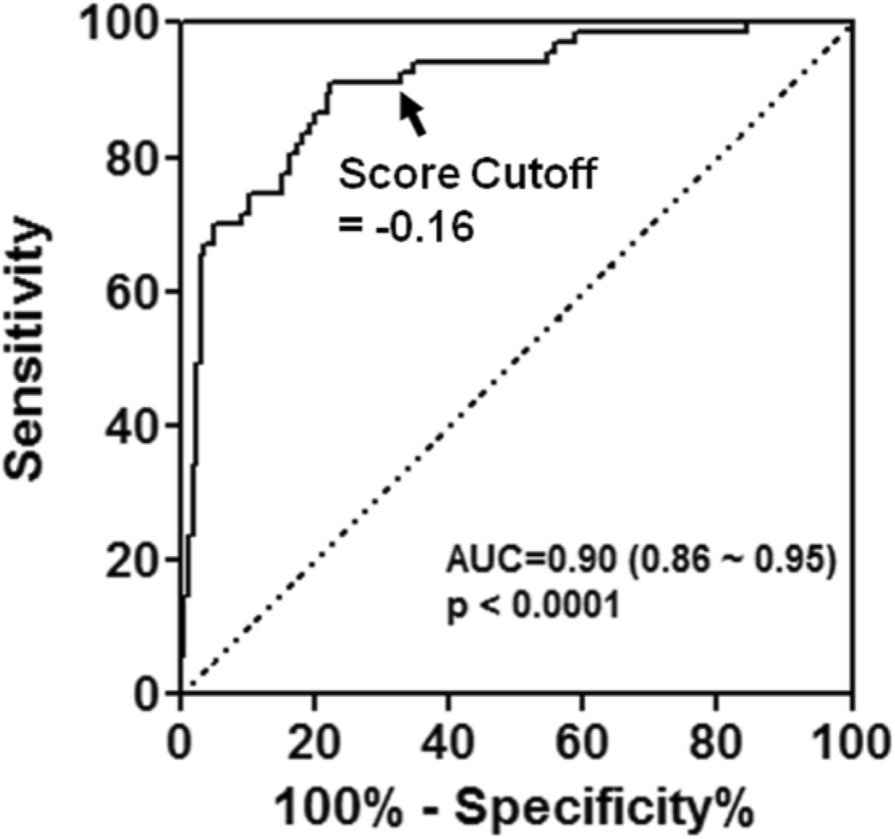
Receiver operating characteristic (ROC) curve that illustrates the predictive performance of a gene signature including 227 genes for the prediction of chemotherapy response in serous ovarian cancer, obtained using the TCGA (The Cancer Genome Atlas) data set. The arrow indicates the sensitivity and specificity values obtained for a selected cutoff value that can serve as a threshold for patient stratification. In this example, the AUC is evaluated on the same data used to train the classifier, so it is likely to be overoptimistic. Source: [185]

**Fig. 18:**
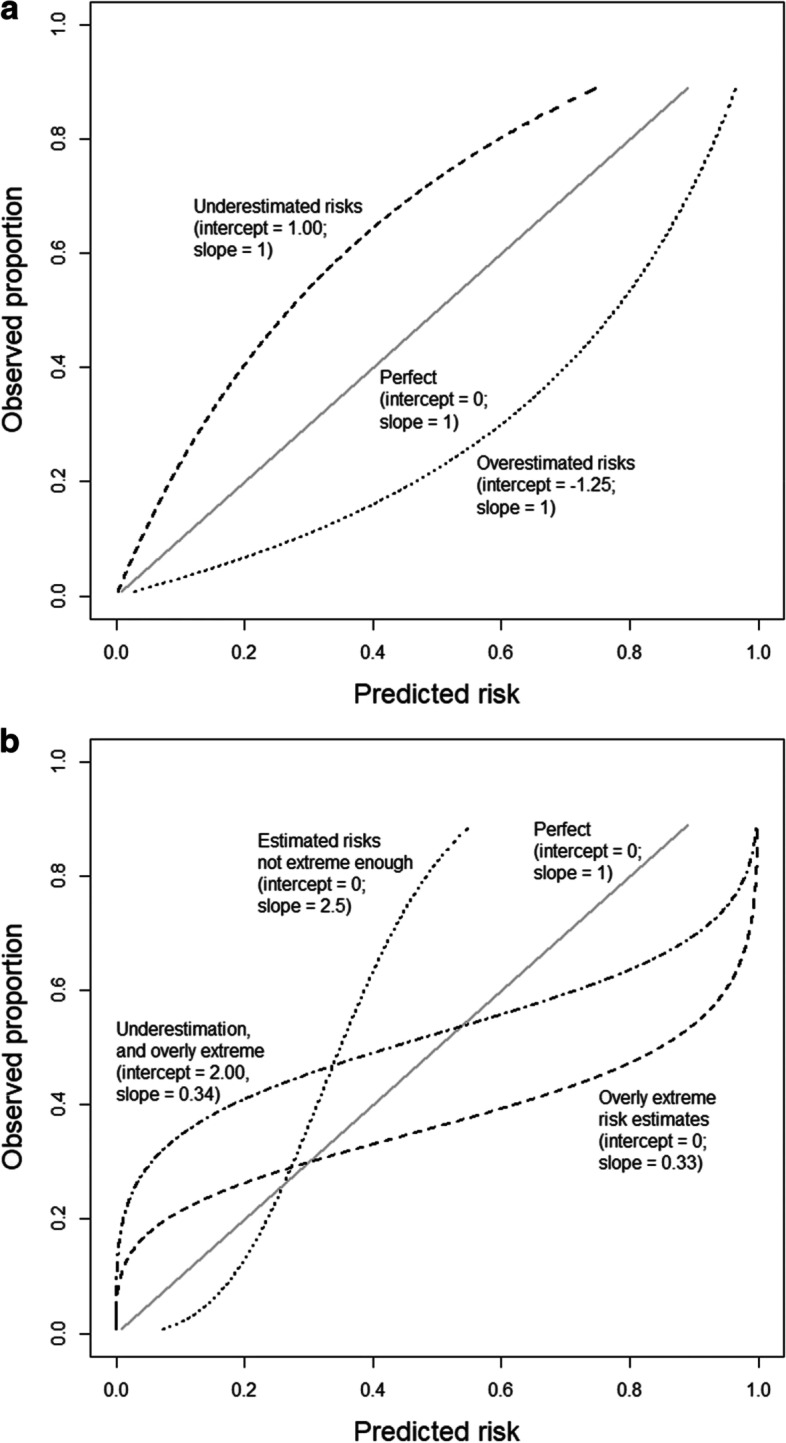
Illustrations of different types of miscalibration, visualized by calibration plots. Illustrations are based on an outcome with a 25% event rate and a model with an area under the ROC curve (AUC or c-statistic) of 0.71. Calibration intercept and slope are indicated for each illustrative curve. **a** General over- or underestimation of predicted risks. **b** Predicted risks that are too extreme or not extreme enough. Source: [187]

**Mean squared error (MSE) and mean absolute error (MAE)**
*Mean squared error* (MSE) and *mean absolute error* (MAE), sometimes denoted as *mean squared prediction error* (MSPE) and *mean absolute prediction error* (MAPE) to emphasize the fact that they are computed on a test set (see discussion below), are commonly used measures to evaluate the prediction performance of a model in the case of a continuous target variable. They are computed by averaging the squared differences or the absolute differences, respectively, between the values predicted by the model and the true values of the target variable. Note that the MSE, being a quadratic measure, is sometimes reported after a square root transformation, the so-called *root mean squared error* (RMSE)
**ROC curves and AUC**
A *receiver operating characteristic* (ROC) curve is a graphical plot that facilitates visualization of the discrimination ability of a binary classification method. Many statistical methods classify observations into two classes based on estimated probabilities of their membership. If the probability is larger than a threshold, then the response is classified as positive (e.g., sick), otherwise as negative (e.g., healthy). This threshold is mostly set to 0.5 or to the prevalence of the positive cases in the dataset. Choosing a lower threshold corresponds to more positive predictions, with the consequence of increasing the percentage of observations correctly classified positive among those actually positive (*sensitivity*) with the potential cost of decreasing the percentage of observations correctly classified negative among those actually negative (*specificity*). Conversely, a larger threshold generally leads to lower sensitivity and higher specificity
The ROC curve is typically constructed with values for 1 − specificity (*x*-axis) plotted against the values for sensitivity (*y*-axis) for all possible values of the threshold. The result is a curve that indicates how well the method discriminates between the two classes. Models with the best discrimination ability will correspond to ROC curves occupying the top left corner of the plot, corresponding to simultaneous high sensitivity and high specificity. A ROC curve close to the diagonal line from lower left to upper right represents poor discrimination ability that is no better than random guessing, e.g., by flipping a coin. The information provided by the ROC curve is often summarized in one single number by calculating the *area under the curve* (AUC). Best classifiers obtain an AUC value close to 1, while methods not better than random guessing exhibit values close to 0.5. Figure 17 [185] shows an exemplary ROC curve corresponding to high discrimination ability with AUC = 0.90 (and confidence interval [0.86, 0.95])
Caution is advised regarding the risk of overestimating the performance of a classifier based solely on the AUC value, as the binary decision depends on an optimized threshold, which can be quite different from 0.5. This problem is especially important for HDD, since there is a lot of flexibility to tune and optimize the classifier, including the decision threshold, based on the large number of predictor variables. Calibration plots (see below) are also important to assess whether the classifier is well calibrated, i.e., estimated probabilities correspond to similar proportions in the data
**Misclassification rate**
A simpler measure of the prediction ability in the case of categorical response is the misclassification rate that quantifies the proportion of observations that have been erroneously classified by the model. Here, in contrast to AUC, smaller values are better. While this measure is simple and can be used even if the classifier does not assign probabilities to observations, but only predicts classes, it does not differentiate between false positives and false negatives. Therefore, the overall misclassification rate can be heavily dependent on the mix of true positive and true negative cases in the test set
**Brier score**
While the misclassification rate only measures accuracy, the Brier score also takes into account the precision of a predictor [180, 186]. The Brier score can be applied for binary, categorical, or time-to-event predictions. It calculates quadratic differences between predicted probabilities and observed outcomes. Thus, it can be considered the counterpart for these prediction targets of the MSE used for regression models. The Brier score is particularly useful because it captures both aspects of a good prediction, namely *calibration* (similarity between the actual and predicted survival time) and *discrimination* (ability to predict the survival times of the observations in the right order). For survival data, the Brier score is generally plotted as a function of the time, where higher curves mean worse models. Alternatively, the area under the Brier score curve is computed, leading to the *integrated Brier score*, which summarizes in a single number the measure of the prediction error (lower being better)
**Calibration plots**
Calibration plots for statistical prediction models can be used to visually check if the predicted probabilities of the response variable agree with the empirical probabilities. For example, for logistic regression models, the predicted probabilities of the target outcome are grouped into intervals and for all observations within each interval the proportion of observations positive for the target outcome are calculated. The means of the predicted values are plotted against the proportion of true responders across the intervals. For survival models, the Kaplan–Meier curve (the observed survival function) can be compared with the average of the predicted survival curves of all observations. Poorly calibrated algorithms can be misleading and potentially harmful for clinical decision-making [187]. Figure 18 [187] visualizes different types of miscalibration using calibration plots
**Deviance**
The deviance measures a distance between two probabilistic models, and it is based on likelihood functions. It can be used to perform model comparison, for any kind of response variable for which a likelihood function can be specified. For a Gaussian response, it corresponds (up to a constant) to the MSE and thus provides a measure of *goodness-of-fit* of the model compared to a null model without predictors. For model comparison, when computed on the training set (see discussion below) to choose the “best” model among several alternatives, it is often regularized. A factor is applied which penalizes larger models (large *p*, where *p* is the number of predictor variables), obtaining measures such as the information criteria AIC (penalty equal to *2p*) and BIC (penalty equal to *p* * log *n*). The specific choice of the information criterion is difficult and depends, e.g., for classification tasks, also on the relative importance of sensitivity and specificity [188]

##### PRED2.2: Internal and external validation

Whatever measure of model performance has been chosen, computing it on the same dataset that was used for constructing the model may lead to a dramatic over-estimation of the performance. Instead, one should assess prediction performance using independent data, i.e., data not used to construct the model [189–191]. One classical procedure is to split the given dataset into a training set and a test set, and then to construct the model using only the training set and evaluate the model using only the test set. This is one type of “internal validation,” in contrast to “external validation,” where data from independent patient cohorts are used [192].

Due to the typical instability of predictors developed using HDD, this sample splitting procedure is very risky, as in most cases the specific split heavily influences the result. Resampling techniques, such as cross-validation, subsampling and bootstrapping, can be less risky, although even those methods cannot avoid impact of biases in the data introduced by faulty designs such as those that would confound batch effects with outcome variables. The common idea behind these procedures is to repeatedly use a part of the dataset as training dataset, i.e., to construct a prediction model, and the other (non-overlapping) part as test dataset to evaluate the constructed model. This process is repeated several times for different splits into training and test data to produce a more stable and representative estimate of model performance.

For such approaches, a bias in the performance estimates must be considered (see also [135]). This bias occurs because the training data sample size is smaller than for the full dataset, and therefore prediction models built on the training dataset tend to have somewhat worse performance than a final model built on the full data. The latter is typically used for further evaluation. This bias becomes larger the smaller the training dataset is compared to the full dataset. This aspect is less relevant if the sample size of the full dataset and thus of the training dataset is very large.

**Fig. 19:**
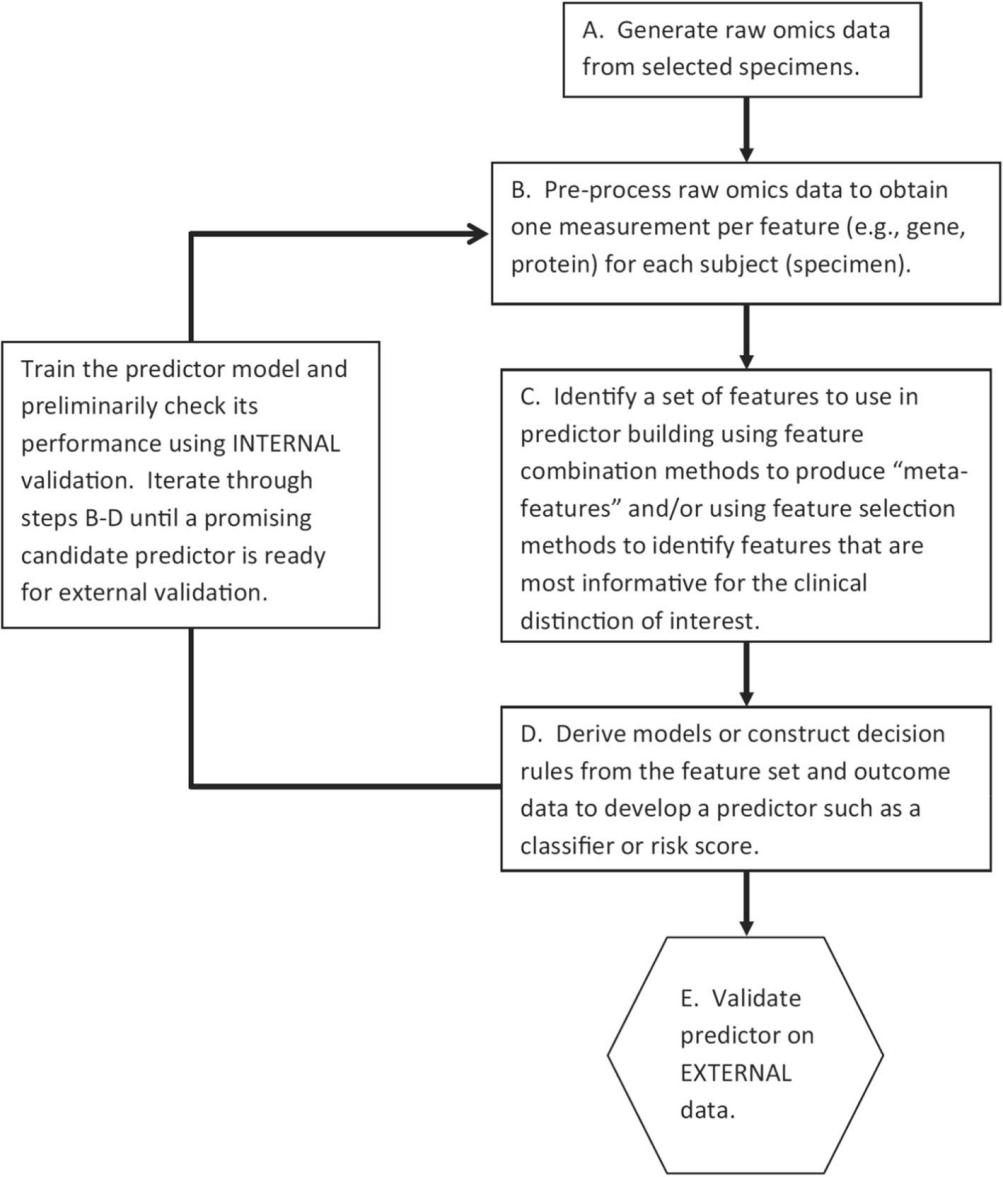
Schematic representation of an appropriate omics predictor development process, with internal validation for improving prediction performance (left box) and external validation for assessing prediction performance on external data. Source: [203]

One misleading practice is use of resampling procedures for multiple different prediction modelling methods or for different parameter values, and then reporting results for only the model with best performance. This practice leads to over-optimism in model performance because it neglects to acknowledge and account for the fact that the reported model was the result of another optimization process [201]. Such studies aiming to find “best” models occur quite frequently in the context of HDD. While it would be naive to expect that investigators will not try multiple approaches to develop a prediction model, the key is transparency in reporting how many models were actually produced and evaluated, and appropriately accounting for the additional selection step. One should either validate the final selected model using an independent dataset (see Table 26), or when such a dataset is not available, embed the selection process in the cross-validation procedure, i.e., perform a so-called nested cross-validation procedure [190, 202]. Figure 19 [203] shows a schematic representation of a suitable process for developing a predictor, here specified for omics data, in which the discussed aspects are adequately taken into account.

**Table 26:** Methods for validation of prediction models: Subsampling, cross-validation, bootstrapping, use of external datasets

**Subsampling**
Subsampling is probably the most straightforward procedure to address the stability issue discussed above. Instead of relying on the result of one single split in training and test sets, the prediction measure is computed for a large number (at least 100) of splits. In practice, for each split, the model (or algorithm) is trained on a part of the data and evaluated on the rest. The results are then averaged to yield a summary measure of performance
**Cross-validation**
Subsampling can substantially improve stability compared to use of a single data split, but a potential criticism is that it does not guarantee (for a finite number of replications) that all observations are used equally frequently in the training set and in the test set. Cross-validation ensures balance by splitting the observations in *K* approximately equal-sized portions (folds) and using, in turn, *K* − 1 folds to build the model and the remaining fold to evaluate its performance. Every single observation is then used *K* − 1 times to train the model and once to test it. The *K* results are then averaged. One drawback for classical cross-validation is that the procedure relies on the specific split in *K* folds. To address this issue, the cross-validation procedure can be repeated several times, combining the idea of cross-validation and subsampling [193]
**Bootstrapping and its modifications**
Similar to subsampling, bootstrapping is based on the idea of generating a large number of training and test sets. In contrast to subsampling, bootstrapping generates training sets of the same size as the original sample, by resampling observations *with replacement* [194, 195]. Since some observations are then used multiple times in the bootstrap-generated training set, other observations are not included at all, and these then form the test set on which the model is evaluated
Bootstrapping is known to overestimate the prediction error, since the training datasets are smaller than the full dataset, as discussed above. Adjustments to the method have been proposed, for example, the 0.632 bootstrap and the 0.632+ bootstrap [196]. Both modifications balance the overestimated bootstrap-based error estimate with the heavily underestimated corresponding error estimate computed on the training set. An overview of many different bootstrapping approaches for practitioners and researchers was provided by [197]
**Use of external datasets (“external validation”)**
While resampling-based approaches can be useful to evaluate and compare performance of prediction models, they do not meet validation standards typically desired in real-world scenarios. Generally, the goal is to develop a prediction model that generalizes well to independent patient cohorts. This refers both to future patients from the same, say, clinical centers as those from which the data used for the construction of the model were obtained, and to patients from different clinical centers [198–200]. Resampling techniques reflect the model performance for independent patient data only if the distribution of the independent data is the same as the original. This assumption can justifiably be questioned when high-dimensional omics or other biomarker data are involved, which may be generated in a new laboratory or according to modified methods, or at very least subject to different batch effects (see section “IDA3.2: Batch correction”). For all of these reasons, validation on external data (cohorts) is essential to have sufficient confidence in the performance of a predictor for clinical use

##### PRED2.3: Identification of influential points

Identification of possible influential observations, defined as those for which inclusion or exclusion in model development might substantially alter characteristics of the final model [204], is an important aspect of prediction modelling that is often neglected in HDD settings, and even frequently in low-dimensional settings. Model alterations can be related to variable selection (see, e.g., [205]), functional forms (e.g., [206]) or parameter estimation (e.g., [207]). Influential points can be outliers in some of the variables (observations suspiciously different from the rest, such that they are probably generated by a different mechanism [208]), but they do not need to be.

For HDD, identification of influential points is particularly difficult, due to data sparsity (the so-called “curse of dimensionality”) and, more generally, the increased difficulty in identifying data patterns, especially by graphical methods. Many available methods for influential point detection are extensions of traditional low-dimensional tools such as Cook’s distance [204] and the CFBETA / DFFITS measures [209]. Examples of adaptations to the high-dimensional framework of the former are Zhao et al. [210] and Wang and Li [211], of the latter Walker and Birch [212] and Rajaratnam et al. [213]. Focusing more on statistical models (see section “PRED1.4: Statistical modelling”), methods like those of Shi and Wang [214] and Hellton et al. [215] investigate the effect of influential points on the choice of the tuning parameters, again adapting existing low-dimensional approaches (the aforementioned DFFITS measure and the resampling approaches in De Bin et al. [205], respectively). The latter is an example of how cross-validation and subsampling can be used to detect influential points by tracking large changes in the estimates when one (or a few) observation is omitted. Although influential points and outliers can strongly affect the results of analyses in HDD settings, systematic checks for them seem to be often ignored in the literature, despite of the availability of various techniques [216, 217].

In a study on the classification of breast cancer subtypes, Segaert et al. [218] stress that classical statistical methods may fail to identify outliers and argue for robust classification methods that flag outliers. They propose the DetectDeviatingCells outlier detection technique. Specifically for HDD, Boulesteix et al. [216] propose a rank discrepancy measure that considers the difference between gene rankings for the original data and for a pretransformation that tries to eliminate the effect of extreme values. For survival data, Carrasquiha et al. [219] propose a rank product test to identify influential observations, and more techniques have been proposed recently. Fan [220] released the R package HighDimOut, which contains three high-dimensional outlier detection algorithms. However, none of the approaches seems to have gained popularity in practice. More guidance and comparisons of available approaches are needed.

##### PRED2.4: Sample size considerations

Recent guidelines for calculating sample size when developing a risk prediction model [16] are not specifically tailored for applications involving variable selection or shrinkage methods (such as LASSO or Ridge Regression). This is the situation in high-dimensional settings, where variable selection or dimension reduction is needed to identify prognostic variables or components. The available methods for sample size planning [17, 18] and references therein are based either on simulations that require assumptions of feature independence or on the availability of a pilot dataset or preliminary data, but these methods are hardly used in practice. Moreover, penalized estimation has often been proposed for situations with potentially large overfitting problems, while recent evidence suggests that it yields unstable results, especially for small sample sizes [221], when overfitting is a major concern.

A practical sample size method for planning a preliminary study of a prognostic biomarker is suggested for microarray technology [113], which can be used in more general settings. When a gene signature is already available from previous exploratory studies, a formal calculation for a predictive model, including the gene signature and standard prognostic covariates, can be performed according to available guidelines, taking also into account the need for external validation [16, 200].

### Good reporting to improve transparency and reproducible research

Reporting of studies involving HDD can be particularly challenging and at the same time especially important due to the many potential pitfalls in the collection and analysis of complex HDD as described herein. Complete and transparent reporting of these studies is critical to allow independent researchers to evaluate how a study was designed, conducted, and analyzed so that quality and relevance of the findings can be judged and interpreted in appropriate context. Provision of data and computer code may be required to achieve full transparency.

Guidelines for reporting of many types of health research have already been developed and are largely applicable in HDD settings. Simera et al. [222] introduced the EQUATOR (Enhancing the QUAlity and Transparency Of health Research) network as an umbrella organization for the reporting of studies in the health sciences. Most relevant for HDD data are the REporting recommendations for tumor MARKer prognostic studies (REMARK) [11] and TRIPOD for the reporting of multivariable prediction models for individual prognosis or diagnosis [12]. For both reporting guidelines, more detailed “explanation and elaboration” papers have been published [125, 223], which also include several sections on statistical analyses. Furthermore, the two-part REMARK profile, a structured display that summarizes key aspects of a study, has been proposed to improve completeness and transparency of reporting, specifically of statistical analyses. The TRIPOD checklist distinguishes between model development and validation. Both guidelines were developed for markers and for models based on clinical data, with no more than a few dozen potential predictors in mind.

In an article stressing the importance of registering diagnostic and prognostic research, Altman [224] clearly expresses that non-reporting and misleading reporting do not just mislead researchers in the field, they also diminish the evidence base underpinning clinical practice and harm patients. To improve on such an unacceptable situation of non-transparency in studies, several initiatives including data pooling, registers, and journal requirements for protocols were started, see Peat et al. [225] for a detailed discussion with an emphasis on prognosis research.

Obviously, reporting of artificial intelligence and machine learning methods come with a large number of additional challenges. Concerns have been raised that they are overhyped in clinical medicine (see, e.g., [226]) and, if not used with proper expertise, have methodological shortcomings, poor transparency, and poor reproducibility [227]. There is a strong need for applications of machine learning techniques to adhere to established methodological standards already defined in prediction model research [228].

## Discussion

In this section, we first summarize the content and the key messages of this overview paper. We also briefly present the relationships of the other topic groups of the STRATOS initiative to the HDD-focused TG9 group and discuss the importance of further collaboration.

Biomedical research has always relied on a combination of observational studies, carefully controlled laboratory experiments, and clinical trials, but the types of data generated and analyzed in these studies continue to evolve and now more often include HDD. The high dimensionality may result from new technologies such as omics assays, which are capable of comprehensive interrogation of biological specimens, or from increased ability to merge data from multiple information systems such as electronic health records or registries. HDD present many new challenges for statistical design and analysis of biomedical research studies. This overview provides a gentle introduction to basic concepts and useful strategies for design and analysis of studies involving HDD. Key points are summarized in the discussion that follows.

Study design for prospectively planned investigations and vigilance to detect (and avoid when possible) confounding in observational studies remain as important for studies involving HDD as for other studies. Consequences of inattention to these aspects can be particularly damaging when HDD are involved. While HDD may provide greater opportunity for discovery of new biological and clinical concepts and associations, they might also be more susceptible to influence of ancillary confounding variables and technical artifacts. Therefore, initial examination of data for technical artifacts such as batch effects, inconsistent, extreme, or suspicious values is critically important but simultaneously more challenging as the data dimension increases. New data visualization, detection, and correction or normalization methods have been adopted for HDD, as were described in section “IDA: Initial data analysis and preprocessing” of this overview. Techniques for data visualization and exploration such as those described in section “EDA: Exploratory data analysis” of this overview are also important to provide biological insights and support development of new scientific hypotheses from HDD. The initial steps and exploratory data analyses described in sections “IDA: Initial data analysis and preprocessing” and “EDA: Exploratory data analysis” are optimally performed when equipped with a good understanding of the data sources and data generation methods, for example assay technologies that produce omics data, and interpreted in collaboration with other scientists knowledgeable in the technology, biology, and clinical aspects.

Statistical analysis methods that were developed for traditional settings where the number of independent observations or subjects is substantially larger than the number of variables acquired are widely used by classically trained statisticians and others in a variety of applications, and their widespread use is supported by ready availability of software. Emergence of many new types of HDD has exposed the limitations of many traditional methods. Often, methods rely heavily on distributional assumptions such as normality, which may be unrealistic for data types generated by novel technologies such as omics assays. Many methods owe their robustness to such assumptions to large sample size, yet the notion of what qualifies as “large *n*” is dramatically different for HDD where even the most basic requirement *n* > *p* is not satisfied. Much traditional statistical methodology for addressing multivariate data has focused heavily on mathematically tractable joint distributions such as multivariate Gaussian or assumed that sample sizes were large enough that this served as a good approximation. As these are not reasonable assumptions for many types of HDD, many researchers opt for an alternative strategy of examining each of many variables one-at-a-time. Yet, naively taking such an approach is fraught with danger of generating many false discoveries due to the extremely large number of variables examined. Traditional strategies for controlling false positive findings, such as controlling the FWER, are often impractical or overly stringent in view of the goals of many studies involving HDD, and this recognition has stimulated development of novel approaches for false discovery control. Section “TEST: Identification of informative variables and multiple testing” of the overview highlighted some of these many challenges and summarized some useful strategies to address them.

The last few decades have seen substantial progress in development of prediction modelling methodology, especially as applicable to HDD, and increased availability of free software to implement these methods has fueled their use. Available methods include a variety of statistically based approaches as well as a number of purely algorithmic approaches such as many machine learning methods. Prediction models developed from HDD have intrigued many researchers under the impression that with sufficiently large volumes of data one should be capable of predicting virtually anything. Numerous dramatic claims of performance have been made; unfortunately, these claims do not always withstand careful scrutiny. Section “PRED: Prediction” provides a review of several popular prediction modelling methods for HDD, and it stressed the importance of following proper procedures to assess and avoid model overfitting that leads to prediction models that do not perform well outside of the data from which they were developed. Poor study design and faulty prediction modelling approaches that lead to spurious and overfitted models along with wildly inaccurate claims of their performance persist in the biomedical literature. Guidance provided in section “PRED: Prediction” aims to reduce this problem and promote successful development of useful prediction models.

Within the STRATOS initiative, there are currently nine topic groups (TGs), mainly concerned with LDD. Table 27 presents the relationship of the other STRATOS topic groups to TG9 group and how TG9 guidance will build upon that of other TGs to adapt it for relevance.

**Table 27:** Relationship and collaboration between TG9 (topic group 9) and the other topic groups of the STRATOS initiative

All other topic groups work on issues that are also relevant for the analysis of HDD. Obviously, all papers are written in the context of LDD. Appropriate study designs (TG5) are a key to improve research in the health sciences. It is well known that mistakes in design are often irremediable [229]. Nearly all studies in HDD and LDD have to cope with missing data (TG1, [58]) and data preprocessing is a relevant topic for all studies, closely related to tasks in initial data analysis (TG3). Analyzing LDD, the importance of IDA was largely ignored and a recent review showed that reporting of IDA is sparse [230]. In section “IDA: Initial data analysis and preprocessing,” we provided a discussion of IDA aspects in the context of HDD. Measurement error and misclassification (TG4) is a common problem in many studies in LDD and HDD, which is often ignored in practice [231]. Studies with a survival time output are popular in HDD, and they have to cope with several issues discussed in the survival analysis group (TG8, [232])
In the context of LDD, TG2 published a review focusing on approaches and issues for deriving multivariable regression models for description [136]. Although analyses of HDD concentrate more on models for prediction, some of the issues are also relevant and the very large number of variables and (too) small sample sizes strengthen some problems severely. In LDD, issues in deriving models for prediction are discussed in TG6 [233]. Finally, the overarching aim of many HDD studies is to discover knowledge that is causally related to an outcome of interest. However, causal inference imposes several important challenges (TG7, [234])

## Conclusions

This overview aimed to provide a solid statistical foundation for researchers, including statisticians and non-statisticians, who are newly embarking on research involving HDD or who are merely wanting to better evaluate and understand results of HDD analyses. Common approaches for the statistical analysis of high-dimensional biomedical data are described in 24 method tables; see Table 28 for a list of these tables. New methods to generate HDD or combine existing data resources to yield HDD will continue to evolve, and there will be continued need to develop new and improved computational and statistical analysis strategies to address new types of data and novel questions to be answered from those data. Basic concepts and strategies presented in this overview will remain relevant, and their wider grasp by the biomedical research community will hopefully lead to continued improvement in the quality, reliability, and value of studies involving HDD. Most importantly, strong collaborations between statisticians, computational scientists, and other biomedical researchers such as clinicians, public health experts, laboratorians, technology experts, bioinformaticians, and others that are relevant to each project, are essential to produce the most reliable and meaningful data and results.

**Table 28:** Overview of method tables with descriptions of statistical methods

1	Methods for visual inspection of univariate and multivariate distributions: Histograms, boxplots, scatterplots, correlograms, heatmaps (Table 2)
2	Methods for descriptive statistics: Measures for location and scale, bivariate measures, RLE plots, MA plots (Table 3)
3	Method for analysis of control values: Calibration curve (Table 4)
4	Methods for graphical displays: Principal component analysis (PCA), Biplot (Table 5)
5	Methods for background subtraction and normalization: Background correction, baseline correction, centering and scaling, quantile normalization (Table 6)
6	Methods for batch correction: ComBat, SVA (surrogate variable analysis) (Table 7)
7	Method for recoding: Collapsing categories (Table 8)
8	Method for filtering and exclusion of variables: Variable filtering (Table 9)
9	Method for construction of new variables: Discretizing continuous variables (Table 10)
10	Method for imputation of missing data: Multiple imputation (Table 11)
11	Methods for graphical displays: Multidimensional scaling, t-SNE, UMAP, neural networks (Table 12)
12	Methods for cluster analysis: Hierarchical clustering, k-means, PAM (Table 13)
13	Methods for estimation of the number of clusters: Scree plots, silhouette values (Table 14)
14	Methods for hypothesis testing for a single variable: *T*-test, permutation test (Table 15)
15	Methods for hypothesis testing for multiple variables in HDD: Limma, edgeR, DEseq2 (Table 16)
16	Methods for multiple testing corrections: Bonferroni correction, Holm’s procedure, Westfall-Young permutation procedure (Table 18)
17	Methods for multiple testing corrections controlling the FDR: Benjamini-Hochberg, *q*-values (Table 19)
18	Methods for multiple testing for groups of variables: Gene set enrichment analysis (GSEA), over-representation analysis, global test, topGO (Table 20)
19	Methods for variable transformations: Log-transform, standardization (Table 21)
20	Method for dimension reduction: Supervised principal components (Table 22)
21	Methods for statistical modelling with constraints on regression coefficients: Ridge regression, lasso regression, elastic net, boosting (Table 23)
22	Methods for statistical modelling with machine learning algorithms: Support vector machine, trees, random forests, neural networks and deep learning (Table 24)
23	Methods for assessing performance of prediction models: MSE, MAE, ROC curves, AUC, misclassification rate, Brier score, calibration plots, deviance (Table 25)
24	Methods for validation of prediction models: Subsampling, Cross-validation, Bootstrapping, use of external datasets (Table 26)

## Data Availability

Not applicable.

## Abbreviations

AIC: Akaike information criteria
ANOVA: Analysis of variance
asw: Average silhouette width
AUC: Area under the curve
Bagging: Bootstrap AGGregatING
BH: Benjamini-Hochberg
BIC: Bayesian information criteria
BMI: Body mass index
CAST: Cluster Affinity Search Technique
CLARA: Clustering Large Applications
ComBat: Methods for adjustment of batch effects
DESeq2: Differential gene expression analysis of RNA-seq data
EDA: Exploratory data analysis
EM: Expectation-maximization
EQUATOR: Enhancing the QUAlity and Transparency Of health Research
FDP: False discovery proportion
FDR: False discovery rate
FWER: Familywise error rate
GO: Gene ontology
GSEA: Gene set enrichment analysis
HDD: High-dimensional data
IDA: Initial data analysis
IOM: Institute of Medicine of the National Academy of Sciences, USA
LDD: Low-dimensional data
limma: Linear Models for Microarray Data
m/z: Mass-to-charge ratio
MA plot: Bland-Altman plot
MAD: Median absolute deviation to the median
MAE/MAPE: Mean absolute (prediction) error
MAR: Missing at random
MCAR: Missing “completely at random”
MDS: Multidimensional scaling
MNAR: Missing not at random
MSE/MSPE: Mean squared (prediction) error
NB: Negative binomial
nMDS: Non-Metric Multidimensional Scaling
PAM: Partitioning around medoids
PC: Principal component
PCA: Principal component analysis
PET: Positron emission tomography
PLS: Partial least squares
REMARK: REporting recommendations for tumor MARKer prognostic studies
RLE: Relative log expression
RMSE: Root mean squared error
ROC: Receiver operating characteristic
SNE: Stochastic neighbor embedding
SOMs: Self-organizing maps
STRATOS: STRengthening Analytical Thinking for Observational Studies
superPC: Supervised principal components
SVA: Surrogate variable analysis
SVM: Support vector machine
TG: Topic group
topGO: Topology-based Gene Ontology enrichment analysis
TRIPOD: Transparent Reporting of a multivariable prediction model for Individual Prognosis Or Diagnosis
t-SNE: T-Distributed Stochastic Neighbor Embedding
UMAP: Uniform Manifold Approximation and Projection
WHR: Waist-hip ratio
